# Synthesis and Application of a Hydrophobic Polyglutamate Bearing a Triphenylphosphine Group for the Orientation of Pharmaceutically Active Compounds and the Measurement of Residual Dipolar Couplings

**DOI:** 10.1002/mrc.5522

**Published:** 2025-04-20

**Authors:** Jan Rettig, Michael Gölz, Christina M. Thiele

**Affiliations:** ^1^ Clemens‐Schöpf‐Institut für Organische Chemie und Biochemie Technische Universität Darmstadt Darmstadt Germany

**Keywords:** alignment media, conformation, pharmaceuticals, relative configuration, residual dipolar couplings, structure elucidation

## Abstract

For structure elucidation purposes, it is highly desirable to partially retain anisotropic observables in high‐resolution NMR spectra, as they provide valuable information about the relative configuration, conformation, and dynamics of compounds in solution. Alignment media based on lyotropic liquid crystalline phases formed by α‐helical polypeptides can be used to achieve weak solute alignment. We present a novel homopolyglutamate bearing a bulky and hydrophobic triphenylphosphine side chain, which proved to be an excellent alignment medium using chloroform as a co‐solvent. We successfully applied the alignment medium for the measurement of residual dipolar couplings (RDCs) of artemisinin, an antimalarial drug, galantamine, which is used to treat Alzheimer's disease, and vincamine, a cerebral vasodilator and potential anti‐cancer agent. Excellent agreement between experimental and back‐calculated RDCs is obtained, and the enantio‐differentiating property of the new medium is demonstrated using the model compound isopinocampheol. Our results show that this alignment medium is of high interest for elucidating compounds characterized by high complexity and relevance in the research field of small molecule pharmaceuticals.

We present a novel homopolyglutamate‐based lyotropic liquid crystal, bearing a bulky and hydrophobic triphenylphosphine side chain. We successfully applied it as an alignment medium for measuring RDCs in artemisinin, an antimalarial drug, galantamine, used to treat Alzheimer's disease, and vincamine, a cerebral vasodilator and potential anticancer agent. Our results show that this alignment medium is of high interest for elucidating compounds characterized by high complexity and relevance in the research field of small molecule pharmaceuticals.

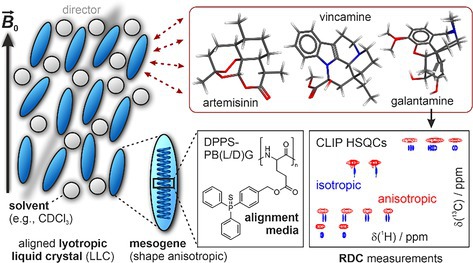

## 1 Introduction

If dissolved in a suitable, helicogenic solvent like chloroform, the homopolypeptide poly‐γ‐benzyl‐l/d‐glutamate (PB(l/d)G) is known to adopt an α‐helical conformation, stabilized by intramolecular hydrogen bonding [1, 2]. These helices exhibit a screw sense that depends on the centrochirality of the amino acid in the polymer backbone with the γ‐esters of l‐glutamic acid forming right‐handed (*P*) helices and the γ‐esters of d‐glutamic acid forming left‐handed (*M*) helices [3, 4]. This α‐helical secondary structure leads to a rigid rod‐like behavior causing shape anisotropy, which allows dissolved PB(L/D)G—and other polypeptides and polymer classes [5, 6]—to form lyotropic liquid crystalline (LLC) phases above a system‐specific critical concentration in helicogenic solvents [7, 8, 9, 10]. These LLC phases [11, 12, 13] have been studied as alignment media [10, 14, 15, 16, 17, 18, 19, 20, 21, 22, 23, 24, 25]. The mesogens of LLC‐based alignment media align relative to the external magnetic field [11, 12] and—if an analyte is added—can interact with this compound transferring this alignment partially onto the analyte [26]. This induces anisotropy in the tumbling and rotation of the analyte, making anisotropic NMR observables accessible for structure elucidation [27]. These anisotropic observables yield complementary global structural information to the established local isotropic observables, nuclear Overhauser effect (nOe) [28, 29, 30] or scalar coupling (*J*) [31, 32, 33]. The anisotropic NMR observables are the residual chemical shift anisotropy (RCSA) [34, 35, 36], residual quadrupolar couplings (RQCs) [24, 37], and residual dipolar couplings (RDCs) [5, 27, 38, 39], with this publication focusing on the latter.

**FIGURE 1:**
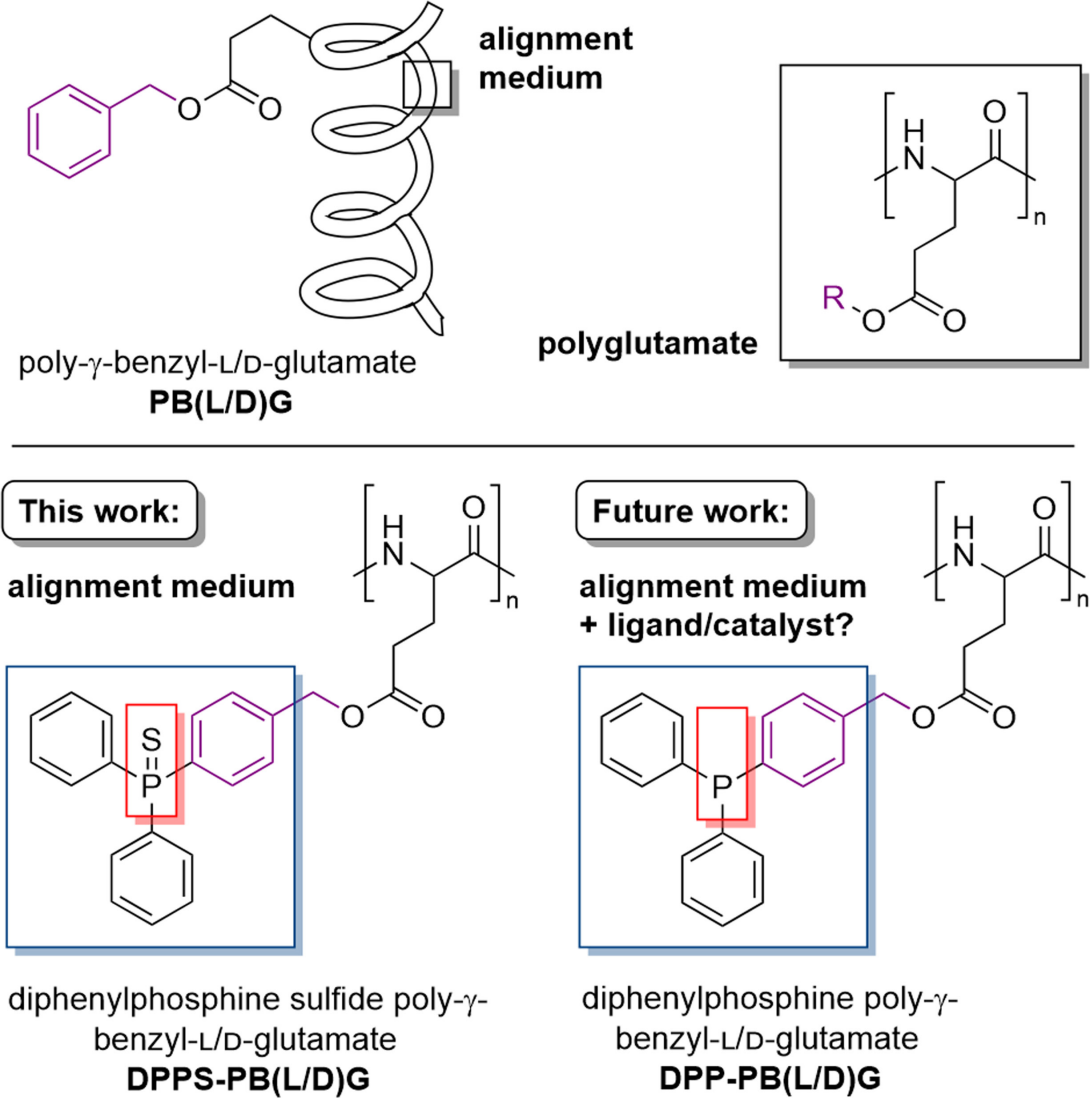
At the top, the well‐studied alignment medium PB(L/D)G and its α‐helical conformation are illustrated. On the bottom, the structure of the presented polymer diphenylphosphine sulfide poly‐γ‐benzyl‐l/d‐glutamate (DPPS‐PB(L/D)G, left) and the future synthesis goal diphenylphosphine poly‐γ‐benzyl‐l/d‐glutamate (DPP‐PB(L/D)G, right) are compared (see red boxes). Both have triphenylphosphine in the side chain and should be able to act as an alignment medium. Additionally, DPP‐PB(L/D)G could potentially act as a polymeric ligand in asymmetric transition metal catalysis [40, 41, 42, 43].

Especially sought‐after media are compatible with a wide range of analytes that allow the extraction of all possible one‐bond carbon‐hydrogen (^1^ *D*_CH_) RDCs of a given compound and exhibit excellent spectral quality with line widths comparable to the isotropic state. In this work, we present the synthesis of the new polymers diphenylphosphine sulfide poly‐γ‐benzyl‐l/d‐glutamate (DPPS‐PB(L/D)G), in which the benzyl ring in the sidechain of the previously mentioned PB(L/D)G is extended to a sulfur‐protected triphenylphosphine unit (see Figure 1).

By expanding the benzyl ring to triphenylphosphine, we hope to retain the necessary α‐helical conformation of PB(L/D)G in solution but alter the alignment properties compared with the parent polymer. The polyglutamate DPPS‐PB(L/D)G (Figure 1, bottom left) presented herein is the first result of a hybrid synthesis strategy in which we aim to synthesize polymers that can act not only as an alignment medium but potentially as a polymeric ligand for asymmetric transition metal catalysis [40, 41, 42, 43]. The latter could be achieved by removing the sulfur protection group (red boxes in Figure 1) from DPPS‐PB(L/D)G to yield the polymer diphenylphosphine poly‐γ‐benzyl‐l/d‐glutamate (DPP‐PB(L/D)G; Figure 1, bottom right). The sulfur‐protected motif in DPPS‐PB(L/D)G is chosen to avoid three potential challenges when synthesizing or applying the free triphenylphosphine, which is the unwanted oxidation of the phosphorus atom [44, 45], the high and diverse reactivity with analytes and reagents [46], and the inhibition of the ring‐opening polymerization of the *N*‐carboxy anhydride monomers (NCAs) [47, 48].

To demonstrate the broad applicability of the new polymers as an alignment medium for different compound classes, we measured RDCs not only for α‐santonin and isopinocampheol (IPC), which we use as a “proof‐of‐principle” compound, but also for three more complex pharmaceutical compounds. These are artemisinin, which is utilized to treat malaria [49, 50] and has been investigated using RDCs before [51, 52, 53, 54], vincamine, which is a cerebral vasodilator and a potential antitumor agent [55], and galantamine, which is used to treat Alzheimer's disease [56]. The latter is exciting because of its conformational flexibility, which we want to investigate using our new alignment medium.

## 2 Results

### 2.1 Polymer Synthesis and Characterization

**SCHEME 1:**
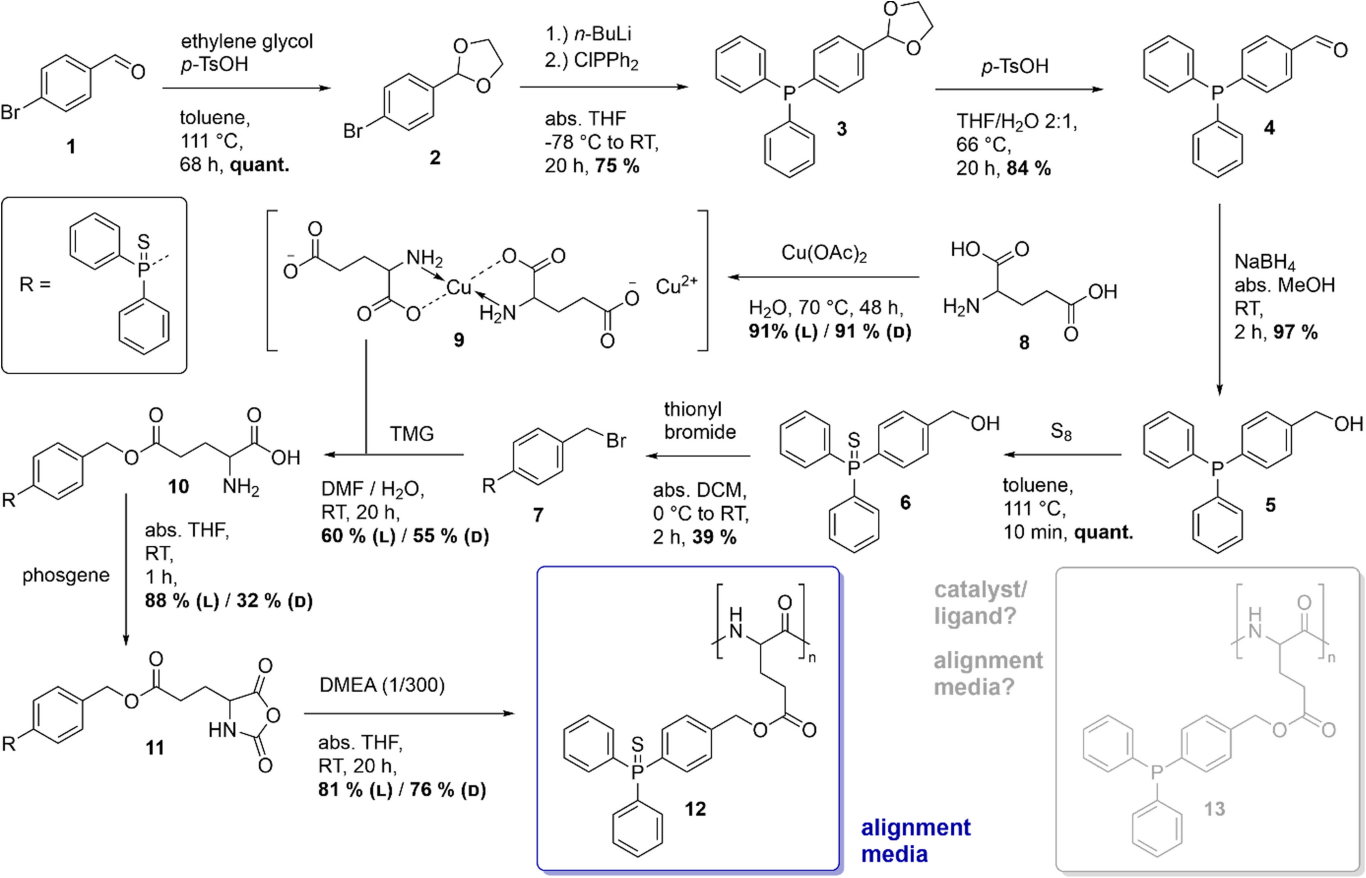
Synthesis of the sulfur‐protected DPPS‐PB(L/D)G **12** (blue box). The side chain synthesis starts from 4‐bromobenzaldehyde **1**, which is transformed into the triphenylphosphine bromide **7** over multiple steps. Compound **7** is then esterified with glutamic acid copper complexes **9** to yield γ‐glutamic acid esters **10** [57]. These are treated with phosgene to generate the corresponding *N‐*carboxy anhydrides (NCAs) **11** [58], which are polymerized using *N*,*N*‐dimethylethanolamine (DMEA) to yield the two enantiomeric alignment media **12** [47]. Detailed experimental procedures can be found in the Supporting Information. In the future, the polymers DPP‐PB(L/D)G **13** (grey box) can be synthesized from polymers **12**.

The synthesis of the alignment medium **12** is shown in Scheme 1.

The procedure is carried out over 10 steps, starting with 4‐bromobenzaldehyde **1**. The starting material is protected as an acetal **2** [59, 60, 61], which is reacted with diphenylchloride to yield triphenylphosphine **3** [59, 60, 61]. The acetal protecting group is hydrolyzed to give aldehyde **4** [60, 61]. The aldehyde **4** is reduced to alcohol **5** [62], which is then protected with elemental sulfur (**6**) [63, 64] and subsequently transformed into the bromide **7** [65]. At this point of the synthesis, the achiral compound **7** is esterified with the two different enantiomers of glutamic acid **8**, which are used as copper (II) complexes **9** [57] to ensure selective esterification of the amino acid side chain (γ‐esterification) [57, 66]. The esters **10** are treated with phosgene to transform them into *N*‐carboxy anhydrides (NCAs) **11** [58], which undergo ring‐opening polymerization initiated by DMEA to yield the polypeptides DPPS‐PB(L/D)G **12** [47] with number average molecular weights of $\bar{M_{n}}$ (**12**‐L) = 1.804·10^5^ g mol^−1^ and $\bar{M_{n}}$ (**12**‐D) = 2.343·10^5^ g mol^−1^ (for more details, see Supporting Information).

**FIGURE 2:**
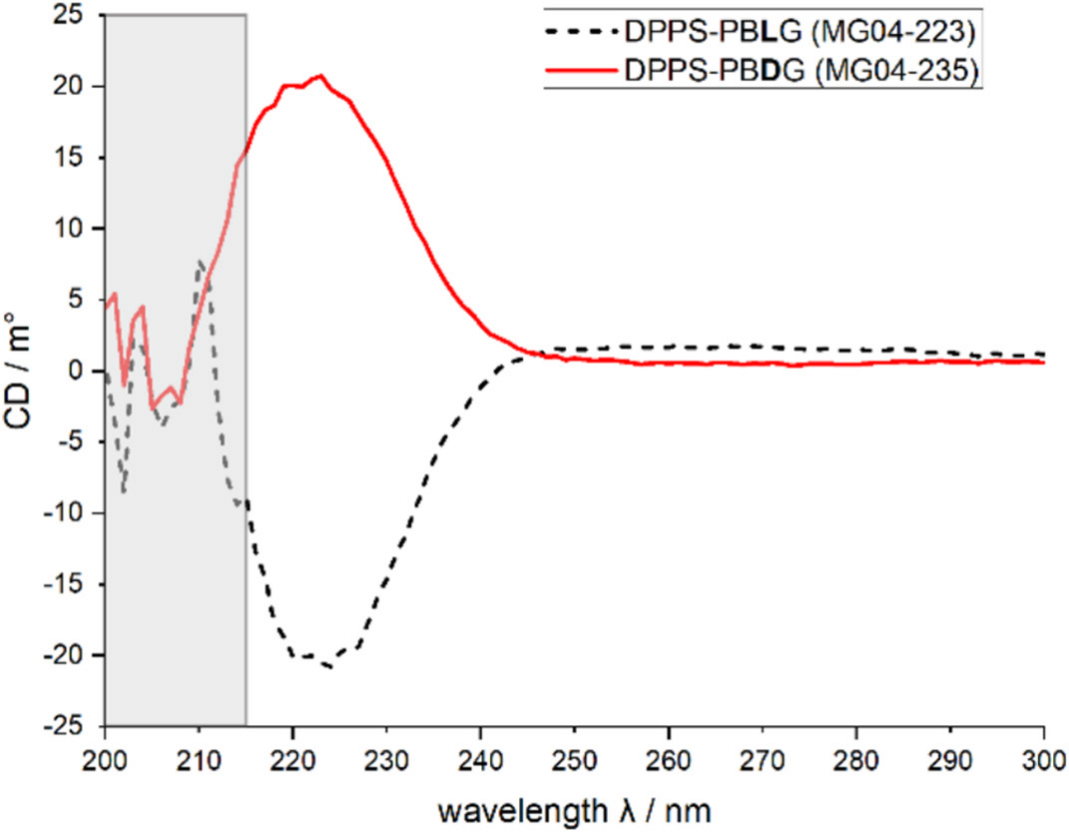
CD spectra of DPPS‐PBLG (dashed black line) and DPPS‐PBDG (solid red line) dissolved in chloroform (~2.5 mg/mL each). The measurements are performed in custom‐made demountable cuvettes with a path length of *d* ~ 0.01 mm [68]. For wavelengths shorter than 215 nm (grey box), the UV‐vis cut‐off of the solvent chloroform is reached. The rest of the spectrum exhibits the expected mirror‐image behavior.

After successful synthesis (see Scheme 1 and Supporting Information), we investigated the secondary structure of the new polymers DPPS‐PB(L/D)G, which is expected to adopt an α‐helical conformation. Therefore, we measured the polymers' circular dichroism (CD) spectra [67] in dilute chloroform solution, depicted in Figure 2.

DPPS‐PBLG (dashed black line) shows a negative CD signature (right‐handed helix), and DPPS‐PBDG (red solid lines) shows a positive CD signature (left‐handed helix) at around 225 nm. This indicates that both polymers adopt an α‐helical conformation with opposite handedness [69, 70]. The almost identical absolute values suggest these polymers behave like true enantiomers.

### 2.2 LLC Properties and RDCs of a Test Compound

**FIGURE 3:**
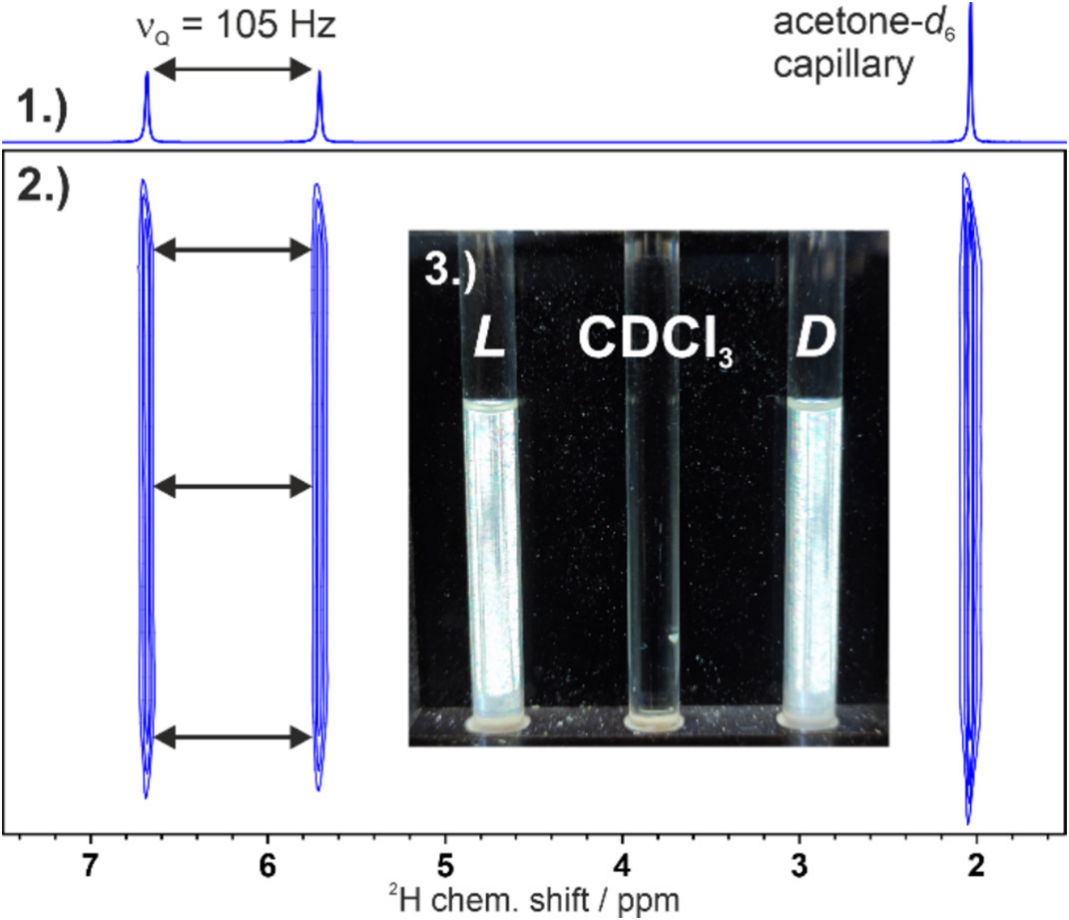
(1) ^2^H and (2) ^2^H‐image [71] spectra acquired at 107.5 MHz and 300 K using a 10.36% (w/w) sample of DPPS‐PBLG in CDCl_3_. (3) Photography of the LLC phases DPPS‐PBLG/CDCl_3_ (left) and DPPS‐PBDG/CDCl_3_ (right) between crossed polarization filters, both at a concentration of 10.5% (w/w) of polymer. In contrast to isotropic CDCl_3_ (middle), the phases show intense birefringence due to their liquid crystalline nature.

NMR samples of DPPS‐PB(L/D)G were prepared in CDCl_3_ to investigate the formation of LLC phases. The NMR tubes were placed between crossed polarization filters where they showed birefringence, indicating the formation of an LLC phase. ^2^H and ^2^H‐image [71] spectra were measured to monitor the quadrupolar splitting of the solvent signal, an indicator for the anisotropy and homogeneity of the sample (see Figure 3).

**FIGURE 4:**
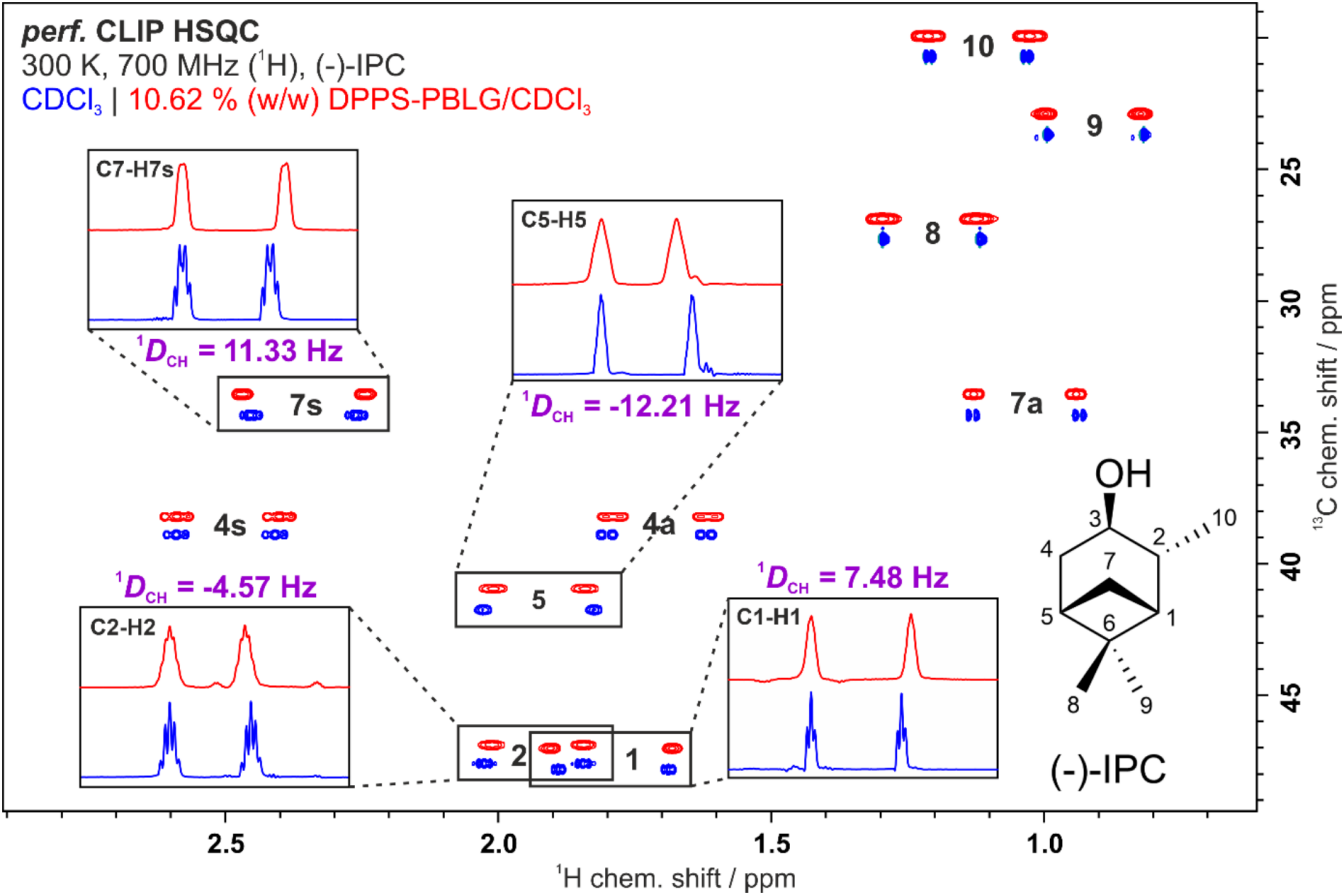
*Perfect* CLIP HSQC [76, 77] spectra of (−)‐IPC in isotropic CDCl_3_ (blue) and anisotropic DPPS‐PBLG/CDCl_3_ (red, 10.62% (w/w)) acquired at 700 MHz (^1^H frequency) and 300 K. The anisotropic spectrum is shifted both horizontally and vertically to allow direct comparison of both spectra and couplings. Some representative traces and RDCs (values in violet) are highlighted to demonstrate the high spectral quality. In each trace, the left‐most edges of the doublets were aligned to show the differences in coupling more clearly on the right‐hand side. All couplings can be found in the Supporting Information.

Both l‐ and d‐polymers formed LLC‐phases that showed the desired birefringence, a deuterium solvent signal fully split into a doublet by the quadrupolar splitting Δ*v*_Q,_ and good spatial homogeneity over the whole length of the active volume (22 mm) proven by the ^2^H‐image [71] spectrum. Determination of their critical concentrations (see Supporting Information) yielded results of about 9.0%–9.5% (w/w) at 300 K for both polymers. This is close to the critical concentration of structurally similar high‐molecular weight polymers used as alignment media [10, 69, 70, 72]. Encouraged by these preliminary results, samples of DPPS‐PB(L/D)G/CDCl_3_ with (−)‐IPC were prepared, and ^1^ *D*_CH_‐RDCs of the analyte were determined. To achieve this, F_1_‐coupled *J*‐scaled BIRD‐HSQCs [73, 74], their variant with multi‐quantum evolution (MQEvo) [75], and F_2_‐coupled *perfect* CLIP HSQCs [76, 77] were acquired in isotropic and anisotropic solutions to determine the scalar couplings (^1^ *J*_CH_) and the total couplings (^1^ *T*_CH_), respectively. RDCs are calculated using the expression ^1^ *T*_CH_ = ^1^ *J*_CH_ + 2 ^1^ *D*_CH_ [27]. F_2_‐coupled *perfect* CLIP HSQCs [76, 77] of (−)‐IPC in isotropic and anisotropic solutions are shown in Figure 4.

Figure 4 shows an excellent spectral quality with symmetrical line shapes and only slightly broadened signals in the anisotropic state (red) compared with the isotropic state (blue). The ^1^ *D*_CH_ RDCs for (−)‐IPC in 10.62% (w/w) DPPS‐PBLG/CDCl_3_ range from −12 to +11 Hz and for (−)‐IPC in 10.67% (w/w) DPPS‐PBDG/CDCl_3_ from −11 to +9 Hz. For the analysis of the RDCs, the order tensor approach is applied, in which the measured experimental RDCs (*D*_exp_) and a structural model (single conformation) are used to form a set of linear equations. This is solved via singular value decomposition (SVD) to determine the alignment tensor [26, 78, 79]. The experimental RDCs are weighted by their experimental error so that RDCs with low uncertainties contribute more to the fitting procedure than RDCs extracted with a lower accuracy. The best‐fitting solution of this overdetermined set of linear equations is then determined to obtain the calculated RDCs (*D*_calc_). To perform these tasks, the software RDC@hotFCHT [80, 81] is applied. For evaluation of the quality of the RDC data, the experimental and calculated RDCs are compared using statistical measures like the quality factor *Q* as defined by Cornilescu [82], which should have values close to zero for structural models that are represented well by the experimental RDCs. This treatment employing a single structure is called *single‐conformer‐single‐tensor* fit (SCST) [78, 81] and is only valid if the compound behaves as one rigid domain or entity and thus can be described by one single conformer, which is true for the bridged and well‐studied hydrocarbon IPC. Analysis of the RDCs shows that DPPS‐PB(L/D)G/CDCl_3_ allows the extraction of RDCs with high accuracy and small errors, which is confirmed by excellent quality factors of *Q* ≤ 0.04 when fitting the RDCs to the structure of (−)‐IPC [20]. Since DPPS‐PBLG and DPPS‐PBDG are synthesized from homochiral but enantiomeric monomers, it was also investigated whether they allow enantiodifferentiation by diastereomorphous interactions [20, 23]. Comparing the orientation tensors of (−)‐IPC in DPPS‐PBLG and DPPS‐PBDG in CDCl_3_ with each other, the so‐called (5D) *β*‐angle [83, 84] of 12° is found, where 0° corresponds to no observable enantiodifferentiation and a *β*‐angle of 90° is the maximum enantiodifferentiation [85].^1^ The observed value of 12° is of similar magnitude as previously observed for (−)‐IPC in PBLG versus PBDG in CDCl_3_, which gives *β*‐angles of 8° [72].

### 2.3 RDCs of Pharmaceutically Active Compounds

**FIGURE 5:**
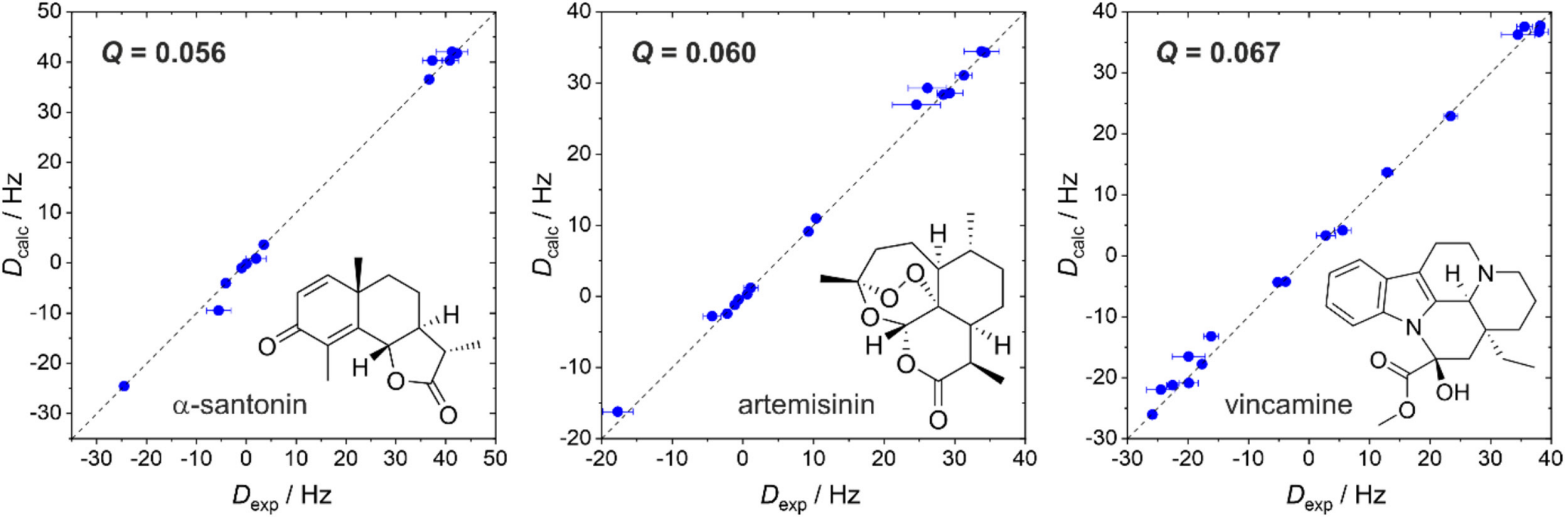
Comparison of experimental (*x*‐axis) and back‐calculated RDCs (*y*‐axis) for α‐santonin, artemisinin, and vincamine (from left to right). Experimental errors are shown as horizontal bars. Note that some errors are too small to be depicted here. All experimental RDCs presented here are determined via F_2_‐coupled CLIP/CLAP HSQCs [76] from LLC‐phases of the polymer DPPS‐PBDG. The quality factor *Q* of each fit and a Kekulé structure are given. All coordinates, coupling data, and fit results can be found in the Supporting Information.

Encouraged by the first results using the thoroughly studied terpene IPC, we wanted to test our new alignment medium with more complex and pharmaceutically relevant compounds. The four compounds chosen are α‐santonin, artemisinin, vincamine, and galantamine. Of these, the first three are rigid compounds having only one populated low‐energy conformer, whereas galantamine exhibits some conformational flexibility. The rigid lactones α‐santonin [86, 87, 88] and artemisinin [51, 52, 53, 54], the latter having an additional peroxide bridge, have been investigated before using RDCs. To the best of our knowledge, vincamine has not been the subject of RDC analysis, making the investigation even more interesting. Structurally, vincamine is an indole alkaloid with an additional tertiary amine, thus providing additional functional groups to the ester (lactones α‐santonin and artemisinin) and the alcohol group (IPC) to demonstrate the broad tolerance of functional groups of DPPS‐PBLG. The structures and the correlation of experimental and back‐calculated RDCs of the three rigid compounds are illustrated in Figure 5. Due to the rigidity of the structures, the analysis of RDCs is performed the same way as described for IPC before (SCST).

Although the ^1^ *D*_CH_ RDCs for the quite strongly oriented α‐santonin are fairly large, ranging from −24 up to 42 Hz, the *Q*‐factor has an excellent value of 0.056, indicating the adequacy of the structural model and a high accuracy of the determined RDCs. The latter finding is quite surprising since a high degree of orientation often increases the influence of systematic errors like strong proton‐proton coupling phenomena on the accuracy of experimental ^1^ *D*_CH_ RDCs, deteriorating the resulting RDC fit. Fortunately, this is not the case for the alignment medium–analyte combination studied here.

The extracted ^1^ *D*_CH_ RDCs for artemisinin range from −18 to 34 Hz, and their fit resulted in a *Q*‐factor of 0.060 for the correct configuration of artemisinin using ^1^ *D*_CH_ RDCs exclusively [51, 52].

The extracted ^1^ *D*_CH_ RDCs range from −26 to 38 Hz for vincamine, resulting in a *Q*‐factor of 0.067. For the fitting procedure, only RDCs of the rigid core of the compound were used, ignoring the two flexible side chains (methyl ester and ethyl group) in the analysis.

The RDC fits presented in Figure 5 show an excellent agreement for all three compounds studied, indicating that DPPS‐PB(L/D)G is a suitable alignment medium for the orientation of all three rigid compounds and the determination of high‐quality RDC data.

To test the utility of our new alignment medium even further, we studied the compound galantamine, which has multiple functional groups and some conformational flexibility. We chose it as test case because flexibility and its proper treatment is becoming more relevant since pharmaceutical research is increasingly interested in flexible small‐molecule drugs that are at the edge or beyond the rule‐of‐5 chemical space [89]. There are examples in the literature that are significantly more flexible than galantamine. Their ability to adopt multiple different conformations makes the determination of their structures a real challenge [90, 91]. Here RDCs could provide important additional structural information in the future. Showing that galantamine can be oriented and analyzed with the new medium is thus a first step.

For the analysis of the conformational space of galantamine, the open‐source program Conformer‐Rotamer Ensemble Sampling Tool (CREST) [92] was used in combination with the program ORCA [93, 94] for DFT optimization (hybrid functional B3LYP [95, 96, 97], Pople‐type basis set 6‐311+G(d) [98, 99]; for further details, see Supporting Information). The results of the conformational analysis are summarized in Table 1.

**TABLE 1:** Relative energies *E*_rel_ (col. 2) and Boltzmann‐weighted conformer populations *p*_x, CREST_ (col. 3) of conformers 1–6 (col. 1) directly from CREST (for more details, see Supporting Information, section 7). These populations are transformed into combined populations of conformers *p*_x, comb_, which exhibit the same conformation for the compound's parts used for RDC analysis (col. 4). The combined populations are normalized *p*_x, norm_ (to give 100%) in col. 5.

Conf	*E*_rel_ (kcal)	*p*_x, CREST_	*p*_x, comb_.	*p*_x, norm_
**01**	0.000	0.4881	0.8944	**0.9393**
**02**	0.108	0.4064		
**03**	1.496	0.0391	0.0578	**0.0607**
**04**	1.935	0.0187		
05	2.258	0.0048	—	—
06	2.449	0.0052	—	—
—	—	—	—	—

Conformers 1 and 2, which are very close in relative energy, only differ in the conformation of the methoxy group at the aromatic ring. Since this group is not part of the RDC analysis, conformers 1 and 2 are treated equally. The same is true for conformers 3 and 4, which differ from conformers 1 and 2 in the conformations of the aliphatic five‐ and six‐membered rings (for superimposed conformers, see Supporting Information). For simplicity, we neglected all conformers of higher energies (*E*_rel_ > 2.2 kcal and *p*_x_ < 1%), which comprise roughly 5% of the conformational space, for further analysis.

**FIGURE 6:**
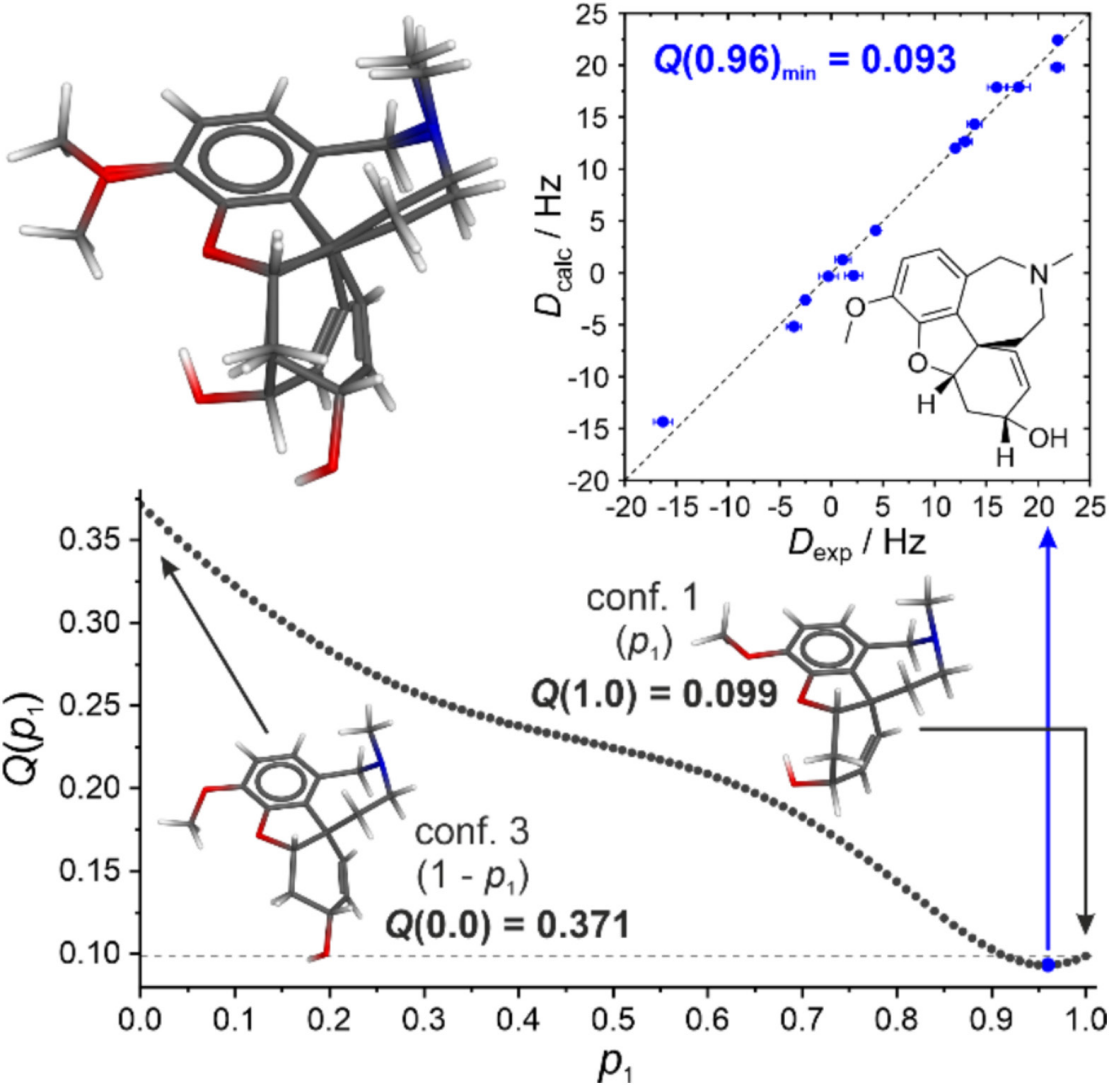
Results of the ^1^ *D*_CH_ RDC analysis for galantamine in 9.32% (w/w) of DPPS‐PBDG/CDCl_3_ (300 K) using two conformers: conformer 1 and conformer 3. The two conformers are overlayed at the aromatic ring in the upper left corner. This superposition of conformers is shown to visualize the differences between the two conformers, while the Eckart transformation [102, 105, 106] was chosen for the superposition of conformers during fitting (not depicted). On the bottom, the dependence of the *Q*‐factor on the population of conformer 1 is given (*Q*(*p*_1_)). The two conformers with their respective *Q*‐factors (*Q*(1.00) and *Q*(0.00)) are shown individually. The lowest *Q*‐factor is obtained for a population of 96% of conformer 1 (*Q*(0.96)_min_ = 0.093, blue dot), and the respective plot of the experimental vs. the calculated RDCs is shown in the upper right corner.

The two conformers remaining, which have normalized Boltzmann populations (see col. 5 in Table 1) of ~94% for the lowest energy conformer (conformer 1) and ~6% for the higher energy conformer (conformer 3; see Table 1, Supporting Information, sections 2.6 and 7), are chosen for RDC analysis. To properly account for the differences in internal dynamics of different conformationally flexible parts of the analyte, a structure model is usually constructed as a population‐weighted conformer ensemble [27]. Ideally, the alignment of each conformer would be represented by its own order tensor, requiring a (usually prohibitively) large number of experimental RDCs (*multiconformer–multitensor* approach [MCMT]) [100, 101]. If it can be assumed that conformer interconversion is faster than molecular tumbling or interconverting structures are similar [102, 103, 104], a single common order tensor describes the alignment of all conformers expressed in a common reference frame (Eckart frame) [102, 105, 106], leading to the *multiconformer–single‐tensor* approach (MCST) [78, 100]. The MCST approach allows for finding the populations that exhibit the best correlation between experimental and calculated RDCs and comparing these results to quantum‐chemical calculations or other analytical methods. This is applied for galantamine [78]. The results of the MCST RDC analysis are illustrated in Figure 6.

The ^1^ *D*_CH_ RDC fit for single conformer 1 (*p*_1_ = 1.00) results in a *Q*‐factor of 0.099, and for single conformer 3 (*p*_1_ = 0.00) in a significantly worse *Q*‐factor of 0.371. If the *Q*‐factor of the conformer ensemble (MCST fit) is plotted against the conformer population, a minimal *Q*‐factor is obtained for a population of 96% of conformer 1. The result of the ensemble fit at *Q*(0.96)_min_ = 0.093 is a small but significant improvement over the *Q*‐factor of the single conformer fit of conformer 1 (*p*_1_ = 1.00). Overall, the plot of the experimental RDCs (*x*‐axis) against the calculated RDCs (*y*‐axis) shows an excellent correlation with no RDC deviations outside their experimental errors. Thus, the RDCs of galantamine extracted from an LLC phase of DPPS‐PBDG/CDCl_3_ fit well to structural models and allow us to analyze the conformational space of galantamine using an MCST fit. The population determined (96% of conformer 1) compares exceptionally well to the population predicted by CREST (94% of conformer 1, normalized population), giving us confidence in the accuracy of the RDC analysis when using our new alignment medium.

## 3 Conclusion

We tested the polyglutamates containing sulfur‐protected triphenylphosphine (DPPS‐PB(L/D)G) as a novel LLC alignment medium for structure elucidation. After synthesis and confirmation that the helical polymers DPPS‐PB(L/D)G form stable LLC phases in chloroform, we found excellent spectral quality using the terpene IPC in “proof‐of‐principle” RDC measurements. Furthermore, we showed our new alignment medium's broad applicability with three rigid, more challenging, and pharmaceutically relevant compounds: α‐santonin, artemisinin, and vincamine. For each of them, all possible ^1^ *D*_CH_‐RDCs were obtained with high accuracy. Since more flexible small‐molecule drugs are increasingly interesting for pharmaceutical research, we applied our alignment medium for the compound galantamine, a drug used to treat Alzheimer's disease. Exploration of the conformational space using CREST yielded two relevant conformers, which mainly differ in ring conformations. ^1^ *D*_CH_ RDCs of galantamine could be extracted from spectra showing high spectral quality. The ensemble fit (MCST) of these RDCs improved the correlation of experimental and back‐calculated RDCs compared with the individual single‐conformer fits. The populations determined experimentally match the populations predicted by CREST.

The results presented herein highlight the properties of DPPS‐PB(L/D)G/CDCl_3_ as an excellent new alignment medium: It is compatible with an apolar organic solvent and multiple analytes of different functional groups, shapes, and degrees of conformational flexibility. Measurements in the anisotropic samples exhibit excellent spectral quality necessary to extract as many ^1^ *D*_CH_ RDCs as possible and to facilitate advanced RDC analyses. This will become increasingly important when RDC‐data‐driven algorithms for de novo [107, 108] or *refinement*‐based [109, 110, 111, 112, 113, 114, 115, 116] structure elucidation become the norm.

## 4 Experimental Section/Methods

Experimental details regarding the synthesis of the polymer, NMR measurements, RDC fits, coordinates, abbreviations/symbols, and computational details can be found in the Supporting Information.

### Peer Review

The peer review history for this article is available at https://www.webofscience.com/api/gateway/wos/peer‐review/10.1002/mrc.5522.

## Supporting information


**Figure S1:** 2H spectra measured during stepwise dilution of the samples to determine the critical concentration of DPPS‐PB(L/D)G in CDCl3 at 300 K and 107.5 MHz (700 MHz proton frequency). The full width at half maximum (FWHM, in Hz) of the right line of the doublet is given in gray. The values are determined using the TopSpin command peakw.
**Table S2:** Composition of the anisotropic analyte samples (LLC phases) used for RDC analysis.
**Table S3:** Additional spectra parameters that do not have a standard value.
**Figure S2:** Superimposed (at the aromatic ring) structural models of the different conformers of galantamine. On the left side, conformers 1 and 2 are overlaid; on the right, conformers 3 and 4, and in the middle, all four conformers. The figure shows that conformers 1 and 2 differ only for the methoxy group (C18), irrelevant to RDC analysis, and can be treated as the same conformer. The same is the case for conformers 3 and 4. The middle part shows that conformers 1 (+2) and 3 (+4) differ in the conformation of the six‐ (C9‐C12) and sevenmembered (C13, C14, N15, C16) rings.
**Figure S3:** 1H‐NMR (red, 600 MHz) and 13C‐NMR (blue, 151 MHz) of bromide **7** measured in CDCl3 at 300 K.
**Figure S4:** 31P‐NMR (green, 243 MHz) of bromide **7** measured in CDCl3 at 300 K.
**Figure S5:** ATR‐IR spectrum (neat) of bromide **7**.
**Figure S6:** HR‐MS (ESI positive) spectrum of bromide **7**. The simulated/expected isotope pattern is compared with the experimental results.
**Figure S7:** 1H‐NMR (red, 700 MHz) and 13C‐NMR (blue, 176 MHz) of ester (L)‐**10** measured in DMSO‐*d*6 + DCl at 300 K.
**Figure S8:** 31P‐NMR (green, 283 MHz) of ester (L)‐**10** measured in DMSO‐*d*6 + DCl at 300 K.
**Figure S9:** ATR‐IR spectrum (neat) of ester (L)‐**10**.
**Figure S10:** HR‐MS (ESI positive) spectrum of ester (L)‐**10**. The simulated/expected isotope pattern is compared with the experimental results.
**Figure S11:** 1H‐NMR (red, 600 MHz) and 13C‐NMR (blue, 151 MHz) of ester (D)‐**10** measured in DMSO‐*d*6 + DCl at 300 K. For the 13C{1H} spectrum, an exponential line broadening of 3 Hz was applied to improve the signal‐to‐noise ratio.
**Figure S12:** ATR‐IR spectrum (neat) of ester (D)‐**10**.
**Figure S13:** HR‐MS (ESI positive) spectrum of ester (D)‐**10**. The simulated/expected isotope pattern is compared with the experimental results.
**Figure S14:** 1H‐NMR (red, 600 MHz) and 13C‐NMR (blue, 151 MHz) of NCA (L)‐**11** measured in THF‐*d*8 at 300 K.
**Figure S15:** 31P‐NMR (green, 243 MHz) of NCA (L)‐**11** measured in THF‐*d*8 at 300 K.
**Figure S16:** ATR‐IR spectrum (neat) of NCA (L)‐**11**.
**Figure S17:** HR‐MS (ESI positive) spectrum of NCA (L)‐**11**. The simulated/expected isotope pattern is compared with the experimental results.
**Figure S18:** 1H‐NMR (red, 600 MHz) and 13C‐NMR (blue, 151 MHz) of NCA (D)‐**11** measured in THF‐*d*8 at 300 K.
**Figure S20:** ATR‐IR spectrum (neat) of NCA (D)‐**11**.
**Figure S21:** HR‐MS (ESI positive) spectrum of NCA (D)‐**11**. The simulated/expected isotope pattern is compared with the experimental results.
**Figure S22:** 1H‐NMR (red, 700 MHz) and 13C‐NMR (blue, 176 MHz) of polymer DPPSPBLG (L)‐**12** measured in THF‐*d*8 + TFA‐*d* at 300 K.
**Figure S23:** 31P‐NMR (green, 283 MHz) of polymer DPPS‐PBLG (L)‐**12** measured in THF‐*d*8 + TFA‐*d* at 300 K.
**Figure S24:** ATR‐IR spectrum (neat) of polymer DPPS‐PBLG (L)‐**12**
**Figure S25:** MALDI‐TOF‐MS spectrum of polymer DPPS‐PBLG (L)‐**12**. The mass difference between peaks (in red) corresponds to the molecular weight of the repeating unit.
**Figure S26:** SEC graph of DPPS‐PBLG (internal batch number MG04–223). Detailed information about the acquisition parameters can be found in SI section 2.2.
**Figure S27:** On the left side, the CD spectrum of DPPS‐PBLG dissolved in chloroform (2.5 mg/mL), and on the right side, the respective absorbance spectrum is shown. The measurement is performed in custom‐made demountable cuvettes with a path length of *d* ~ 0.01 mm. [5] At around 215 nm, the UV solvent cut‐off for chloroform is reached (visible on the right).
**Figure S28:** 1H‐NMR (red, 700 MHz) and 13C‐NMR (blue, 176 MHz) of polymer DPPSPBDG (D)‐**12** measured in THF‐*d*8 + TFA‐*d* at 300 K.
**Figure S29:** ATR‐IR spectrum (neat) of polymer DPPS‐PBDG (D)‐**12**.
**Figure S30:** MALDI‐TOF‐MS spectrum of polymer DPPS‐PBDG (D)‐**12**. The mass difference between peaks (in red) corresponds to the molecular weight of the repeating unit.
**Figure S31:** SEC graph of DPPS‐PBDG (internal batch number MG04–235). Detailed information about the acquisition parameters can be found in SI section 2.2.
**Figure S32:** On the left side, the CD spectrum of DPPS‐PBDG dissolved in chloroform (2.5 mg/mL), and on the right side, the respective absorbance spectrum is shown. The measurement is performed in custom‐made demountable cuvettes with a path length of *d* ~ 0.01 mm. [5] At around 215 nm, the UV solvent cut‐off for chloroform is reached (visible on the right).
**Figure S33:** (−)‐Isopinocampheol (IPC) with numbered atom positions. The diastereotopic CH2 protons are labeled 4 s, 4a, 7 s, and 7a, with ‘s’ defined as *syn* and ‘a’ as *anti* relative to the dimethyl bridge (C6, C8, C9). [14]
**Table S4:** Assignment of (−)‐IPC in CDCl3 at 700 MHz (1H freq.) and 300 K. [14, 46, 47]
**Table S5:** Scalar couplings (1*J*CH) of (−)‐IPC in CDCl3 at 700 MHz (1H freq.) and 300 K.
**Table S6:** Total couplings (1*T*CH) of (−)‐IPC in 10.62% (w/w) DPPS‐PBLG/CDCl3 at 700 MHz (1H freq.) and 300 K.
**Table S7:** Total couplings (1*T*CH) of (−)‐IPC in 10.67% (w/w) DPPS‐PBDG/CDCl3 at 700 MHz (1H freq.) and 300 K.
**Table S8:** RDCs (1*D*CH/1*D*CC) of (−)‐IPC calculated from the scalar couplings (1*J*CH) in isotropic CDCl3 (**Table S5**) and the total couplings (1*T*CH) in 10.62% (w/w) DPPS‐PBLG/CDCl3 (**Table S6**) at 700 MHz (1H freq.) and 300 K.
**Table S9:** RDCs (1*D*CH/1*D*CC) of (−)‐IPC calculated from the scalar couplings (1*J*CH) in isotropic CDCl3 (**Table S5**) and the total couplings (1*T*CH) in 10.67% (w/w) DPPS‐PBDG/CDCl3 (**Table S7**) at 700 MHz (1H freq.) and 300 K.
**Figure S34:** (−)‐α‐Santonin with numbered atom positions.
**Table S10:** Assignment of α‐santonin in CDCl3 at 700 MHz (1H freq.) and 300 K. [48]
**Table S11:** Scalar couplings (1*J*CH) of α‐santonin in CDCl3 at 700 MHz (1H freq.) and 300 K.
**Table S12:** Total couplings (1*T*CH) of α‐santonin in 9.41% (w/w) DPPS‐PBDG/CDCl3 at 700 MHz (1H freq.) and 300 K.
**Table S13:** RDCs (1*D*CH/1*D*CC) of α‐santonin calculated from the scalar couplings (1*J*CH) in isotropic CDCl3 (**Table S11**) and the total couplings (1*T*CH) in 9.41% (w/w) DPPSPBDG/ CDCl3 (**Table S12**) at 700 MHz (1H freq.) and 300 K.
**Figure S35:** Artemisinin with numbered atom positions.
**Table S14:** Assignment of artemisinin in CDCl3 at 700 MHz (1H freq.) and 300 K. [49]
**Table S15:** Scalar couplings (1*J*CH) of artemisinin in CDCl3 at 700 MHz (1H freq.) and 300 K.
**Table S16:** Total couplings (1*T*CH) of artemisinin in 9.61% (w/w) DPPS‐PBDG/CDCl3 at 700 MHz (1H freq.) and 300 K.
**Table S17:** RDCs (1*D*CH/1*D*CC) of artemisinin calculated from the scalar couplings (1*J*CH) in isotropic CDCl3 (**Table S15**) and the total couplings (1*T*CH) in 9.61% (w/w) DPPSPBDG/ CDCl3 (**Table S16**) at 700 MHz (1H freq.) and 300 K.
**Figure S36:** (+)‐Vincamine with numbered atom positions.
**Table S18:** Assignment of vincamine in CDCl3 at 700 MHz (1H freq.) and 300 K. [50]
**Table S19:** Scalar couplings (1*J*CH) of vincamine in CDCl3 at 700 MHz (1H freq.) and 300 K.
**Table S20:** Total couplings (1*T*CH) of vincamine in 9.86% (w/w) DPPS‐PBDG/CDCl3 at 700 MHz (1H freq.) and 300 K.
**Table S21:** RDCs (1*D*CH/1*D*CC) of vincamine calculated from the scalar couplings (1*J*CH) in isotropic CDCl3 (**Table S19**) and the total couplings (1*T*CH) in 9.86% (w/w) DPPSPBDG/ CDCl3 (**Table S20**) at 700 MHz (1H freq.) and 300 K.
**Figure S37:** (−)‐galantamine with numbered atom positions.
**Table S22:** Assignment of (−)‐galantamine in CDCl3 at 700 MHz (1H freq.) and 300 K. [51, 52]
**Table S23:** Scalar couplings (1*J*CH) of (−)‐galantamine in CDCl3 at 700 MHz (1H freq.) and 300 K. **Table S24:** Total couplings (1*T*CH) of (−)‐galantamine in 9.32% (w/w) DPPS‐PBDG/CDCl3 at 700 MHz (1H freq.) and 300 K.
**Table S25:** RDCs (1*D*CH/1*D*CC) of (−)‐galantamine calculated from the scalar couplings (1*J*CH) in isotropic CDCl3 (**Table S23**) and the total couplings (1*T*CH) in 9.32% (w/w) DPPSPBDG/CDCl3 (**Table S24**) at 700 MHz (1H freq.) and 300 K.
**Table S26:** Key parameters of the SVD‐based RDC fitting (using RDC@hotFCHT) of (−)‐IPC oriented in 10.67 w% DPPS‐PBDG/CDCl3 at 300 K and 700 MHz 1H frequency. The 1*D*CX RDCs are determined via F2‐coupled CLIP/CLAP HSQC spectra.
**Table S27:** Key parameters of the SVD‐based RDC fitting (using RDC@hotFCHT) of (−)‐IPC oriented in 10.67 w% DPPS‐PBDG/CDCl3 at 300 K and 700 MHz 1H frequency. The 1*D*CX RDCs are determined via a combination of F1‐coupled HSQC (CH and CH3) and F1‐coupled HSQC spectra with MQ evolution (CH2).
**Table S28:** Key parameters of the SVD‐based RDC fitting (using RDC@hotFCHT) of (−)‐IPC oriented in 10.62 w% DPPS‐PBLG/CDCl3 at 300 K and 700 MHz 1H frequency. The 1*D*CX RDCs are determined via F2‐coupled CLIP/CLAP HSQC spectra.
**Table S29:** Key parameters of the SVD‐based RDC fitting (using RDC@hotFCHT) of (−)‐IPC oriented in 10.62 w% DPPS‐PBLG/CDCl3 at 300 K and 700 MHz 1H frequency. The 1*D*CX RDCs are determined via a combination of F1‐coupled HSQC (CH and CH3) and F1‐coupled HSQC spectra with MQ evolution (CH2).
**Table S30:** Key parameters of the SVD‐based RDC fitting (using RDC@hotFCHT) of α‐santonin oriented in 9.41 w% DPPS‐PBDG/CDCl3 at 300 K and 700 MHz 1H frequency. The 1*D*CX RDCs are determined via F2‐coupled CLIP/CLAP HSQC spectra.
**Table S31:** Key parameters of the SVD‐based RDC fitting (using RDC@hotFCHT) of α‐santonin oriented in 9.41 w% DPPS‐PBDG/CDCl3 at 300 K and 700 MHz 1H frequency. The 1*D*CX RDCs are determined via a combination of F1‐coupled HSQC (CH and CH3) and F2‐ coupled CLIP/CLAP HSQC (CH2) spectra.
**Table S32:** Key parameters of the SVD‐based RDC fitting (using RDC@hotFCHT) of artemisinin oriented in 9.61 w% DPPS‐PBDG/CDCl3 at 300 K and 700 MHz 1H frequency. The 1*D*CX RDCs are determined via F2‐coupled CLIP/CLAP HSQC spectra.
**Table S33:** Key parameters of the SVD‐based RDC fitting (using RDC@hotFCHT) of (+)‐vincamine oriented in 9.86 w% DPPS‐PBDG/CDCl3 at 300 K and 700 MHz 1H frequency. The 1*D*CX RDCs are determined via F2‐coupled CLIP/CLAP HSQC spectra.
**Table S34:** Key parameters of the SVD‐based RDC fitting (using RDC@hotFCHT) of (−)‐galantamine oriented in 9.32 w% DPPS‐PBDG/CDCl3 at 300 K and 700 MHz 1H frequency. The 1*D*CX RDCs are determined via F2‐coupled CLIP/CLAP HSQC spectra. RDCs are fit in a **single‐conformer‐single‐tensor approach (SCST)** to the structure model of conformer 1.
**Table S35:** Key parameters of the SVD‐based RDC fitting (using RDC@hotFCHT) of (−)‐galantamine oriented in 9.32 w% DPPS‐PBDG/CDCl3 at 300 K and 700 MHz 1H frequency. The 1*D*CX RDCs are determined via F2‐coupled CLIP/CLAP HSQC spectra. RDCs are fit in a **single‐conformer‐single‐tensor approach (SCST)** to the structure model of conformer 3.
**Table S36:** Key parameters of the SVD‐based RDC fitting (using RDC@hotFCHT) of (−)‐galantamine oriented in 9.32 w% DPPS‐PBDG/CDCl3 at 300 K and 700 MHz 1H frequency. The 1*D*CX RDCs are determined via F2‐coupled CLIP/CLAP HSQC spectra. RDCs are fit in a **multi‐conformer‐single‐tensor approach (MCST)**. Data for the minimum of 96% conformer 1 and 4% conformer 3 is shown.

## Data Availability

The data that support the findings of this study are openly available in Zenodo at https://doi.org/10.5281/zenodo.14170603.

## Endnotes

^1^
For a discussion on the valid ranges and maxima of 5D beta angles in the context of enantifferentation, see reference [85] and the derivation in the Supporting Information of that reference.
