# Supplementary material for: Synthesis and Application of a Hydrophobic Polyglutamate Bearing a Triphenylphosphine Group for the Orientation of Pharmaceutically Active Compounds and the Measurement of Residual Dipolar Couplings
Source: Magn Reson Chem. 2025 Apr 20;63(5-6):406–16. doi: 10.1002/mrc.5522 (PMC12053299; doi:10.1002/mrc.5522)
Supplement: Supplementary file 1 — Figure S1: 2H spectra measured during stepwise dilution of the samples to determine the critical concentration of DPPS‐PB(L/D)G in CDCl3 at 300 K and 107.5 MHz (700 MHz proton frequency). The full width at half maximum (FWHM, in Hz) of the right line of the doublet is given in gray. The values are determined using the TopSpin command peakw. Table S2: Composition of the anisotropic analyte samples (LLC phases) used for RDC analysis. Table S3: Additional spectra parameters that do not have a standard value. Figure S2: Superimposed (at the aromatic ring) structural models of the different conformers of galantamine. On the left side, conformers 1 and 2 are overlaid; on the right, conformers 3 and 4, and in the middle, all four conformers. The figure shows that conformers 1 and 2 differ only for the methoxy group (C18), irrelevant to RDC analysis, and can be treated as the same conformer. The same is the case for conformers 3 and 4. The middle part shows that conformers 1 (+2) and 3 (+4) differ in the conformation of the six‐ (C9‐C12) and sevenmembered (C13, C14, N15, C16) rings. Figure S3: 1H‐NMR (red, 600 MHz) and 13C‐NMR (blue, 151 MHz) of bromide 7 measured in CDCl3 at 300 K. Figure S4: 31P‐NMR (green, 243 MHz) of bromide 7 measured in CDCl3 at 300 K. Figure S5: ATR‐IR spectrum (neat) of bromide 7. Figure S6: HR‐MS (ESI positive) spectrum of bromide 7. The simulated/expected isotope pattern is compared with the experimental results. Figure S7: 1H‐NMR (red, 700 MHz) and 13C‐NMR (blue, 176 MHz) of ester (L)‐10 measured in DMSO‐d6 + DCl at 300 K. Figure S8: 31P‐NMR (green, 283 MHz) of ester (L)‐10 measured in DMSO‐d6 + DCl at 300 K. Figure S9: ATR‐IR spectrum (neat) of ester (L)‐10. Figure S10: HR‐MS (ESI positive) spectrum of ester (L)‐10. The simulated/expected isotope pattern is compared with the experimental results. Figure S11: 1H‐NMR (red, 600 MHz) and 13C‐NMR (blue, 151 MHz) of ester (D)‐10 measured in DMSO‐d6 + DCl at 300 K. For the 13C{1H} spectrum, an exponent [file MRC-63-406-s001.pdf]

Supporting Information

Synthesis and Application of a Hydrophobic Polyglutamate Bearing a Triphenylphosphine Group for the Orientation of Pharmaceutically Active Compounds and the Measurement of Residual Dipolar Couplings

Jan Rettig,[†] *Michael Gölz*[†] *and Christina M. Thiele**

Clemens-Schöpf-Institut für Organische Chemie und Biochemie, Technische Universität Darmstadt, Peter-Grünberg-Str. 16, Darmstadt D-64287, Germany

* E-mail: cthiele@thielelab.de

† Both authors contributed equally

Table of contents

1. List of abbreviations and symbols 4
2. Methods and General Procedures 6
   2.1 General remarks regarding the synthesis of the alignment medium 6
   2.2 Size exclusion chromatography (SEC) 7
   2.3 Circular dichroism spectroscopy (CD) 7
   2.4 NMR sample preparation 8
   2.5 NMR measurements for RDC analysis 10
   2.6 Generation of input structures/coordinates 12
3. Synthesis of the alignment medium 14
   3.1 Synthesis of 2-(4-bromophenyl)-1,3-dioxolane 14
   3.2 Synthesis of (4-(1,3-dioxolan-2-yl)phenyl)diphenylphosphine 15
   3.3 Synthesis of 4-(diphenylphosphanyl)benzaldehyde 16
   3.4 Synthesis of (4-(diphenylphosphanyl)phenyl)methanol 17
   3.5 Synthesis of (4-(hydroxymethyl)phenyl)diphenylphosphine sulfide 18
   3.6 Synthesis of (4-(bromomethyl)phenyl)diphenylphosphine sulfide 19
   3.7 Synthesis of (D)- and (L)-glutamic acid copper II complex 22
   3.8 Synthesis of (L)-2-amino-5-((4-(diphenylphosphorothioyl)benzyl)oxy)-5-oxopentanoic acid (DPPS-BLG ester) 23
   3.9 Synthesis of (D)-2-amino-5-((4-(diphenylphosphorothioyl)benzyl)oxy)-5-oxopentanoic acid (DPPS-BDG ester) 26
   3.10 Synthesis of 4-(diphenylphosphorothioyl)benzyl (L)-3-(2,5-dioxooxazolidin-4-yl)propanoate (DPPS-BLG-NCA) 29
   3.11 Synthesis of 4-(diphenylphosphorothioyl)benzyl (D)-3-(2,5-dioxooxazolidin-4-yl)propanoate (DPPS-BDG-NCA) 32
   3.12 Synthesis of the polymer DPPS-PBLG 35
   3.13 Synthesis of the polymer DPPS-PBDG 39
4. NMR (coupling) data of the analytes 43
   4.1 (-)-Isopinocampheol 43
   4.2 α-Santonin 47
   4.3 Artemisinin 50
   4.4 (+)-Vincamine 54
   4.5 (-)-Galantamine 58
5. Orientational properties of analytes (RDC@hotFCHT fits) 62
   5.1 (-)-Isopinocampheol 62
   5.2 α-Santonin 65
   5.3 Artemisinin 67
   5.4 (+)-Vincamine 68

5.5 (-)-Galantamine 69
6. Input structures/coordinates 72
6.1 α-santonin 72
6.2 (+)-Vincamine 73
6.3 (-)-Galantamine 74
7. CREST run for galantamine 79
8. Download NMR raw data 82
9. Author Contributions 83
10. Literature 84

1. List of abbreviations and symbols

abbreviation/ symbol	description
**#RDC**	number of RDCs used for analysis
**ACN**	acetonitrile
**ALPB**	analytical linearized Poisson-Boltzmann model
**APCI**	atmospheric pressure chemical ionization
**ATR-IR**	attenuated total reflection infrared (spectroscopy)
**BCU**	Bruker cooling unit
β	(5D) β-angle / °
c	concentration / mol·L^{-1}
**CD**	circular dichroism
**CLIP CLAP**	clean in-phase / clean anti-phase
**CN**	condition number
**COD**	crystallography open database
**CPCM**	conductor-like polarizable continuum model
**cpl**	coupled
**CREST**	conformer-rotamer ensemble sampling tool
**CSD**	Cambridge structural database
D	dipolar coupling / Hz
d	pathlength (cuvette) / mm
**PDI / Đ**	polydispersity index
$^{1}D_{CC}$	one-bond carbon-carbon RDC / Hz
$^{1}D_{CH}$	one-bond carbon-hydrogen RDC / Hz
$^{n}D_{CH}$	long-range carbon-hydrogen RDC / Hz
$^{n}D_{HH}$	long-range hydrogen-hydrogen RDC / Hz
D_a	axial component of the SAUPE tensor
D_{calc}	back-calculated RDC / Hz
D_{exp}	experimental RDC / Hz
ΔD	error of the RDC / Hz
ΔJ	error of the J-coupling / Hz
ΔT	error of the T-coupling / Hz
$\Delta\nu_Q$	quadrupolar splitting / Hz
**DCM**	dichloromethane
**DFT**	density functional theory
**DMEA**	*N*,*N*-dimethylethanolamine
**DMSO**	dimethylsulfoxide
**DPP-PB(L/D)G**	diphenyl phosphine-PB(L/D)G
**DPPS-PB(L/D)G**	diphenyl phosphine sulfide-PB(L/D)G
D_r	rhombic component of the SAUPE tensor
**EDTA**	ethylenediaminetetraacetate
**EI**	electron ionization
eq.	equivalent (synthesis)
E_{rel}	relative energy / kcal·mol^{-1}
**ESI**	electron spray ionization
**Euler α, β, γ**	Euler angles to describe the relative orientation of the principle axis frame of the order tensor to the molecule-fixed frame (ZY'Z'' convention) / °
F_1	indirect spectral dimension
F_2	direct spectral dimension
**FID**	free induction decay
**GDO**	generalized degree of order
γ	gyromagnetic ratio / rad·T^{-1}·s^{-1}
**HMPA**	hexamethylphosphoramide
**HR**	high-resolution
**IPC**	isopinocampheol
J	scalar coupling / Hz
**LLC**	lyotropic liquid crystal
M	molar mass / g·mol^{-1}
m	mass / g
**MALDI TOF**	matrix-assisted laser-desorption-ionization time of flight
**MCMT**	multi-conformer-multi-tensor
**MCST**	multi-conformer-single-tensor
$\overline{M_n}$	number average molecular weight
$\overline{M_w}$	weight average molecular weight
$\overline{M_p}$	molecular weight of the peak maximum
**MQEvo**	multiquantum evolution
**MS**	mass spectrometry
n	amount of substance / mol
**nOe**	nuclear Overhauser effect
**NCA**	*N*-carboxy anhydride
**PB(L/D)G**	poly-γ-L/D-glutamate
$\hbar$	reduced Planck constant / kg·m^{2}·s^{-1}
p_x	population of conformer x
**PSS**	polymer standards service (Mainz, Germany)
Q	Cornilescu quality factor
R	rhombicity of the Alignment tensor
**RCSA**	residual chemical shift anisotropy
**RDC**	residual dipolar coupling
**hotFCHT**	for hot bands, FC: Franck-Condon, HT: Herzberg-Teller
R_f	retention factor (TLC)
r	distance between spins / Å
**RMSD**	root-mean-square-deviation / Hz
**RQC**	residual quadrupolar coupling
**RT**	room / ambient temperature
**SCST**	single-conformer-single-tensor
**SDV**	styrene-divinylbenzene
**SEC**	size-exclusion chromatography
**SVD**	singular value decomposition
T	temperature / °C or K
**TBAB**	tetrabutylammonium bromide
**TFA**	trifluoroacetic acid

$\boldsymbol{\theta}$	angle between an inter-spin vector and the external magnetic field / °
**THF**	tetrahydrofuran
**TLC**	thin-layer chromatography
**TMG**	*N*,*N*,*N*',*N*'-tetramethylguanidine
**UV/Vis**	ultraviolet/visible light (spectroscopy)
$\boldsymbol{V}$	volume / mL
$\boldsymbol{\mu_0}$	vacuum permeability / N·A^{-2}
$\boldsymbol{w_p}$	weight percent / % (w/w)
$\boldsymbol{\xi_i}$	xi values to use for chemical shift referencing on the unified scale (IUPAC)

2. Methods and General Procedures

2.1 General remarks regarding the synthesis of the alignment medium

All used **chemicals** were bought from commercial sources (ABCR, Acros, Sigma Aldrich, etc.). If not described differently, the chemicals were used without any further purification. Reactions under inert conditions are performed with 99.999 % Argon from AIR LIQUIDE Deutschland GmbH. The silica used for column chromatography is Kieselgel 60 from MACHERY-NAGEL GmbH with particle diameters of 0.04 - 0.063 mm. Dry solvents were bought in the quality "Extra Dry" with an Acroseal® and are stored over 4 Å molecular sieves. All reactions using dry solvents were carried out under inert conditions in flame-dried glassware.

For the **assignment of the proton-, carbon- and phosphorus resonances** ^{1}H, ^{13}C{1H}, ^{31}P{1H}, ^{1}H,^{1}H-COSY, ^{1}H,^{13}C-HSQC, and ^{1}H,^{13}C-HMBC experiments (Standard Bruker pulse sequences) were measured at 300 K on either a NMR spectrometer (Bruker AVANCE III HD) with a ^{1}H frequency of 700 MHz equipped with a QCI cryoprobe (^{1}H/^{19}F-^{31}P/^{13}C/^{15}N/^{2}H) with z-gradient or a NMR spectrometer (Bruker AVANCE III) with a ^{1}H frequency of 600 MHz equipped with a triple resonance broadband inverse probe (^{1}H/^{31}P/^{2}H-BB) with z-gradient. The spectra were analyzed using *MestreNova* (MestreLab, version 14.2.1) or *TopSpin* (Bruker, version 3.5 pl. 7). The proton NMR spectra were referenced to the residual protonated solvent signal of $CDCl_3$ (δ_{1H} = 7.26 ppm), DMSO-d_6 (δ_{1H} = 2.50 ppm), or THF-d_8 (δ_{1H} = 1.72 ppm or 3.58 ppm).[1] All other spectra (^{13}C, ^{31}P, and 2D NMR experiments) were referenced relative to the proton spectra using the ξ values as recommended by IUPAC[2] (*xiref* command in TopSpin or the *Absolute Reference* tool in MestreNova). The ^{1}H spectra were processed using an exponential line broadening of 0.3 Hz and zero-filled by a factor of four, whereas 1 Hz line broadening and a zero-filling factor of two was used for the ^{13}C{^{1}H}- and ^{31}P{1H}-spectra, if not stated otherwise.**IR spectra** were acquired using an ALPHA platinum-ATR from Bruker on neat substances (monomer precursors) or in solution (polymers). The progress of the polymerization was monitored using IR spectroscopy. The polymerization was complete after 20 h when the two carbonyl bands of the *N*-carboxy anhydride at ~1850 cm^{-1} and ~1780 cm^{-1} had vanished entirely, and the carbonyl band of the amide backbone at around ~1650 cm^{-1} was observable instead.

Mass spectra were measured on a Finnigan MAT 95 (EI-MS), Bruker Daltonik Autoflex speed TOF/TOF (MALDI-MS), or a Bruker Daltonik Impact II (ESI-MS).

2.2 Size exclusion chromatography (SEC)

SEC measurements were performed on a system with three PSS (polymer standards service (Mainz, Germany)) SDV (styrene-divinylbenzene) columns (porosities of 10^7, 10^5, and 10^3 Å and particle sizes of 10 µm) and a PSS SDV pre-column (particle size 10 µm). Chloroform, with 0.3 % (w/w) tetra-*N*-butylammonium bromide (TBAB) and 0.1 % (V/V) xylene as the internal standard, is used as the mobile phase. The polymers are dissolved in the mobile phase overnight, resulting in 1 mg/mL concentrations. The flow rate was set to 0.25 mL/min at 25 °C. For each measurement, 75 µL of the polymer solution was injected, and a JASCO UV-2075 plus detector, operating at 258 nm, was used for detection. The molecular weights and their distribution are determined against polystyrene standards supplied from PPS. As the random-coil, globular polystyrene systems are not well-suited models for rigid-rod polypeptides, differences in e.g. hydrodynamic radii and shape factors are not accounted for, and no viscosity data to apply a universal calibration is available,[3,4] the polymer masses given below cannot be used as absolute polymer masses but may be used qualitatively to compare different polymer batches with each other.

2.3 Circular dichroism spectroscopy (CD)

CD spectra of the polymers were acquired on a JASCO J-1500 spectrometer (detectors PM-539 and PML-534). The polypeptides were dissolved in chloroform, resulting in a 2.5 mg/mL solution concentration. The solution was transferred into custom-made demountable quartz cuvettes[5] with a path length of $d \sim 0.01$ mm. Spectra were acquired at 23 °C (Peltier cell holder PTC-510) using five accumulations in the 300 to 200 nm range with a scanning speed of 50 nm/min, a data pitch of 1 nm, and a bandwidth of 2 nm. A background in neat chloroform was acquired the same way and subtracted from the sample spectra.

2.4 NMR sample preparation

The analyte (-)-isopinocampheol (IPC, CAS No. 25465-65-0) was purchased from Sigma Aldrich, (-)-α-santonin (CAS No. 481-06-1) from ABCR, artemisinin (CAS No. 63968-64-9), (+)-vincamine (CAS No. 1617-90-9) from TCI, and (-)-galantamine (CAS No. 357-70-0) from MedChemExpress. (-)-IPC was purified via sublimation, and the other analytes were used as received. $CDCl_3$ (≥ 99.8 % deuterated) was purchased from Sigma Aldrich in ampoules of 0.75 mL and used as received.

The isotropic samples were prepared by directly weighing the analyte into 5 mm standard quality NMR tubes. An appropriate amount of solvent (see **Table S1**) was added, and the tube was flame-sealed under a vacuum using an acetylene-oxygen burner. The sealed tube was shaken to ensure optimal homogeneity and dissolution of the analytes.

Table S1: Composition of the isotropic analyte samples used for RDC analysis.

**analyte A**	**M(A) /** g/mol	**m(A) /** mg	**n(A) /** mmol	**m($CDCl_3$)** / mg	**V($CDCl_3$)** / mL	**c(A) /** mmol/L
(-)-IPC	154.25	20.02	0.130	605.28	0.404	322
α-santonin	246.30	14.88	0.060	579.79	0.387	155
artemisinin	282.33	10.13	0.036	779.10	0.519	69
vincamine	354.44	10.23	0.029	778.31	0.519	56
galantamine	287.35	9.94	0.035	820.66	0.547	64

Before RDC analysis, the critical concentrations of the two polymers DPPS-PBLG (L-polymer for short, internal batch number MG04-223) and DPPS-PBDG (D-polymer for short, internal batch number MG04-235) in chloroform solution are determined. Therefore, the procedure described below is performed for each polymer without adding an analyte, starting with a concentration significantly above the concentration used for RDC analysis. After the homogenization of each sample, a ^2H spectrum is acquired. The sample is considered completely anisotropic if this spectrum shows the $CDCl_3$ resonance split into a doublet (by $\Delta\nu_Q$) with sharp, symmetric lines. Stepwise, more $CDCl_3$ is added, the sample is homogenized, and a ^2H spectrum is acquired. This is repeated until the ^2H spectrum shows a partially isotropic $CDCl_3$ signal or the doublet lines become broad and asymmetric ("tailing" into the direction of the respective other line, see **Figure S1**).

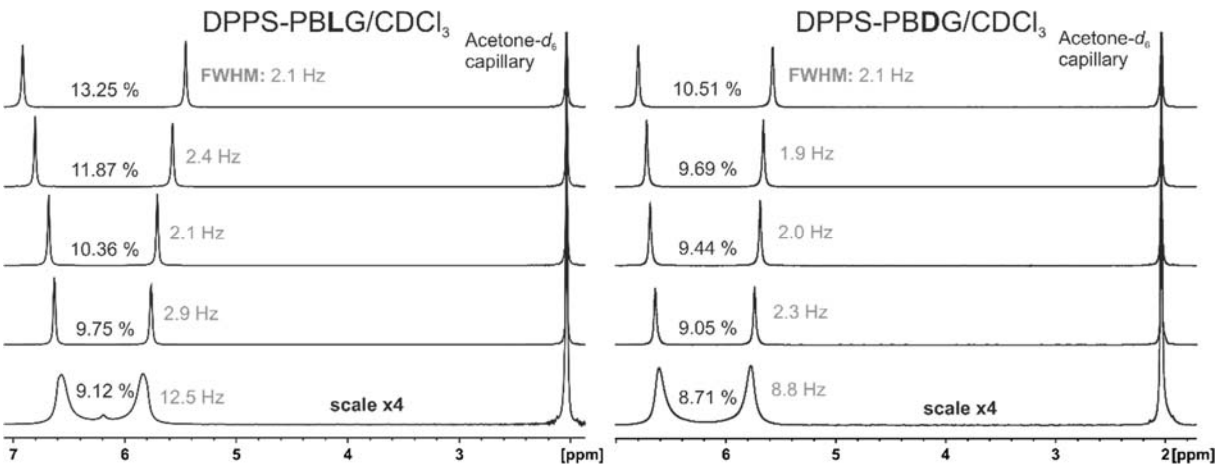

Figure S1: ^{2}H spectra measured during stepwise dilution of the samples to determine the critical concentration of DPPS-PB(L/D)G in $CDCl_3$ at 300 K and 107.5 MHz (700 MHz proton frequency). The full width at half maximum (FWHM, in Hz) of the right line of the doublet is given in gray. The values are determined using the TopSpin command `peakw`.

Both polymers show a similar critical concentration of about 9.0 - 9.5 % (w/w) in chloroform at 300 K. The bottom spectra (scaled by a factor of 4) of each series show the broad and asymmetric $CDCl_3$ lines mentioned before. This state is not considered fully anisotropic anymore. Note that the critical concentration depends on the sample temperature and is higher at temperatures above 300 K.

Anisotropic samples (see **Table S2**) were prepared by directly weighing the polymer (and the respective analyte) into 5 mm standard quality NMR tubes. A sealed capillary containing isotropic ACN-d_3 or acetone-d_6 was added to each tube to ensure convenient locking and shimming of the sample. The sample was capped using a rubber plug and centrifuged back and forth (from bottom to top and vice versa, ~1600 rpm) at least ten times to achieve optimal sample homogeneity. The progress was monitored occasionally by placing the samples between crossed polarization filters to check for a homogenous birefringence and by measurements of ^{2}H-image[6] spectra. The quadrupolar splitting of $CDCl_3$ in each sample was determined by ^{2}H spectra, where narrow and symmetric line shapes are used as an indicator of homogeneity. The mass fraction w (% w/w) of polymer in solution is determined using the equation $w_p = m_p * (m_p + m_A + m_{solvent})^{-1}$. The mass of the solvent is determined directly before RDC measurements. The anisotropic samples prepared are listed in **Table S2**.

Table S2: Composition of the anisotropic analyte samples (LLC phases) used for RDC analysis.

Analyte A	polymer	m(p) / mg	m(A) / mg	m($CDCl_3$) / mg	w_p/ % (w/w)	$\Delta\nu_Q$ @300 K / Hz
(-)-IPC	L	57.01	12.08	467.61	10.62	103
(-)-IPC	D	57.14	12.18	466.45	10.67	105
α-santonin	D	58.82	10.06	556.41	9.41	86
artemisinin	D	58.95	11.33	543.10	9.61	92
vincamine	D	58.72	9.50	527.06	9.86	98
galantamine	D	60.91	9.73	583.16	9.32	85

2.5 NMR measurements for RDC analysis

All RDC-related measurements were performed at 300 K on an NMR spectrometer (Bruker AVANCE III HD) with a ^{1}H frequency of 700 MHz equipped with a QCI cryoprobe ($^{1}H/^{19}F$-$^{31}P/^{13}C/^{15}N/^{2}H$) with z-gradient and a Bruker Cooling Unit II (BCU-II) using a gas flow of 700 l/h.

The assignment of resonances to the analytes was carried out in isotropic solution using ^{1}H, $^{13}C\{^{1}H\}$, $^{1}H,^{1}H$-COSY, $^{1}H,^{13}C$-HSQC, $^{1}H,^{13}C$-HMBC, and $^{1}H,^{1}H$-NOESY spectra (standard Bruker pulse sequences).

The scalar ($^{1}J_{CH}$) and total couplings ($^{1}T_{CH}$) necessary for the calculations of RDCs were extracted from *perfect* CLIP/CLAP HSQCs,[7] CLIP/CLAP HSQCs[8] (Bruker pulse sequence *hsqcetgpiajcsp.2*), F_1-cpl HSQCs[9] (Bruker pulse sequence *hsqcbietgpjcsp.2*) and F_1-cpl HSQCs with multi quantum evolution[10] (Bruker pulse sequence *hsqcbietgpjcmqsp*). The latter could only be successfully applied to the LLC phases containing (-)-IPC. For the determination of coupling constants and their respective errors, rows (for (*perfect*) CLIP/CLAP HSQCs) or a sum of columns (for F_1-cpl (MQEvo) HSQCs) were extracted from 2D spectra and analyzed according to the cross-fitting procedure described by Kummerlöwe et al.[11]

All INEPT delays were optimized for a coupling constant ($^{1}J_{CH}$ or $^{1}T_{CH}$) of 145 Hz (CNST2). Each spectrum was acquired with a spectral width of 14 ppm and an offset of 4.7 ppm in the direct dimension. Before the Fourier transformation, apodization using a squared sine bell function (SSB = 2) was performed.

The F_2-coupled (*perfect*) CLIP HSQC spectra were acquired using 4 scans per increment with 8192 points (FID-resolution of 2.4 Hz) in the direct and 256 (IPC, vincamine, α-santonin), 512 (galantamine), or 1024 (artemisinin) points in the indirect dimension. Zero-filling was applied to both dimensions, resulting in 16384 (direct) and 1024 points (indirect), respectively.
The F_1-coupled HSQC spectra (F_1-cpl) were acquired using 2 or 4 scans per increment with 2048 points in the direct and 512 points in the indirect dimension. *J*-scaling (CNST16) was applied in the indirect dimension using a value of 4 (for α-santonin), 8, or 10 (for IPC). Zero-filling was applied to both dimensions, resulting in 4096 (direct) and 2048 points (indirect), respectively.
The F_1-coupled HSQC spectra with multiple quantum evolutions (F_1-cpl MQEvo, for LLC phases with (-)-IPC only) were acquired using 4 scans per increment with 2048 points in the direct and 256 points in the indirect dimension. In the indirect dimension, *J*-scaling (CNST16) was applied using a value of 8. Zero-filling was applied to both dimensions, resulting in 4096 (direct) and 1024 points (indirect), respectively.
The individual parameters used for each experiment and analyte can be found in **Table S3**.

Table S3: Additional spectra parameters that do not have a standard value.

**Analyte A**	**polymer**	**experiment**	**NS**	**SW1 /** ppm	**O1P /** ppm	**FID res. (FX)** /Hz
(-)-IPC	L / D	*perf.* CLIP	4	60	45.5	2.39 (F2)
		F_1-cpl	2	65	45.5	44.71 (F1)
		F_1-cpl MQEvo	4	30	33.5	41.27 (F1)
α-santonin	D	CLIP CLAP	4	55	31.5	2.39
		F_1-cpl	4	65	31.5	44.71 (F1)
artemisinin	D	CLIP CLAP	4	45	30.0	2.39
		F_1-cpl	4	62	30.0	21.32 (F1)
(+)-vincamine	D	CLIP CLAP	4	55	32.5	2.39
		F_1-cpl	4	80	35.0	55.02 (F1)
(-)-galantamine	D	CLIP CLAP	4	64	59.0	2.39
		F_1-cpl	4	85	58.0	58.47 (F1)

RDCs are calculated from the total ($^1T_{CH}$) and scalar ($^1J_{CH}$) couplings using the equation $^1D_{CH} = (^1T_{CH} - {}^1J_{CH})/2$.[12] RDCs of methyl groups ($^1D_{CH3}$) are converted into the respective $^1D_{CC}$ between the carbon atom of the methyl group and the directly neighbored carbon atom

using the **equation S1** given by Verdier et al.[13] where γ_C ($67.2829\ 10^6\ \frac{\mathrm{rad}}{\mathrm{Ts}}$) and γ_H ($267.5222\ 10^6\ \frac{\mathrm{rad}}{\mathrm{Ts}}$) are the gyromagnetic ratios of carbon atoms C and protons H, and r_{CH} (1.091 Å) and r_{CC} (1.507 Å) are bond lengths.

$$^{1}D_{CC} = {}^{1}D_{CH_3}(-3\,\gamma_C/\gamma_H)(r_{CH}{}^{3}/r_{CC}{}^{3}) \quad \text{(S1)}$$

2.6 Generation of input structures/coordinates

General remarks: Some structures used for RDC analysis are taken from the literature (IPC[14]; artemisinin[15]). If structures are generated, a suitable starting structure is chosen (either a crystal structure or a force-field optimized structure) and optimized via DFT. For DFT structure optimizations, the program ORCA (version 5.0.3.)[16,17] is used with the hybrid functional B3LYP[18–20] and the Pople-type 6-311+G(d) basis set.[21,22] The charge-dependent dispersion correction D4[23] and the conductor-like Polarizable Continuum Model (CPCM)[24] for chloroform are used. In the case of a more flexible compound, a conformational search is performed. Therefore, the software CREST (Conformer-Rotamer Ensemble Sampling Tool, version 2.12)[25–27] is used with the default settings (energy threshold 6 kcal/mol) and the analytical linearized Poisson-Boltzmann (ALPB)[28] implicit solvent model for chloroform.

The **α-santonin** structure was generated using the software Chem3D Ultra, version 19.1.0.8 (PerkinElmer Informatics). The structure is optimized using the built-in force field MMFF94[29] and DFT optimized as described above.

The **(+)-vincamine** structure is generated using the crystal structure (Crystallography Open Database (COD) entry 2311256)[30] as a starting point and DFT optimized as described above.

The **(-)-galantamine** structure was generated using the crystal structure (Cambridge Structural Database (CSD) Identifier SIBHAM, deposition number 1258428)[31,32] as a starting point. This structure is DFT optimized, as described above. A conformational search is conducted using CREST[25–27] (version 2.12), resulting in four conformers of reasonable energies and Boltzmann populations >1 % (see **SI section 7** and **Table 1**). Since conformers 1 and 2 and also 3 and 4 show identical conformations of the core moieties of the compound relevant for RDC analysis diverging only in the conformation of the exocyclic methoxy group (see **Figure S2**), only

conformers 1 and 3 are used for RDC analysis. The structures of the conformers are DFT optimized, as described above.

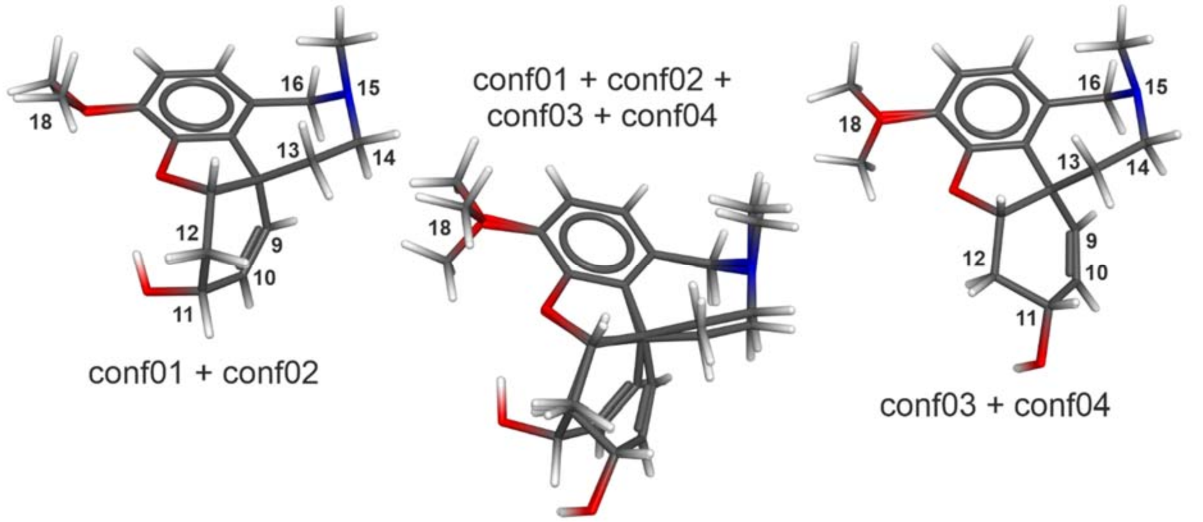

Figure S2: Superimposed (at the aromatic ring) structural models of the different conformers of galantamine. On the left side, conformers 1 and 2 are overlaid; on the right, conformers 3 and 4, and in the middle, all four conformers. The figure shows that conformers 1 and 2 differ only for the methoxy group (C18), irrelevant to RDC analysis, and can be treated as the same conformer. The same is the case for conformers 3 and 4. The middle part shows that conformers 1 (+2) and 3 (+4) differ in the conformation of the six- (C9-C12) and seven-membered (C13, C14, N15, C16) rings.

3. Synthesis of the alignment medium

Details, procedures, and analytical data regarding every reaction step can be found in this chapter.

3.1 Synthesis of 2-(4-bromophenyl)-1,3-dioxolane

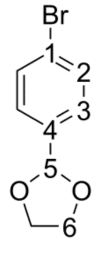

The synthesis is carried out according to a literature procedure.[33–35]

51.3 g 4-bromobenzaldehyde (277 mmol, 1 eq.) and 23.2 mL of ethylene glycol (412 mmol, 1.5 eq.) are dissolved in 1.5 L of toluene. 1.61 g *p*-toluenesulfonic acid monohydrate (8.5 mmol, 0.05 eq.) is added, and the reaction mixture is heated to 111 °C for 68 h using a Dean-Stark apparatus. After complete conversion, the organic layer was extracted with sat. aq. $NaHCO_3$ solution and sat. aq. NaCl solution. It was dried over $MgSO_4$, and the solvent was removed under reduced pressure. The crude product was purified by distillation at 2 mbar and 110 °C. 62.6 g (273 mmol, quant. yield) of a colorless liquid are obtained.

Internal batch number **MG04-22**
***R*f** (*n*-hexane/ethyl acetate 2:1) = 0.60
^{1}H-NMR (600 MHz, $CDCl_3$, 300 K): δ = 7.51 (d, $^3J_{HH}$ = 8.5 Hz, 2H_2), 7.35 (d, $^3J_{HH}$ = 8.5 Hz, 2H_3), 5.77 (s, 1H_5), 4.07 (m, 4H_6) ppm.
^{13}C{^{1}H}-NMR (151 MHz, $CDCl_3$, 300 K): δ = 137.94 (C_1), 131.81 (C_2), 128.72 (C_3), 123.40 (C_4), 103.38 (C_5), 64.78 (C_6) ppm.
ATR-IR (neat): $\tilde{\nu}$ = 3064 (=C-H), 1649 (arom. ring), 1594 (arom. ring), 1483 (arom. ring), 1295 (-C-O), 1217 (-C-O), 1065 (-C-Br), 811 (1,4-disubs. arom. ring) cm^{-1}.
MS (EI): Expected: 227 m/z, found: 227 m/z. Fragment: 183 m/z Br-Ar-CO^+.
The analytic data is consistent with literature data.[34]

3.2 Synthesis of (4-(1,3-dioxolan-2-yl)phenyl)diphenylphosphine

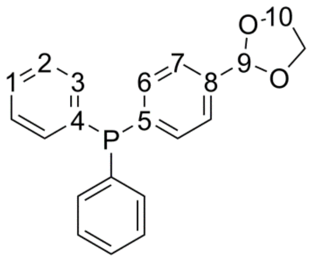

The synthesis is carried out according to a literature procedure.[33–35]

62.6 g 2-(4-bromophenyl)-1,3-dioxolane (271 mmol, 1 eq.) are dissolved in 850 mL of anhydrous THF. The solution is cooled down to -78 °C using an acetone/liquid nitrogen bath, and 130 mL of *n*-BuLi (2.5 mol/L in *n*-hexane, 325 mmol, 1.2 eq.) are added. The reaction mixture is stirred for 1 h, and 78.4 mL of chlorodiphenylphosphine (352 mmol, 1.3 eq.) is added. The reaction mixture is allowed to warm up slowly to ambient temperature overnight. The mixture is extracted with sat. aq. NH_4Cl solution. After removing the solvent under reduced pressure, the crude product was crystallized from MeOH. 68.2 g of colorless crystals (204 mmol, 75 % yield) are obtained.

Internal batch number **MG04-25**

***R*f** (*n*-hexane:CH_2Cl_2 1:1) = 0.16

^{1}H-NMR (600 MHz, $CDCl_3$, 300 K): δ = 7.45 (dd, ^3J = 7.0 Hz, ^4J = 1.0 Hz, $2H_7$), 7.35- 7.29 (m, $12H_{1,2,3,6}$), 5.81 (s, $1H_9$), 4.08 (m, $4H_{10}$) ppm.

^{13}C{^{1}H}-NMR (151 MHz, $CDCl_3$, 300 K): δ = 138.7 (d, $^1J_{PC}$ = 11 Hz, C_5), 138.5 (C_8), 137.1 (d, $^1J_{PC}$ = 10 Hz C_4), 133.9 ($C_{2/3}$), 133.8 ($C_{2/3}$), 128.9 (C_1), 128.7 (d, $^2J_{PC}$ = 7 Hz, C_6), 126.7 (d, $^3J_{PC}$ = 7 Hz, C_7), 103.6 (C_9), 65.5 (C_{10}) ppm.

^{31}P{^{1}H}-NMR (243 MHz, $CDCl_3$, 300 K): δ = -5.60 ppm.

ATR-IR (neat): $\tilde{\nu}$ = 3057 (=C-H), 1591 (arom. ring), 1558 (arom. ring), 1477 (arom. ring), 1453 (=C-P), 1261 (-C-O), 1205 (-C-O), 999 (=C-P), 820 (1,4-disubs. arom. ring), 743 (monosubs. benzene), 694 (monosubs. benzene) cm^{-1}.

MS (APCI): $C_{21}H_{20}O_2P$ $[M+H]^+$ requires 335 m/z, found 335 m/z.

The analytic data is consistent with literature data.[34]

3.3 Synthesis of 4-(diphenylphosphanyl)benzaldehyde

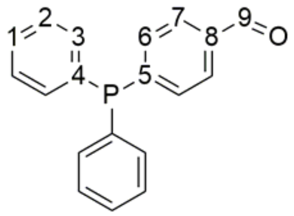

The synthesis is carried out according to a literature procedure.[34,35]

68.2 g (4-(1,3-dioxolan-2-yl)phenyl)diphenylphosphine (204 mmol, 1 eq.) are suspended in THF/water (500 mL:250 mL), and 1.19 g *p*-toluenesulfonic acid (6 mmol, 0.03 eq.) are added. The reaction mixture is heated to reflux for 20 h. The reaction mixture is allowed to cool down and is extracted with sat. aq. $NaHCO_3$ and sat. aq. NaCl solution. The solvent is removed under reduced pressure, and the crude product is recrystallized from MeOH. 49.7 g (171 mmol, 84 % yield) of colorless crystals are obtained.

Internal batch number **MG04-31**
***R*f** (*n*-hexane:ethyl acetate) = 0.25
^{1}H-NMR (600 MHz, $CDCl_3$, 300 K): δ = 10.01 (s, $1H_9$), 7.81 (d, $^{3}J_{HH}$ = 7.5 Hz, $2H_7$), 7.42 (pseudo t, J = 7 Hz, $2H_6$), 7.38 (m, $4H_2$), 7.35 (m, $4H_3$), 7.40 (m, $2H_1$) ppm.
^{13}C{^{1}H}-NMR (151 MHz, $CDCl_3$, 300 K): δ = 192.05 (C_9), 146.65 (d, $^{1}J_{PC}$ = 16 Hz, C_5) 136.16 (C_8), 135.94 (d, $^{1}J_{PC}$ = 11 Hz, C_4), 134.19 (d, $^{2}J_{PC}$ = 20 Hz, C_3), 133.67 (d, $^{2}J_{PC}$ = 19 Hz, C_6), 129.46 (C_1), 129.44 (C_7), 128.91 (d, $^{3}J_{PC}$ = 7 Hz, C_2) ppm.
^{31}P{^{1}H}-NMR (243 MHz, $CDCl_3$, 300 K): δ = -4.27 ppm.
ATR-IR (neat): $\tilde{\nu}$ = 3052 (=C-H), 2820 (-OC-H), 1696 (C=O), 1590 (arom. ring), 1556 (arom. ring), 1476 (arom. ring), 1431 (=C-P), 999 (=C-P), 823 (1,4-disubs. arom. ring), 744 (monosubs. benzene), 691 (monosubs. benzene) cm^{-1}.
MS (EI): Expected: 290 m/z, found: 290 m/z. Fragment: 183 m/z Br-Ar-CO^{+}.
The analytic data is consistent with the literature data.[34]

3.4 Synthesis of (4-(diphenylphosphanyl)phenyl)methanol

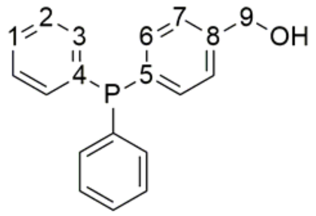

The synthesis is carried out according to a literature procedure.[36]

49.7 g 4-(Diphenylphosphino)benzaldehyde (171 mmol, 1 eq.) are suspended in 1 L anhydrous MeOH. 5.63 g $NaBH_4$ (149 mmol, 0.87 eq.) are added in small portions. The reaction mixture is stirred for 2 h and quenched by adding water. The organic and the aqueous layers are separated, and the volume of the organic layer is reduced *in vacuo*. It is extracted with sat. aq. $NaHCO_3$ and sat. aq. NaCl solution. The solvent is removed under reduced pressure. 48.6 g (166 mmol, 97 % yield) of a colorless oil are obtained.

Internal batch numbers **MG03-7**, **MG04-32**
$\boldsymbol{R_f}$ (*n*-hexane:ethyl acetate 1:1) = 0.25
^{1}H-NMR (600 MHz, $CDCl_3$, 300 K): δ = 7.70 - 7.32 (m, $14H_{1,2,3,6,7}$), 4.72 (s, $2H_9$) ppm.
^{13}C{^{1}H}-NMR (151 MHz, $CDCl_3$, 300 K): δ = 141.6 (C_8), 137.0 (d, $^1J_{PC}$ = 10 Hz, C_4), 136.4 (d, $^1J_{PC}$ = 10 Hz, C_5), 134.1 (d, J_{PC}= 20 Hz, $C_{1/2/3/6/7}$), 133.8 (d, J_{PC}= 18 Hz, $C_{1/2/3/6/7}$), 129.1 (s, $C_{1/2/3/6/7}$), 128.7 (d, J_{PC} = 7 Hz, $C_{1/2/3/6/7}$), 127.2 (d, J_{PC} = 7 Hz, $C_{1/2/3/6/7}$), 65.0 (C_9) ppm.
^{31}P{^{1}H}-NMR (243 MHz, $CDCl_3$, 300 K): δ = -5.91 ppm.
ATR-IR (neat): $\tilde{\nu}$ = 3313 (-O-H), 3054 (=C-H), 2864 (-C-H), 1598 (arom. ring), 1583 (arom. ring), 1494 (arom. ring), 1434 (=C-P), 1399 (-O-H), 1038 (-C-O), 998 (=C-P), 803 (1,4-disubs. arom. ring), 742 (monosubs. benzene), 695 (monosubs. benzene) cm^{-1}.
MS (EI): Expected: 292 m/z, found: 292 m/z. Fragment: 183 m/z Br-Ar-CO^+.
The analytic data is consistent with the literature data.[37]

3.5 Synthesis of (4-(hydroxymethyl)phenyl)diphenylphosphine sulfide

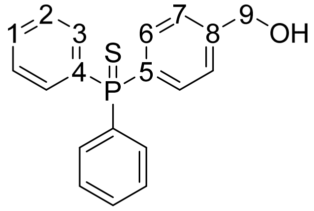

The synthesis is carried out according to a literature procedure.[38,39]

14.8 g (4-(Diphenylphosphino)phenyl)methanol (50.5 mmol, 1 eq.) is dissolved in 600 mL toluene, and 1.62 g sulfur (50.5 mmol, 1 eq.) is added. The reaction mixture is heated to reflux for 10 minutes. The solvent is removed under reduced pressure, and 16.4 g (50.5 mmol, quant. yield) of a colorless solid is obtained.

Internal batch number **MG04-33**
***R*f** (CH_2Cl_2) = 0.37
^{1}H-NMR (600 MHz, $CDCl_3$, 300 K): δ = 7.75-7.66 (m, 6$H_{3,6}$), 7.51 (m, 2H_1), 7.47-7.39 (m, 6$H_{7,2}$), 4.74 (s, 2H_9) ppm.
^{13}C{^{1}H}-NMR (151 MHz, $CDCl_3$, 300 K): δ = 144.8 (d, $^4J_{PC}$ = 3 Hz, C_8), 133.0 (d, $^1J_{PC}$ = 85 Hz, C_4), 132.7 (d, $^2J_{PC}$ = 11 Hz, C_6), 132.4 (d, $^2J_{PC}$ = 11 Hz, C_3), ~132.0 (d, $^1J_{PC}$ = ~85 Hz C_5, no exact shift and coupling due to signal overlap), 131.7 (d, $^4J_{PC}$ = 3 Hz, C_1), 128.7 (d, $^3J_{PC}$ = 13 Hz, C_2), 126.8 (d, $^3J_{PC}$ = 13 Hz, C_7), 64.6 (C_9) ppm.
^{31}P{^{1}H}-NMR (243 MHz, $CDCl_3$, 300 K): δ = 43.0 ppm.
ATR-IR (neat): $\tilde{\nu}$ = 3395 (-O-H), 3051 (=C-H), 2864 (-C-H), 1602 (arom. ring), 1585 (arom. ring), 1496 (arom. ring), 1434 (=C-P), 1396 (-O-H), 1102 (-C-O), 997 (=C-P), 808 (1,4-disubs. arom. ring), 750 (monosubs. benzene), 691 (monosubs. benzene), 638 (P=S) cm^{-1}.
MS (ESI positive): $C_{19}H_{18}OPS$ $[M+H]^+$ requires 325 m/z, found 325 m/z.
The analytic data is consistent with literature data with small differences in the ^{1}H and ^{13}C NMR chemical shifts from those reported.[40]

3.6 Synthesis of (4-(bromomethyl)phenyl)diphenylphosphine sulfide

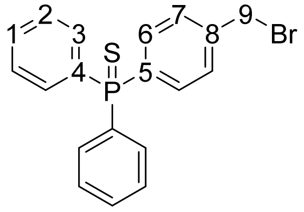

The synthesis is carried out according to a literature procedure.[41]

10 g (4-(hydroxymethyl)phenyl)diphenylphosphine sulfide (30.8 mmol, 1 eq.) is dissolved in 300 mL anhydrous DCM and cooled down to 0 °C. 2.95 mL thionyl bromide (37 mmol, 1.2 eq.) is added slowly. The reaction is stirred at room temperature for 2 h and quenched by adding 50 mL water. The organic layer is washed with water (100 mL) twice and dried over $MgSO_4$. The solvent is removed under reduced pressure. The crude product is purified by column chromatography (*n*-hexane:CH_2Cl_2 2:3). 4.6 g of a colorless solid (11.9 mmol, 39 % yield) is obtained.

Internal batch numbers **MG03-31** and **MG04-36**

$\boldsymbol{R_f}$ (*n*-hexane:CH_2Cl_2 2:3) = 0.49

^{1}H-NMR (600 MHz, $CDCl_3$, 300 K): δ = 7.76-7.66 (m, 6$H_{3,6}$), 7.52 (m, 2H_1), 7.49-7.42 (m, 6$H_{2,7}$), 4.49 (s, 2H_9) ppm.

^{13}C{^{1}H}-NMR (151 MHz, $CDCl_3$, 300 K): δ = 141.4 (d, $^4J_{PC}$ = 3 Hz, C_8), 133.4 (d, $^1J_{PC}$ = 85 Hz, C_5), 132.9 (d, $^2J_{PC}$ = 11 Hz, C_6), 132.8 (d, $^1J_{PC}$ = 85 Hz, C_4), 132.4 (d, $^2J_{PC}$ = 11 Hz, C_3), 131.8 (d, $^4J_{PC}$ = 3 Hz, C_1), 129.2 (d, $^3J_{PC}$ = 13 Hz, C_7), 128.7 (d, $^3J_{PC}$ = 13 Hz, C_2), 32.2 (d, $^5J_{PC}$ = 1.5 Hz, C_9) ppm.

^{31}P{^{1}H}-NMR (243 MHz, $CDCl_3$, 300 K): δ = 42.9 ppm.

ATR-IR (neat): $\tilde{\nu}$ = 3049 (=C-H), 2956 (-C-H), 1598 (arom. ring), 1585 (arom. ring), 1497 (arom. ring), 1434 (=C-P), 999 (=C-P), 816 (1,4-disubs. arom. ring), 750 (monosubs. benzene), 689 (monosubs. benzene), 670 (-C-Br), 636 (P=S) cm^{-1}.

MS (HR-ESI positive): $C_{19}H_{17}BrPS$ $[M+2+H]^+$ requires 388,99476 m/z, found 388,99469 m/z (Δ = 0.18 ppm).

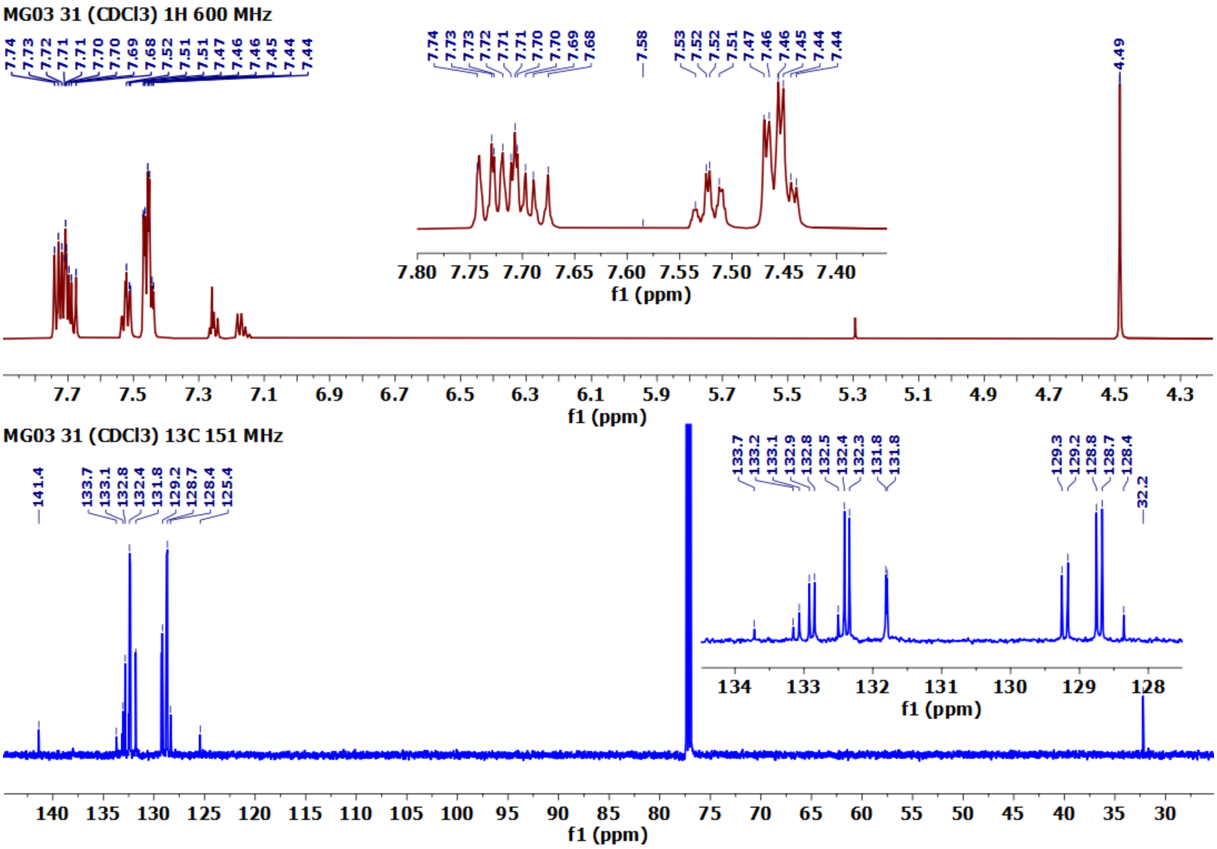

Figure S3: ^{1}H-NMR (red, 600 MHz) and ^{13}C-NMR (blue, 151 MHz) of bromide **7** measured in $CDCl_3$ at 300 K.

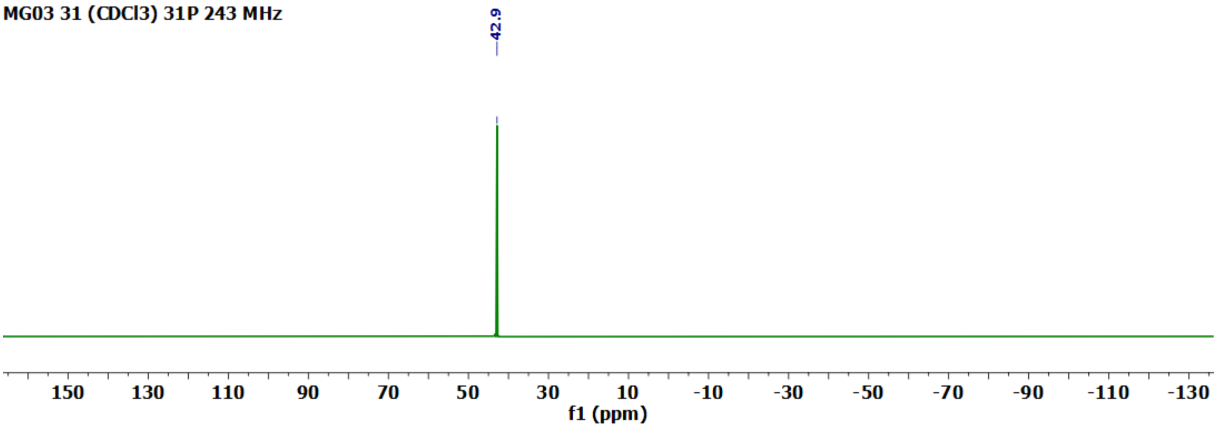

Figure S4: ^{31}P-NMR (green, 243 MHz) of bromide **7** measured in $CDCl_3$ at 300 K.

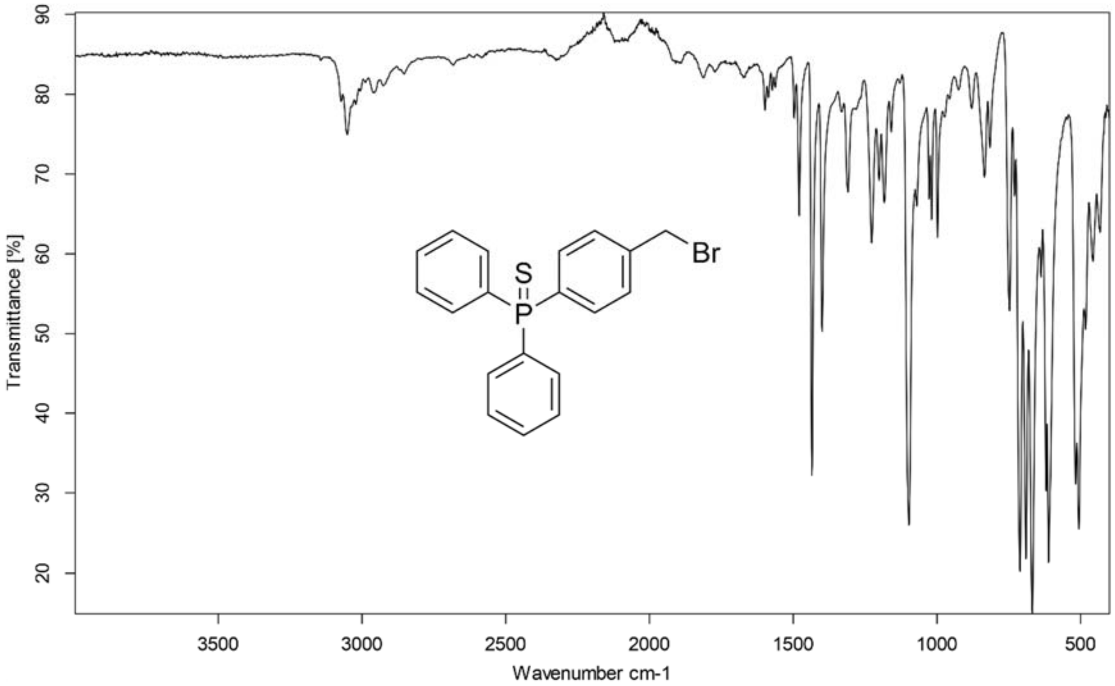

Figure S5: ATR-IR spectrum (neat) of bromide **7**.

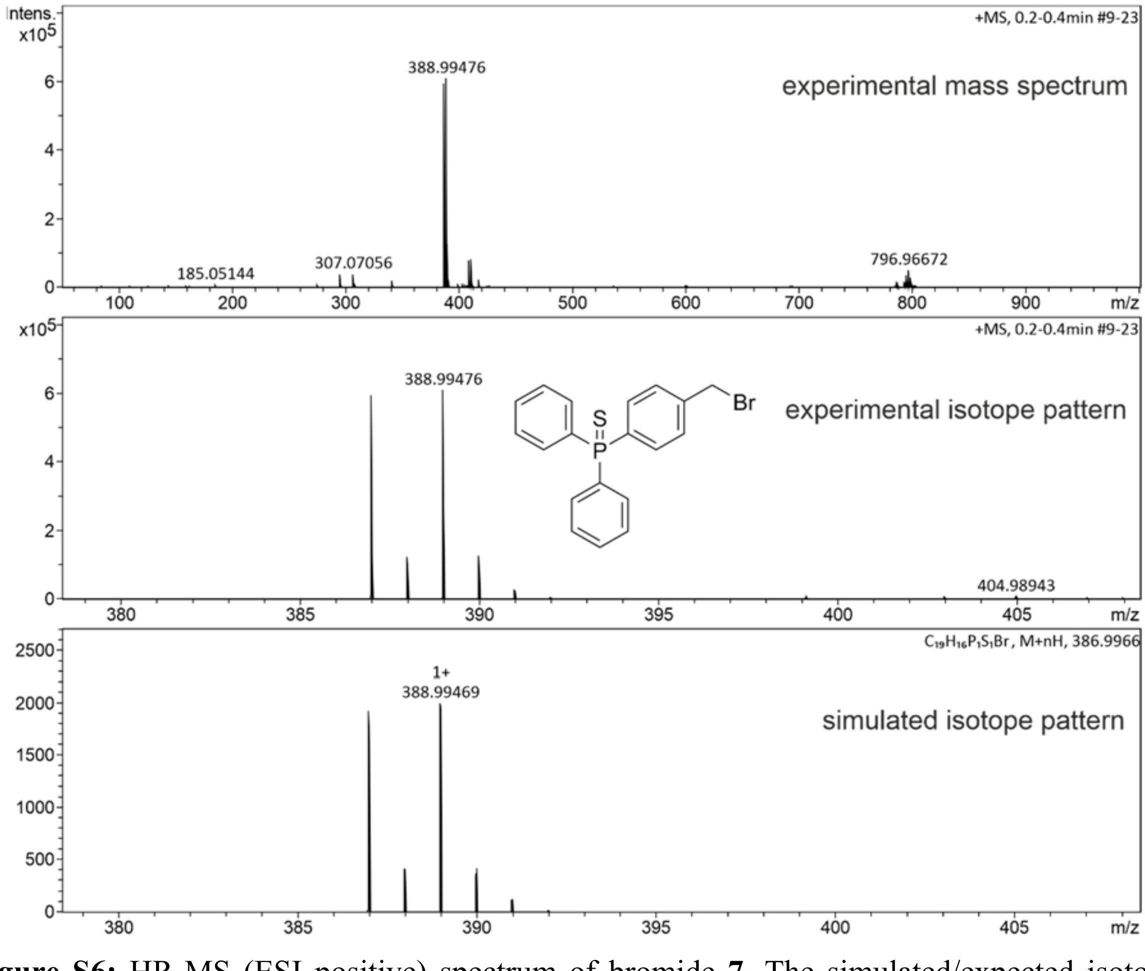

Figure S6: HR-MS (ESI positive) spectrum of bromide **7**. The simulated/expected isotope pattern is compared with the experimental results.

3.7 Synthesis of (D)- and (L)-glutamic acid copper II complex

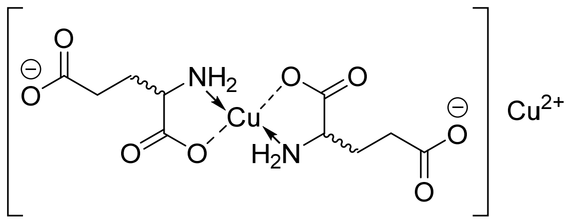

The following procedure (from literature[42]) was used to synthesize the (D)-glutamic acid copper II complex and (L)-glutamic acid copper II complex from the respective enantiomer of glutamic acid.

30 g (D)- or (L)-glutamic acid (204 mmol, 1 eq.) are added to 750 mL of water. After heating to 70 °C, a copper (II) acetate solution (42.7 g, 214 mmol, 1.05 eq. in 750 mL water) is added slowly. The reaction mixture is stirred for 2 days at ambient temperature, and the precipitated product is isolated by filtration and washed with water, EtOH, and diethyl ether. The residue is dried in a high vacuum. 45.6 g ((D), 91% yield) and 45.4 g ((L), 91 % yield) of a blue solid are obtained.

Internal batch numbers **MG04-136** for (D) and **MG03-33** for (L).
Due to the poor solubility of the complexes, no analytic data is acquired, and the crude products are used without further purification in the subsequent steps.

3.8 Synthesis of (L)-2-amino-5-((4-(diphenylphosphorothioyl)benzyl)oxy)-5-oxopentanoic acid (DPPS-BLG ester)

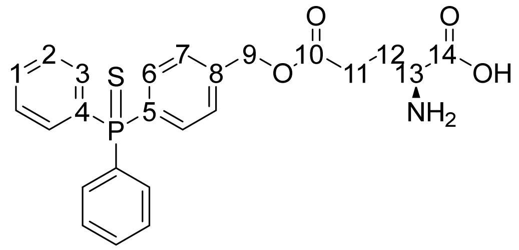

The synthesis is carried out according to a literature procedure.[42,43]

847 mg (L)-glutamic acid (5.7 mmol, 0.48 eq.) and 1.63 g (L)-glutamic acid copper II complex (3.3 mmol, 2.8 eq.) are added to a mixture of 5.3 mL DMF and 0.9 mL water. 1.6 mL *N*,*N*,*N*',*N*'-tetramethylguanidine (12.5 mmol, 1.05 eq.) is added slowly, and the reaction mixture is stirred for 2 h at ambient temperature. A solution of 4.60 g (4-(bromomethyl)phenyl)-diphenylphosphine sulfide (11.9 mmol, 1 eq.) in 10 mL DMF is added. The reaction is stirred for 20 h, and acetone is added to precipitate the product. The precipitate is filtered off and washed with acetone. The blue residue is stirred in a freshly prepared Na_2-EDTA solution (20 g EDTA (5.7 eq.), 11.6 g $NaHCO_3$, 170 mL water) for 2 h. The near-colorless solid is filtered off and washed with water. Residual solvents were removed in high vacuum to obtain 3.23 g (7.1 mmol, 60 % yield) of a near-colorless, slightly blue solid.

Internal batch numbers **MG04-37** and **MG04-173**
^{1}H-NMR (700 MHz, DMSO-d_6+DCl, 300 K): δ = 7.69-7.49 (m, 14$H_{1,2,3,6,7}$), 5.14 (s, 2H_9), 3.89 (pseudo-t, $^3J_{HH}$ = 6.4 Hz, 1H_{13}), 2.72-2.48 (m, 2H_{11}), 2.13-1.99 (m, 2H_{12}) ppm.
^{13}C{^{1}H}-NMR (176 MHz, DMSO-d_6+DCl, 300 K): δ= 171.9 (s, C_{10}), 170.4 (s, C_{14}), 140.6 (d, $^4J_{PC}$ = 3 Hz, C_8),132.8- 131.2 ($C_{1,4,5,6/7}$), 132.1 (d, J_{PC} = 11 Hz, $C_{2/3}$), 129.3 (d, J_{PC} = 12 Hz, $C_{2/3}$), 128.2 (d, J_{PC} = 12 Hz, $C_{6/7}$), 65.2 (s, C_9), 51.3 (s, C_{13}), 29.5 (s, C_{11}), 25.2 (s, C_{12}) ppm.
^{31}P{^{1}H}-NMR (283 MHz, DMSO-d_6+DCl, 300 K): δ = 41.9 ppm.
ATR-IR (neat): $\tilde{\nu}$ = 3054 (=C-H), 2934 (-C-H), 1727 (C=O), 1608 (arom. ring), 1431 (=C-P), 1178 (-C-O), 1096 (-C-O), 713 (monosubs. benzene), 693 (monosubs. benzene), 513 (P=S) cm^{-1}.
MS (HR-ESI positive): $C_{24}H_{25}NO_4PS$ $[M+H]^+$ requires 454.12364 m/z, found 454.12301 m/z (Δ = 1.39 ppm).

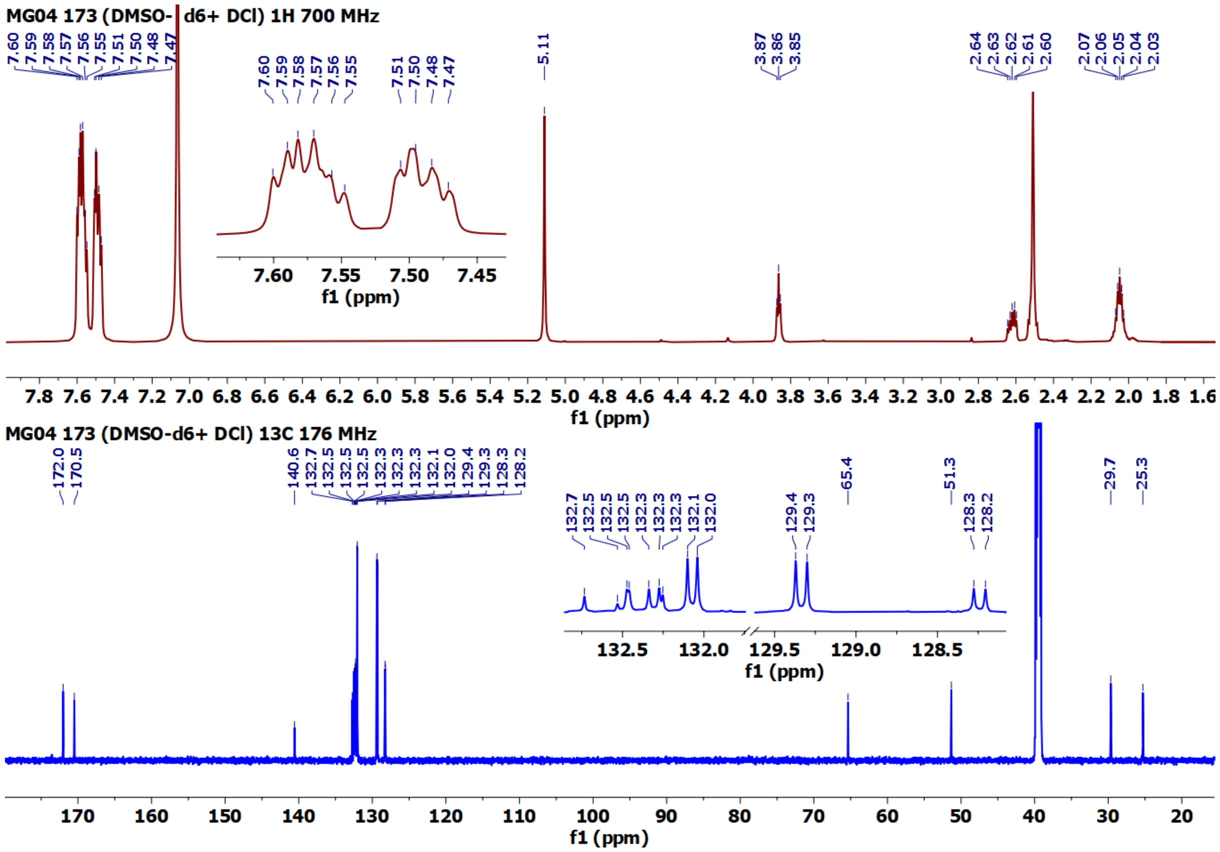

Figure S7: ^{1}H-NMR (red, 700 MHz) and ^{13}C-NMR (blue, 176 MHz) of ester (L)-**10** measured in DMSO-d_6 + DCl at 300 K.

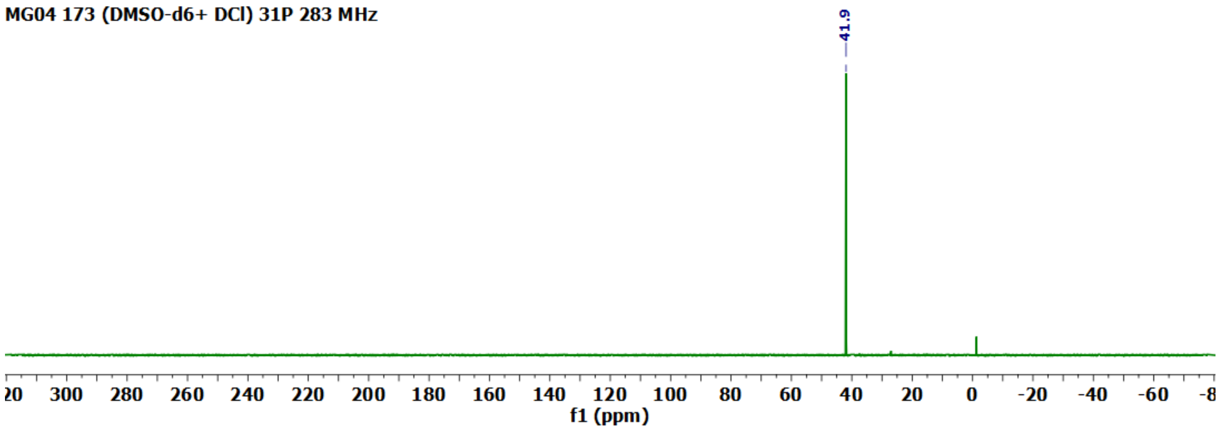

Figure S8: ^{31}P-NMR (green, 283 MHz) of ester (L)-**10** measured in DMSO-d_6 + DCl at 300 K.

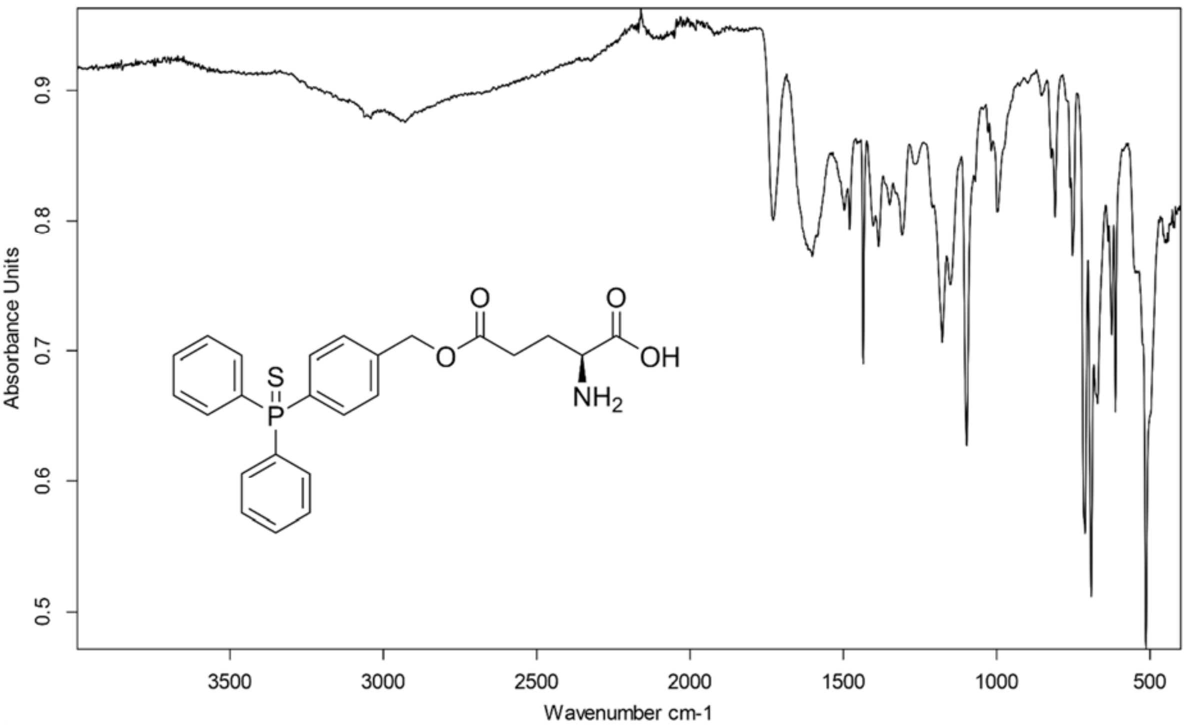

Figure S9: ATR-IR spectrum (neat) of ester (L)-**10**.

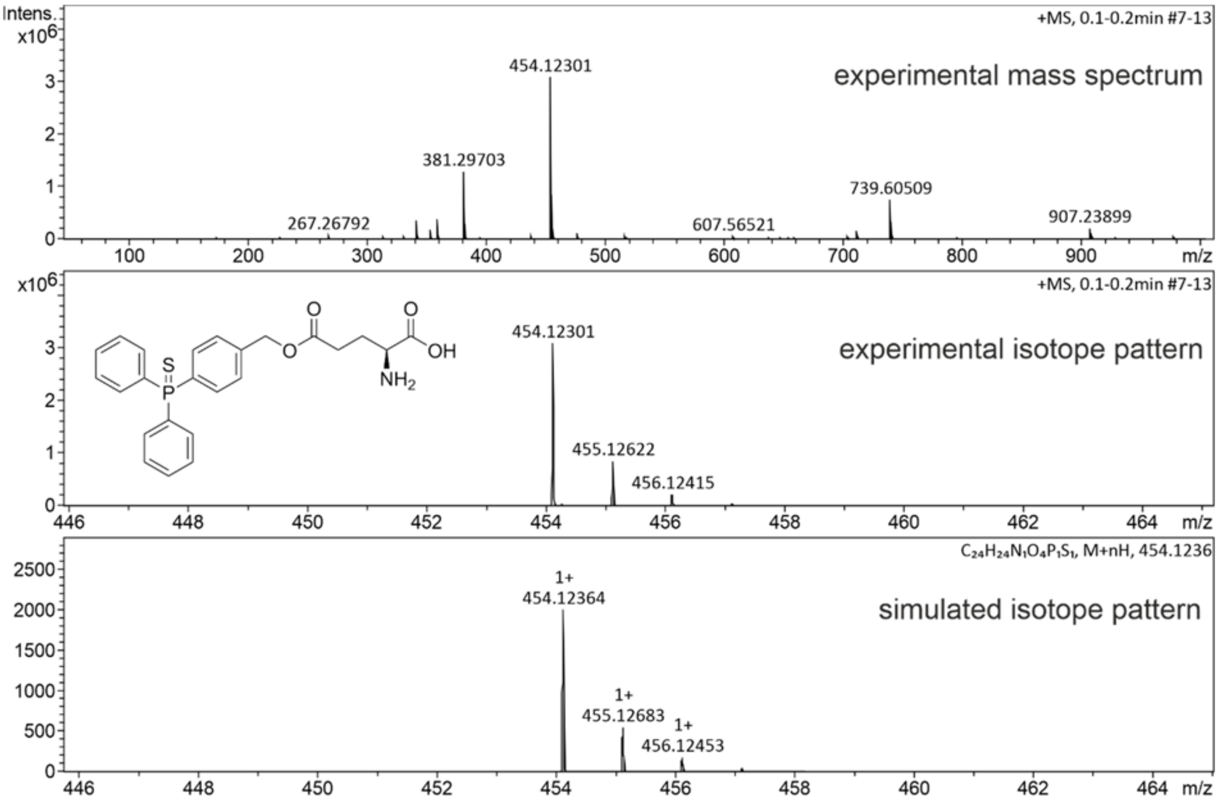

Figure S10: HR-MS (ESI positive) spectrum of ester (L)-**10**. The simulated/expected isotope pattern is compared with the experimental results.

3.9 Synthesis of (D)-2-amino-5-((4-(diphenylphosphorothioyl)benzyl)oxy)-5-oxopentanoic acid (DPPS-BDG ester)

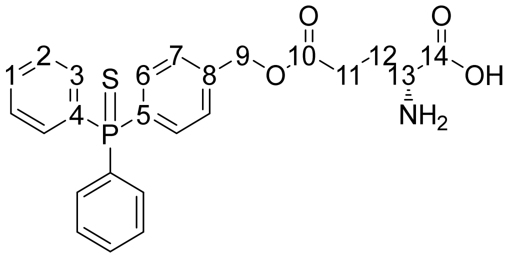

The synthesis is carried out according to a literature procedure.[42,43]

830 mg (D)-glutamic acid (5.6 mmol, 0.48 eq.) and 1.61 g (D)-glutamic acid copper II complex (3.3 mmol, 2.8 eq.) are added to a mixture of 5.3 mL DMF and 0.9 mL water. 1.6 mL *N,N,N',N'*-tetramethylguanidine (12.5 mmol, 1.05 eq.) is added slowly, and the reaction mixture is stirred for 2 h at ambient temperature. A solution of 4.61 g (4-(bromomethyl)phenyl)-diphenylphosphine sulfide (11.9 mmol, 1 eq.) in 10 mL DMF is added. The reaction is stirred for 20 h, and acetone is added to precipitate the product. The precipitate is filtered off and washed with acetone. The blue residue is stirred in a freshly prepared Na_2-EDTA solution (20 g EDTA (5.7 eq.), 11.6 g $NaHCO_3$, 170 mL water) for 2 h. The near-colorless solid is filtered off and washed with water. Residual solvents were removed in high vacuum to obtain 2.94 g (6.5 mmol, 55 % yield) of a near-colorless, slightly blue solid.

Internal batch numbers **MG04-233** and **MG04-279**

^{1}H-NMR (700 MHz, DMSO-d_6+DCl, 300 K): δ = 7.62-7.49 (m, 14$H_{1,2,3,6,7}$), 5.13 (s, 2H_9), 3.88 (pseudo-t, $^3J_{HH}$ = 6.4 Hz, 1H_{13}), 2.67-2.53 (m, 2H_{11}), 2.08-2.04 (m, 2H_{12}) ppm.

^{13}C{^{1}H}-NMR (176 MHz, DMSO-d_6+DCl, 300 K): δ= 172.0 (s, C_{10}), 170.5 (s, C_{14}), 140.6 (d, $^4J_{PC}$ = 3 Hz, C_8),132.8- 132.3 ($C_{1,4,5,6/7}$), 132.1 (d, J_{PC} = 11 Hz, $C_{2/3}$), 129.3 (d, J_{PC} = 12 Hz, $C_{2/3}$), 128.2 (d, J_{PC} = 12 Hz, $C_{6/7}$), 65.3 (s, C_9), 51.3 (s, C_{13}), 29.7 (s, C_{11}), 25.3 (s, C_{12}) ppm.

^{31}P{^{1}H}-NMR (283 MHz, DMSO-d_6+DCl, 300 K): not determined.

ATR-IR (neat): $\tilde{\nu}$ = 3048 (=C-H), 2934 (-C-H), 1728 (C=O), 1596 (arom. ring), 1430 (=C-P), 1175 (-C-O), 1096 (-C-O), 711 (monosubs. benzene), 688 (monosubs. benzene), 507 (P=S) cm^{-1}.

MS (HR-ESI positive): $C_{24}H_{25}NO_4PS$ $[M+H]^+$ requires 454.12364 m/z, found 454.12389 m/z (Δ = 0.55 ppm).

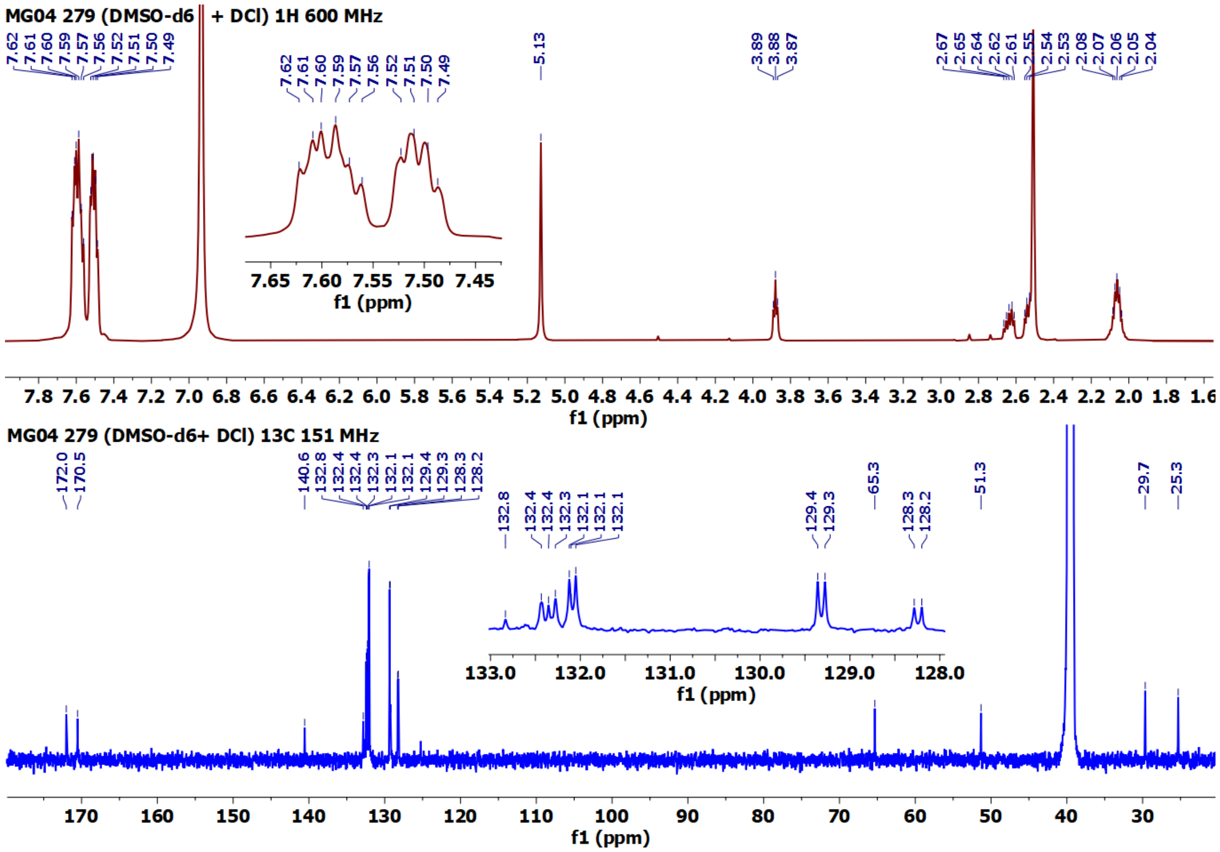

Figure S11: ^{1}H-NMR (red, 600 MHz) and ^{13}C-NMR (blue, 151 MHz) of ester (D)-**10** measured in DMSO-d_6 + DCl at 300 K. For the $^{13}C\{^{1}H\}$ spectrum, an exponential line broadening of 3 Hz was applied to improve the signal-to-noise ratio.

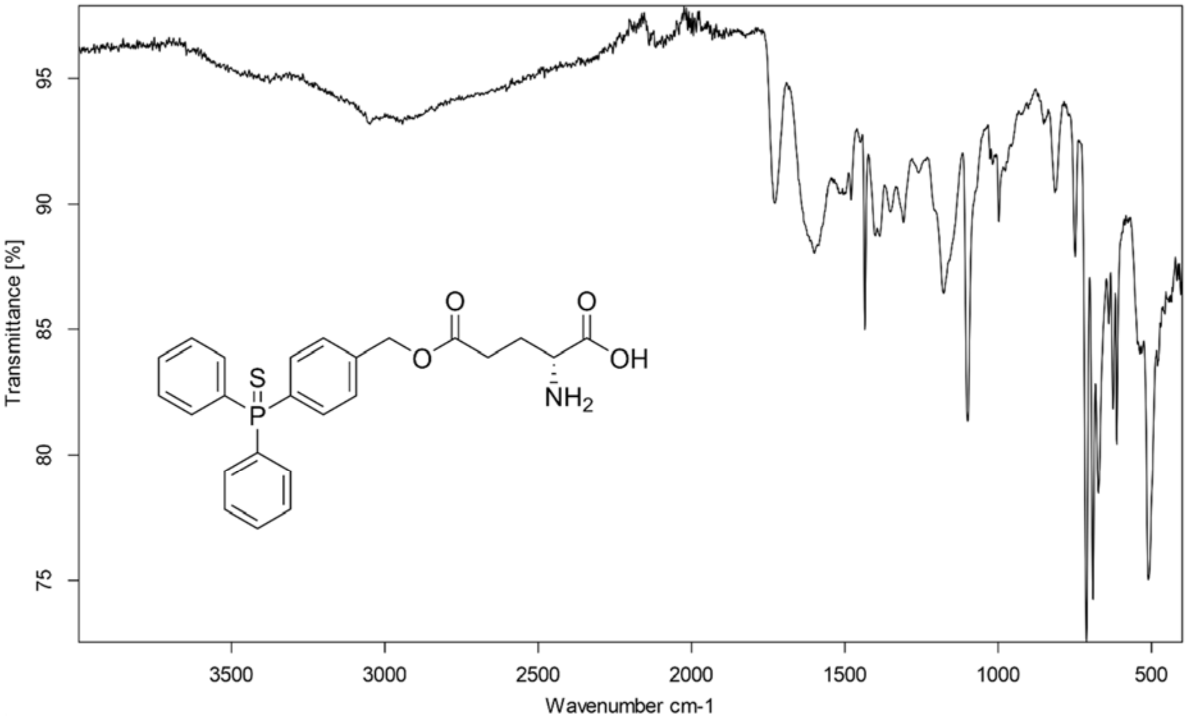

Figure S12: ATR-IR spectrum (neat) of ester (D)-**10**.

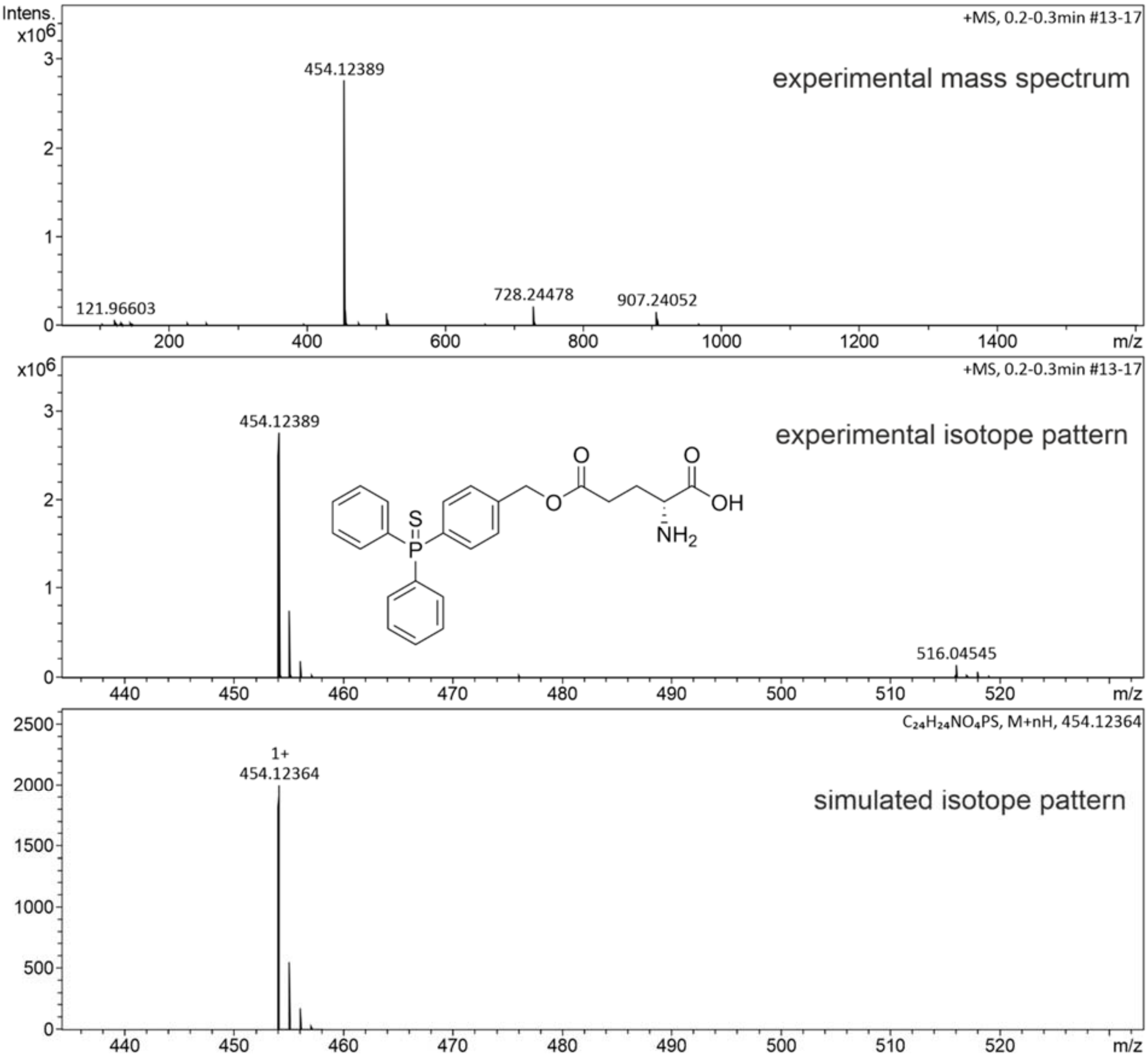

Figure S13: HR-MS (ESI positive) spectrum of ester (D)-**10**. The simulated/expected isotope pattern is compared with the experimental results.

3.10 Synthesis of 4-(diphenylphosphorothioyl)benzyl (L)-3-(2,5-dioxooxazolidin-4-yl)propanoate (DPPS-BLG-NCA)

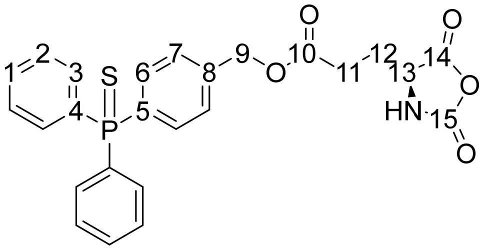

The synthesis is carried out according to a literature procedure.[44]

3.8 g DPPS-BLG ester (8.4 mmol, 1 eq.) is dissolved in 50 mL anhydrous THF. 5.38 mL (10.1 mmol, 1.2 eq.) of a phosgene solution (20 % in toluene) is added. The reaction mixture is stirred for 1 h, and the solvent is removed under reduced pressure. The crude product is purified by column chromatography under inert conditions (ethyl acetate:*n*-hexane 2:1), and 3.54 g (7.4 mmol, 88 % yield) of colorless crystals are obtained.

Internal batch numbers **MG04-105**, **MG04-157**, and **MG04-241**

$\boldsymbol{R_f}$ (ethyl acetate:*n*-hexane 2:1) = 0.22.

^{1}H-NMR (600 MHz, THF-d_8, 300 K): δ = 7.76-7.68 (m, 6$H_{3,6}$), 7.50 (m, 2H_1), 7.47-7.40 (m, 6$H_{2,7}$), 5.17 (s, 2H_9), 4.38 (ddd, $^4J_{HH}$ = 1 Hz, $^3J_{HH}$ = 6 Hz, $^3J_{HH}$ = 7 Hz, 1H_{13}), 2.54 (ddd, $^4J_{HH}$= 1 Hz, $^3J_{HH}$= 7 Hz, $^3J_{HH}$= ~8 Hz, 2H_{11}), 2.15-2.02 (m, 2H_{12}) ppm. Signals of residual ethyl acetate are visible in the spectrum.

^{13}C{^{1}H}-NMR (151 MHz, THF-d_8, 300 K): δ= 172.3 (C_{10}), 171.4 (C_{14}), 152.6 (C_{15}), 140.9 (d, $^4J_{PC}$ = 3 Hz, C_8), 134.7 (d, $^1J_{PC}$ = 85 Hz, C_4), 134.6 (d, $^1J_{PC}$ = 85 Hz, C_5), 133.3 (d, $^2J_{PC}$ = 11 Hz, C_6), 133.1 (d, $^2J_{PC}$ = 11 Hz, C_3), 132.2 (d, $^4J_{PC}$ = 3 Hz, C_1), 129.2 (d, $^3J_{PC}$ = 12 Hz, C_2), 128.5 (d, $^3J_{PC}$ = 12 Hz, C_7), 66.1 (d, $^5J_{PC}$ = 1 Hz, C_9), 57.3 (C_{13}), 29.8 (C_{11}), 27.9 (C_{12}) ppm. Signals of residual ethyl acetate are visible in the spectrum.

^{31}P{^{1}H}-NMR (243 MHz, THF-d_8, 300 K): δ = 41.8 ppm.

ATR-IR (neat): $\tilde{\nu}$ = 3286 (N-H), 1851 (C=O), 1781 (C=O), 1729 (C=O), 1434 (=C-P), 1163 (-C-O), 919 (=C-P), 717 (monosubs. benzene), 688 (monosubs. benzene) cm^{-1}.

MS (HR-ESI positive): $C_{25}H_{23}NO_5PS$ $[M+H]^+$ requires 480.10291 m/z, found 480.10288 m/z (Δ = 0.06 ppm).

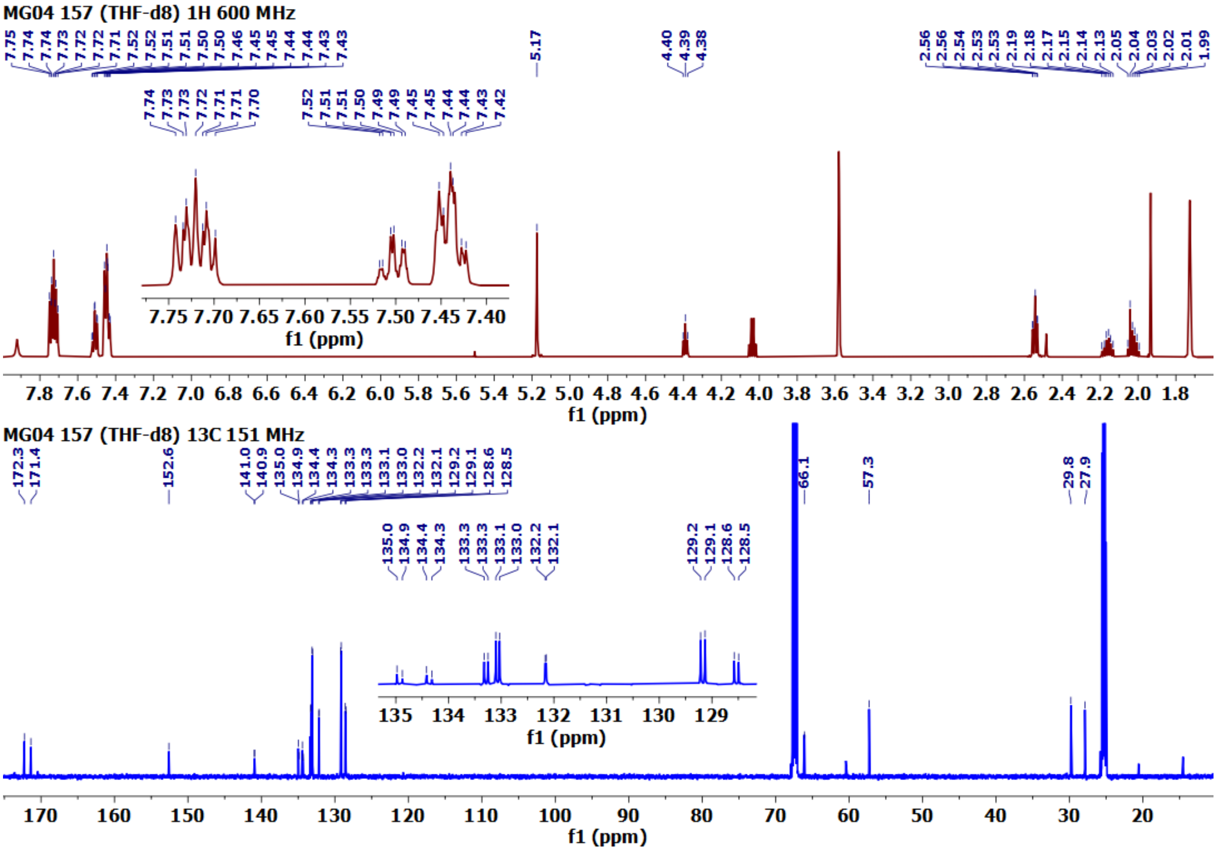

Figure S14: ^{1}H-NMR (red, 600 MHz) and ^{13}C-NMR (blue, 151 MHz) of NCA (L)-**11** measured in THF-d_8 at 300 K.

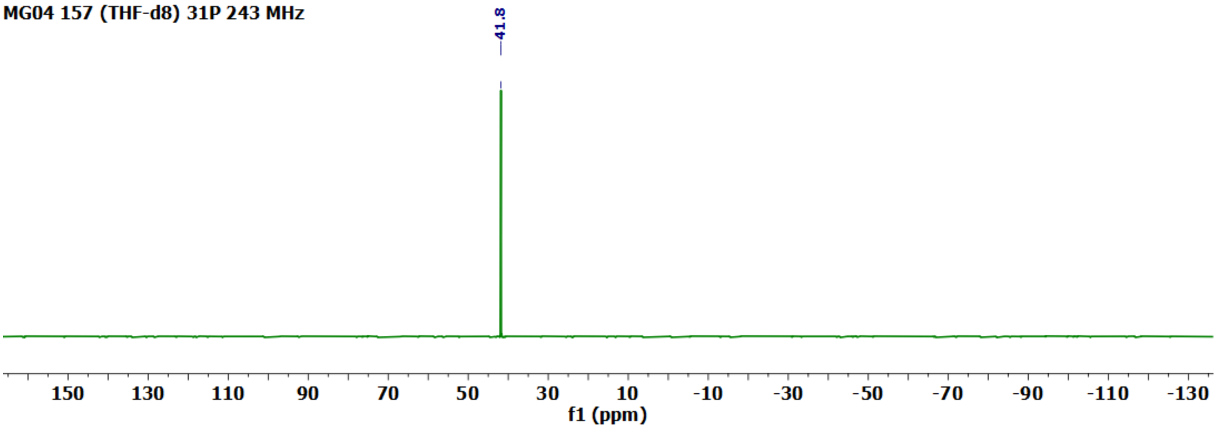

Figure S15: ^{31}P-NMR (green, 243 MHz) of NCA (L)-**11** measured in THF-d_8 at 300 K.

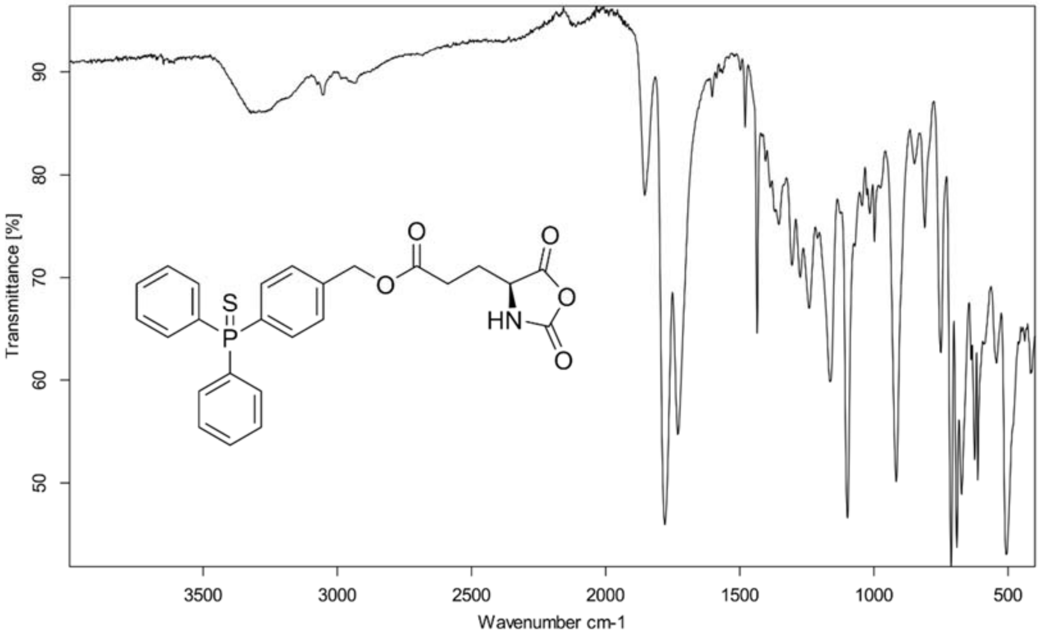

Figure S16: ATR-IR spectrum (neat) of NCA (L)-**11**.

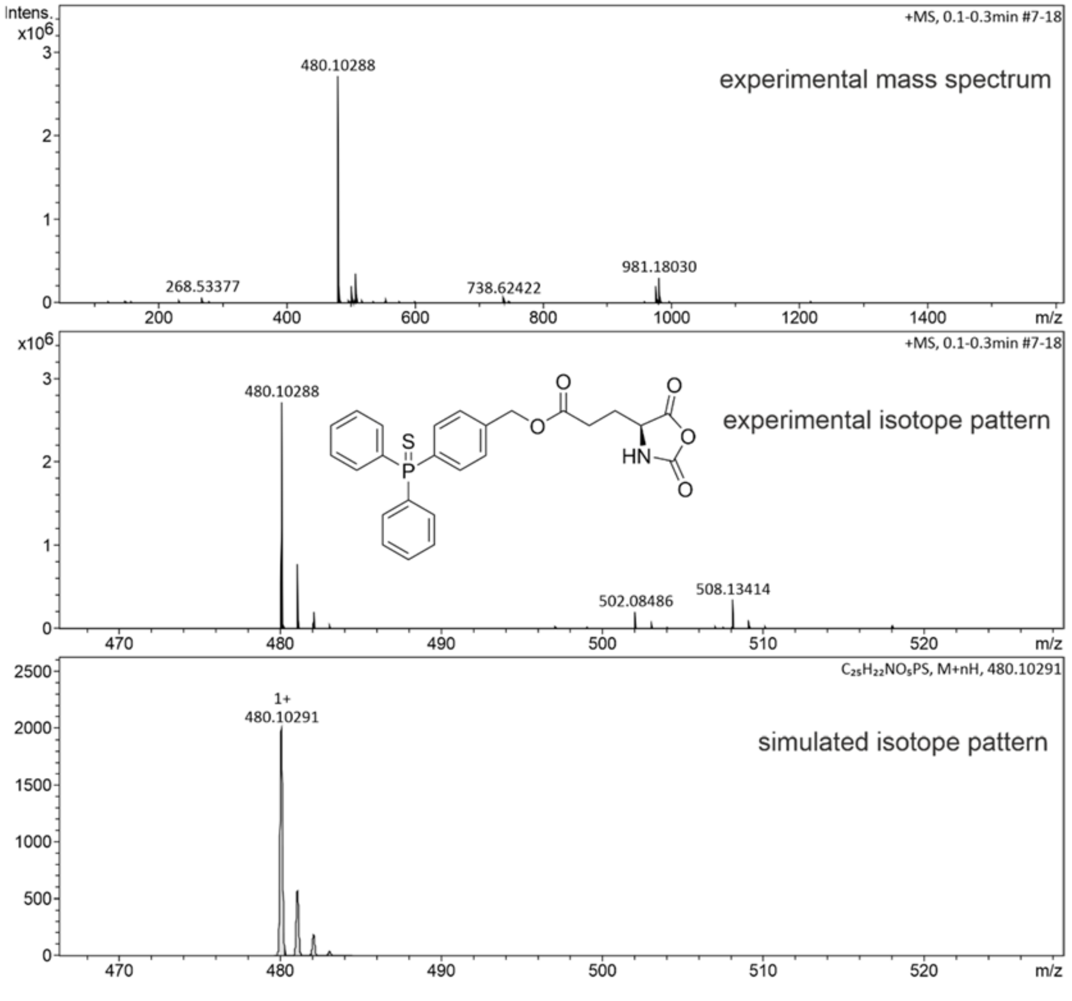

Figure S17: HR-MS (ESI positive) spectrum of NCA (L)-**11**. The simulated/expected isotope pattern is compared with the experimental results.

3.11 Synthesis of 4-(diphenylphosphorothioyl)benzyl (D)-3-(2,5-dioxooxazolidin-4-yl)propanoate (DPPS-BDG-NCA)

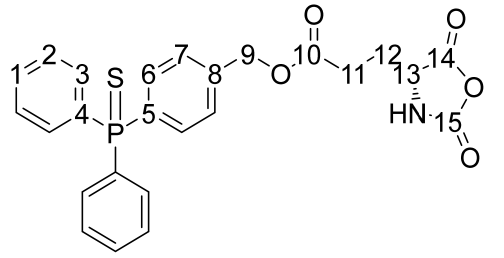

The synthesis is carried out according to a literature procedure.[44]

2.94 g DPPS-BDG ester (6.5 mmol, 1 eq.) is dissolved in 50 mL anhydrous THF. 5.38 mL (7.77 mmol, 1.2 eq.) of a phosgene solution (20 % in toluene) is added. The reaction mixture is stirred for 1 h, and the solvent is removed under reduced pressure. The crude product was purified by column chromatography under inert conditions (ethyl acetate:*n*-hexane 1:1). 1.00 g (2.1 mmol, 32 % yield) of colorless crystals were obtained.

Internal batch number **MG04-234**
$\boldsymbol{R_f}$ (ethyl acetate:*n*-hexane 1:1) = 0.13
^{1}H-NMR (600 MHz, $CDCl_3$, 300 K): δ = 7.76-7.68 (m, $6H_{3,6}$), 7.50 (dt, $^5J_{PH}$= ~2 Hz, $^3J_{HH}$ = 7.4 Hz, $2H_1$), 7.48-7.42 (ddd, $^3J_{HH}$ = 7.7 Hz, $^3J_{HH}$ = 7.7 Hz, $^4J_{PH}$ = 3 Hz, $4H_2$), 7.42-7.37 (dd, J = 8 Hz, J = 2 Hz, $2H_7$), 5.16 (s, $2H_9$), 4.36 (dd, $^3J_{HH}$ = 6 Hz, $^3J_{HH}$ = 6 Hz, $1H_{13}$), 2.56 (dd, $^3J_{HH}$ = 7 Hz, $^3J_{HH}$ = 7 Hz, $2H_{11}$), 2.30-2.06 (m, $2H_{12}$) ppm. Signals of residual ethyl acetate and THF are visible in the spectrum.
^{13}C{^{1}H}-NMR (151 MHz, $CDCl_3$, 300 K): δ = 172.2 (C_{10}), 169.5 (C_{14}), 151.7 (C_{15}), 149.1 (d, $^4J_{PC}$ = 3 Hz, C_8), 133.5 (d, $^1J_{PC}$ = 85 Hz, C_4), 132.8 (d, $^2J_{PC}$ = 11 Hz, C_6), 132.7 (d, $^1J_{PC}$ = 85 Hz, C_5, assigned via intensity), 132.3 (d, $^2J_{PC}$ = 11 Hz, C_3), 131.8 (d, $^4J_{PC}$ = 3 Hz, C_1), 128.7 (d, $^3J_{PC}$ = 13 Hz, C_2), 128.1 (d, $^3J_{PC}$ = 13 Hz, C_7), 66.2 (d, $^5J_{PC}$ = ~1 Hz, C_9), 57.9 (C_{13}), 29.7 (C_{11}), 26.9 (C_{12}) ppm. Signals of residual ethyl acetate and THF are visible in the spectrum.
^{31}P{^{1}H}-NMR (243 MHz, $CDCl_3$, 300 K): δ = 42.9 ppm.
ATR-IR (neat): $\tilde{\nu}$ = 3288 (N-H), 2956 (-C-H), 2869 (-C-H), 1850 (C=O), 1777 (C=O), 1733 (C=O), 1435 (=C-P), 1165 (-C-O), 921 (=C-P), 752 (*ortho*-subs. benzene), 714 (monosubs. benzene), 692 (monosubs. benzene), 626 (P=S) cm^{-1}.
MS (HR-ESI positive): $C_{25}H_{23}NO_5PS$ $[M+H]^+$ requires 480.10291 m/z, found 480.10332 m/z (Δ = 0.85 ppm).

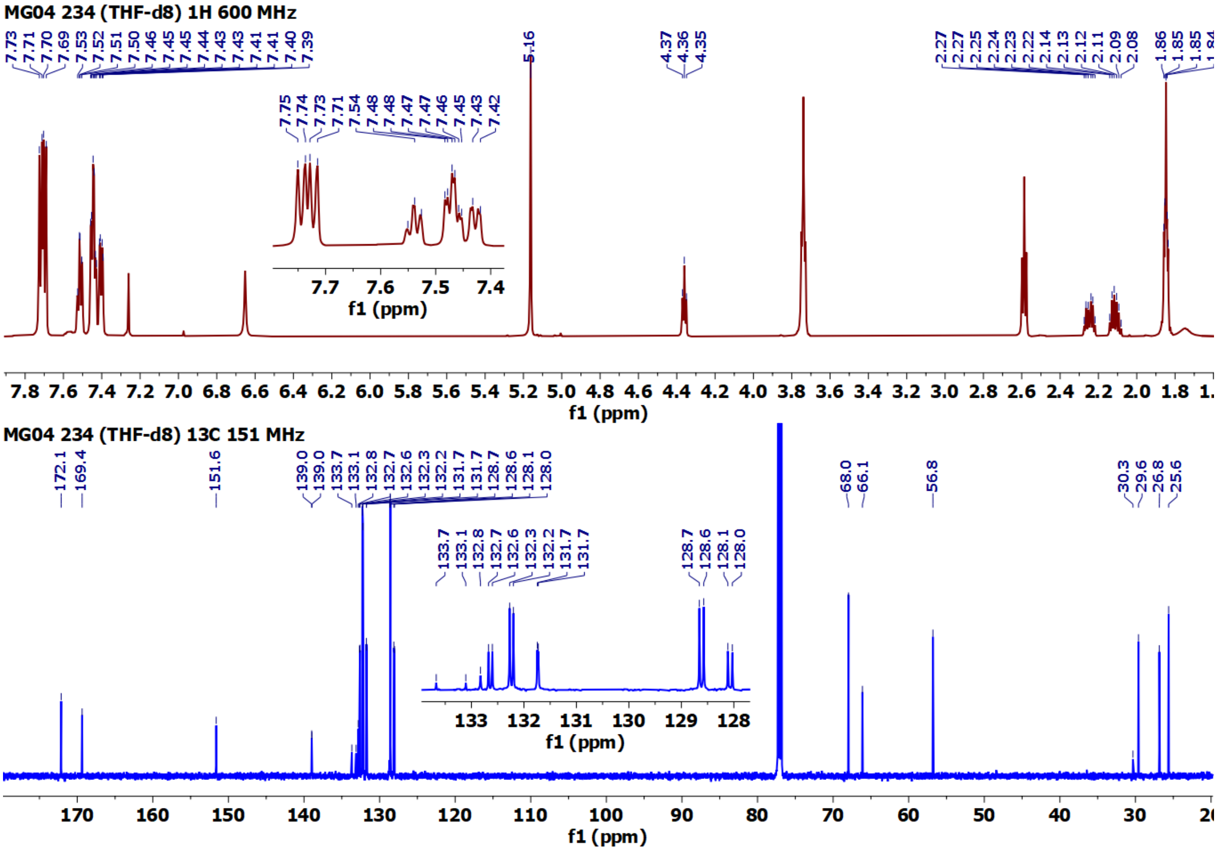

Figure S18: ^{1}H-NMR (red, 600 MHz) and ^{13}C-NMR (blue, 151 MHz) of NCA (D)-**11** measured in THF-d_8 at 300 K.

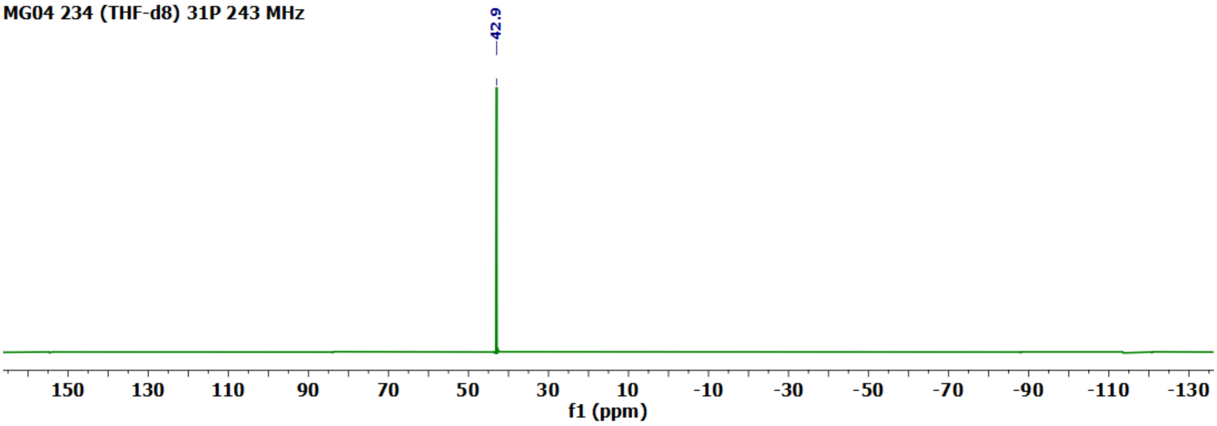

Figure S19: ^{31}P-NMR (green, 243 MHz) of NCA (D)-**11** measured in THF-d_8 at 300 K.

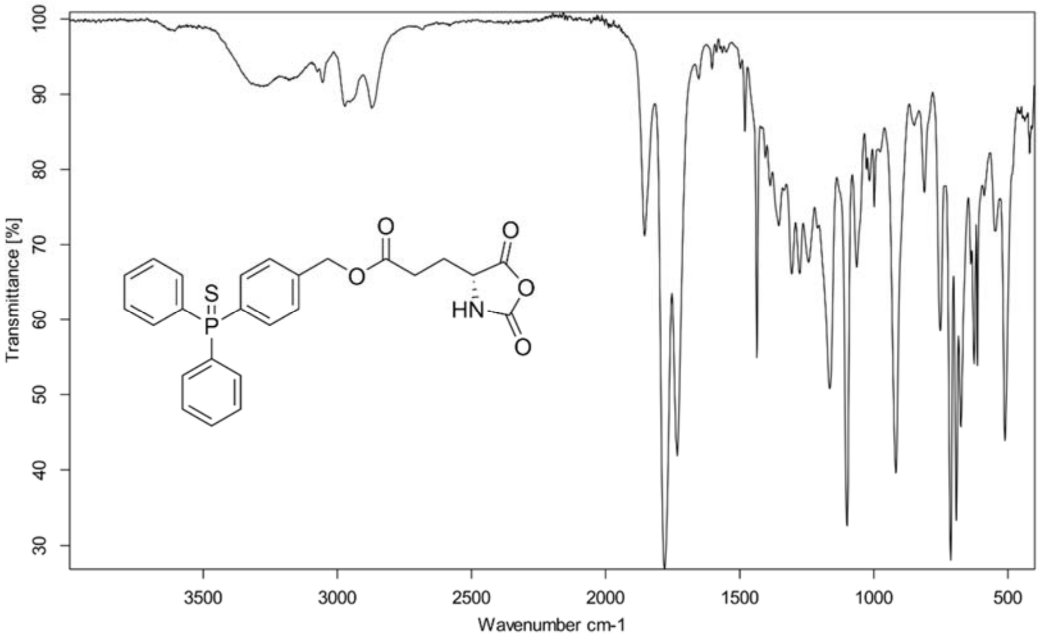

Figure S20: ATR-IR spectrum (neat) of NCA (D)-**11**.

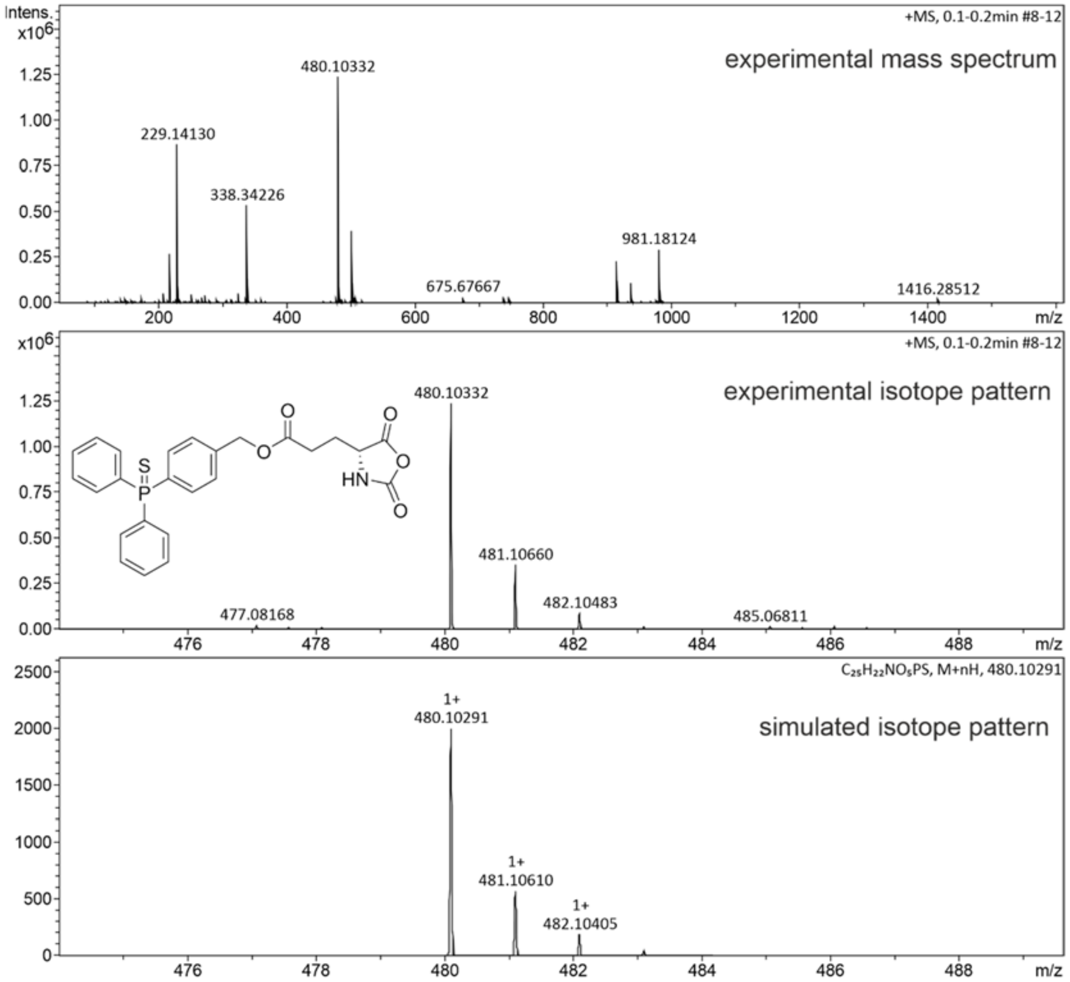

Figure S21: HR-MS (ESI positive) spectrum of NCA (D)-**11**. The simulated/expected isotope pattern is compared with the experimental results.

3.12 Synthesis of the polymer DPPS-PBLG

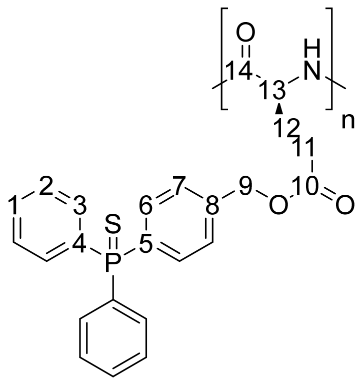

The synthesis is carried out according to a literature procedure.[45]

1.12 g DPPS-BLG-NCA (2.34 mmol, 1 eq.) is dissolved in 40 mL anhydrous and degassed THF (c = 0.1 mol/L) inside a glove box operating under argon. 78 µL of a stock solution containing 10 µL per 1 mL degassed anhydrous THF (resulting in 0.78 µL of pure DMEA, 0.0078 mmol, 0.0033 eq.) is added, resulting in a monomer/initiator ratio of 300:1. The reaction is stirred for 20 h. The reaction progress is monitored using IR spectroscopy. After complete conversion, the polymer solution is precipitated in MeOH (+ 1 % aq. HCl) and hexane thrice each. The solvents are removed under reduced pressure. 821 mg (81 % yield) of colorless polymer fibers are obtained.

Internal batch numbers **MG04-158** and **MG04-223**

^{1}H-NMR (L-polymer, 700 MHz, $CDCl_3$+ TFA-*d*, 300 K): δ = 7.70 - 7.57 (m, 6$H_{3,6}$), 7.51 (t, $^3J_{HH}$ = 7.3 Hz, 2H_1), 7.42 (dt, ^3J = 7.8 Hz, J = 2.8 Hz, 4H_2), 7.34 (dd, ^3J = 8 Hz, J = ~2 Hz, 2H_7), 5.14 (d, $^2J_{HH}$ = 12.8 Hz, 1H_9), 5.09 (d, $^2J_{HH}$ = 12.8 Hz, 1H_9), 4.63 (dd, J_{HH} = 4 Hz, $^3J_{HH}$ = 8.5 Hz, 1H_{13}), 2.55 (m, 2H_{11}), 2.07 (m, 2H_{12}) ppm.

^{13}C{^{1}H}-NMR (L-polymer, 176 MHz, $CDCl_3$+TFA-*d*, 300 K): δ= 175.0 (C_{10}), 172.9 (C_{14}), 138.9 (d, $^4J_{PC}$ = 2 Hz, C_8), 132.6 (d, $^2J_{PC}$ = 11 Hz, C_6), 132.3 (d, $^4J_{PC}$ = 2 Hz, C_1), 132.2 (d, $^2J_{PC}$ = 11 Hz, C_3), 131.6 (d, $^1J_{PC}$ = 86 Hz, C_5), 130.5 (d, $^1J_{PC}$ = 86 Hz, C_4), 128.7 (d, $^3J_{PC}$ = 13 Hz, C_2), 127.9 (d, $^3J_{PC}$ = 13 Hz, C_7), 66.8 (C_9), 53.2 (C_{13}), 29.9 (C_{11}), 27.0 (C_{12}) ppm.

^{31}P{^{1}H}-NMR (L-polymer, 283 MHz, $CDCl_3$+TFA-*d*, 300 K): δ = 43.9 ppm.

ATR-IR (neat): $\tilde{\nu}$ = 3285 (N-H, H-bridge), 3052 (=C-H), 2949 (-C-H), 2867 (-C-H), 1737 (C=O), 1649 (C=O), 1545 (C=C), 1432 (=C-P), 1158 (-C-O), 1097 (-C-O), 709 (monosubs. benzene), 689 (monosubs. benzene), 508 (P=S) cm^{-1}.

MS (MALDI TOF): Repeating unit $C_{24}H_{22}NO_3PS$ requires 435.5 m/z, found 435.5 ± 0.8 m/z.

SEC (batch: MG04-223, M:I = 300:1): $\overline{M}_n = 1.804 \cdot 10^5$ gmol^{-1}, $\overline{M}_w = 6.082 \cdot 10^5$ gmol^{-1}, $Đ = 3.37$.

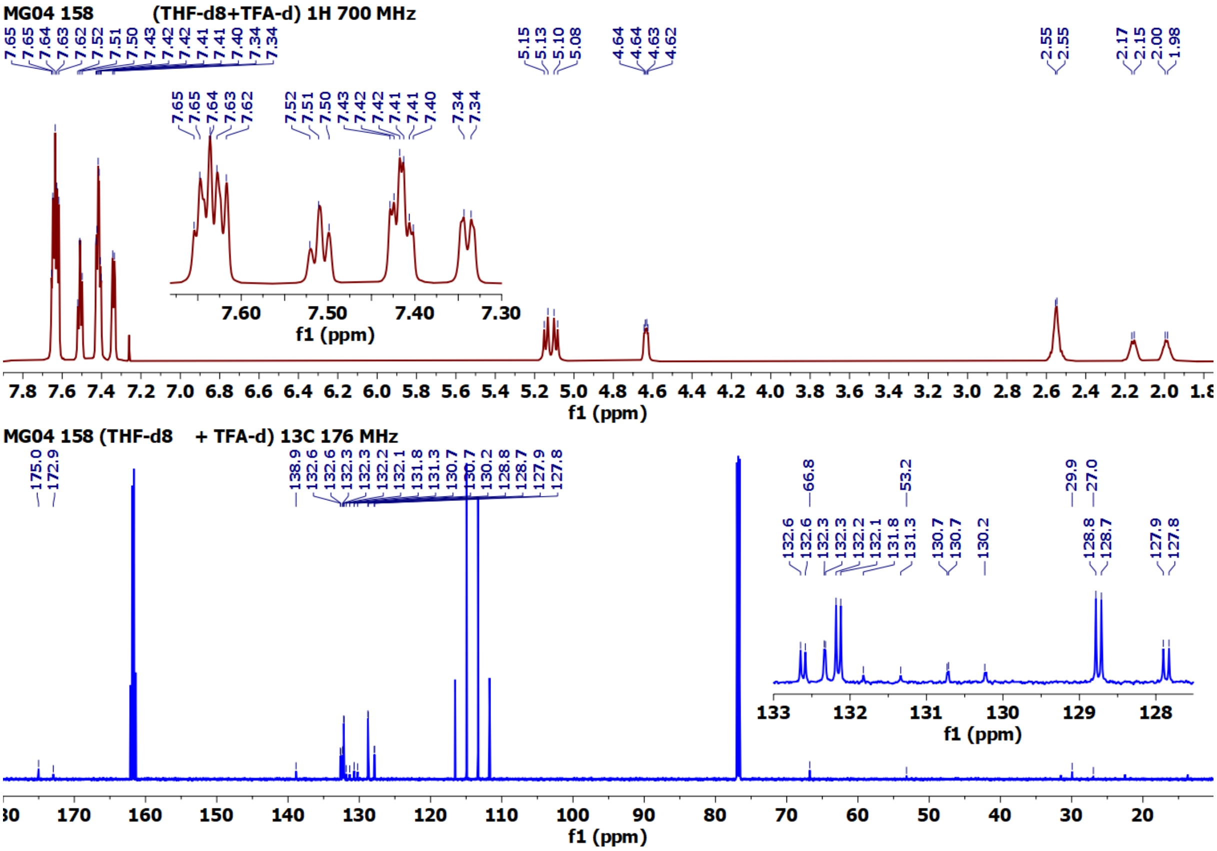

Figure S22: ^{1}H-NMR (red, 700 MHz) and ^{13}C-NMR (blue, 176 MHz) of polymer DPPS-PBLG (L)-**12** measured in THF-d_8 + TFA-*d* at 300 K.

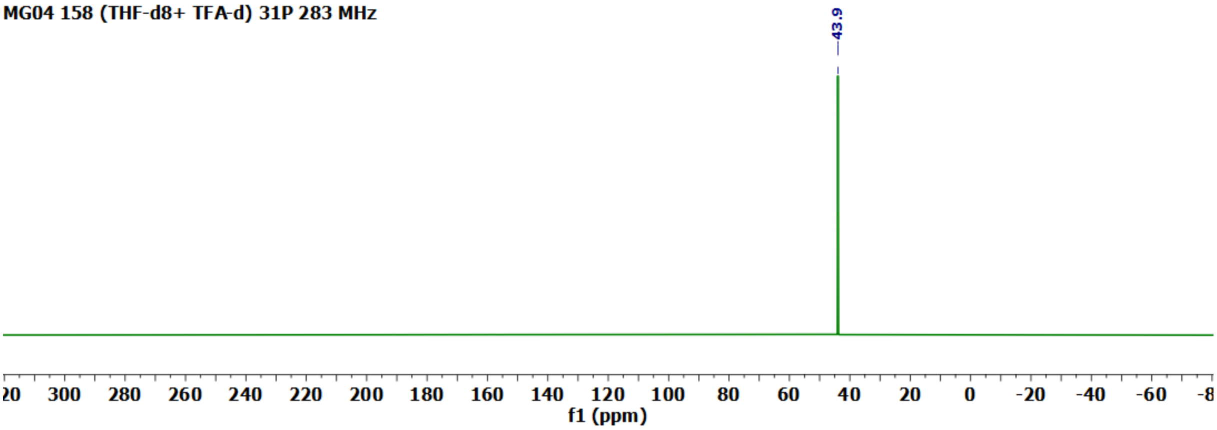

Figure S23: ^{31}P-NMR (green, 283 MHz) of polymer DPPS-PBLG (L)-**12** measured in THF-d_8 + TFA-*d* at 300 K.

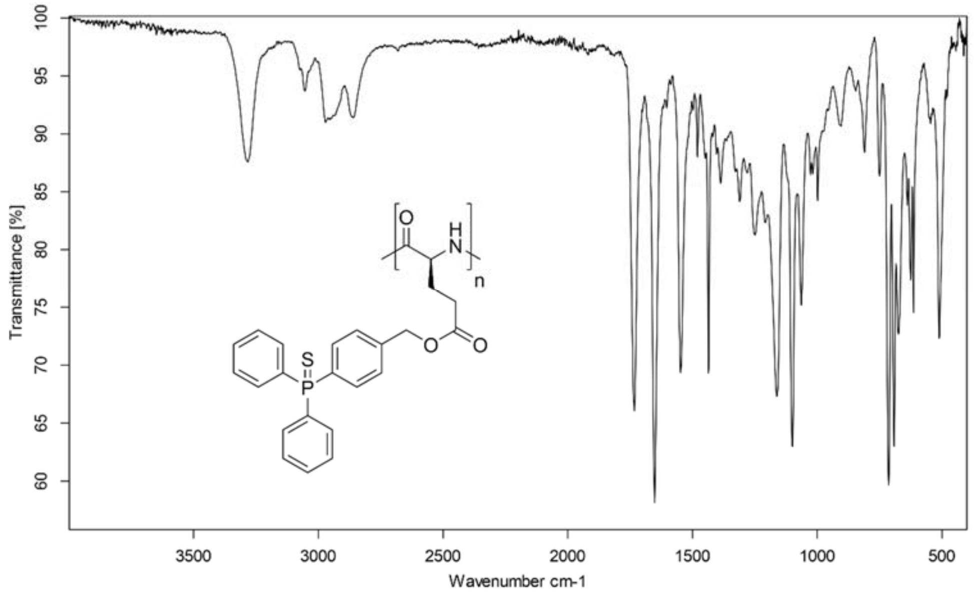

Figure S24: ATR-IR spectrum (neat) of polymer DPPS-PBLG (L)-**12**.

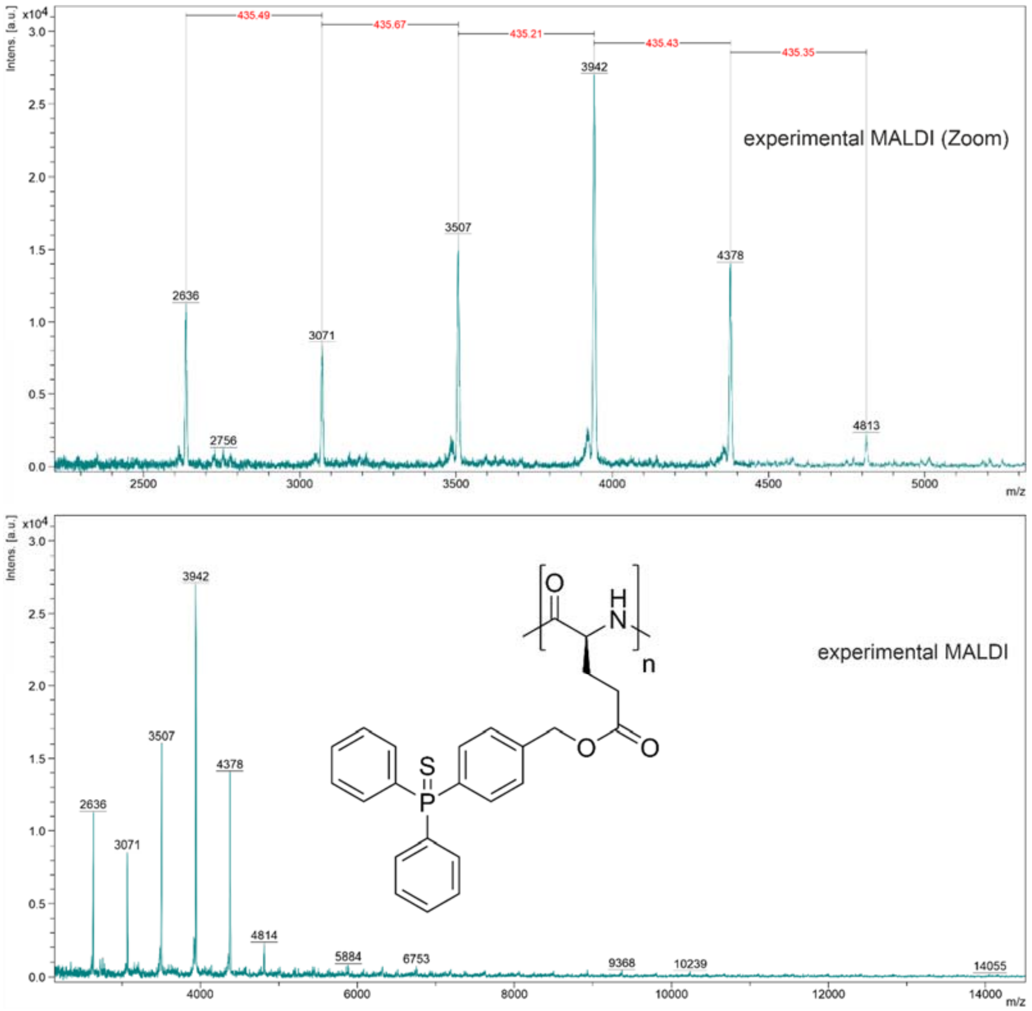

Figure S25: MALDI-TOF-MS spectrum of polymer DPPS-PBLG (L)-**12**. The mass difference between peaks (in red) corresponds to the molecular weight of the repeating unit.

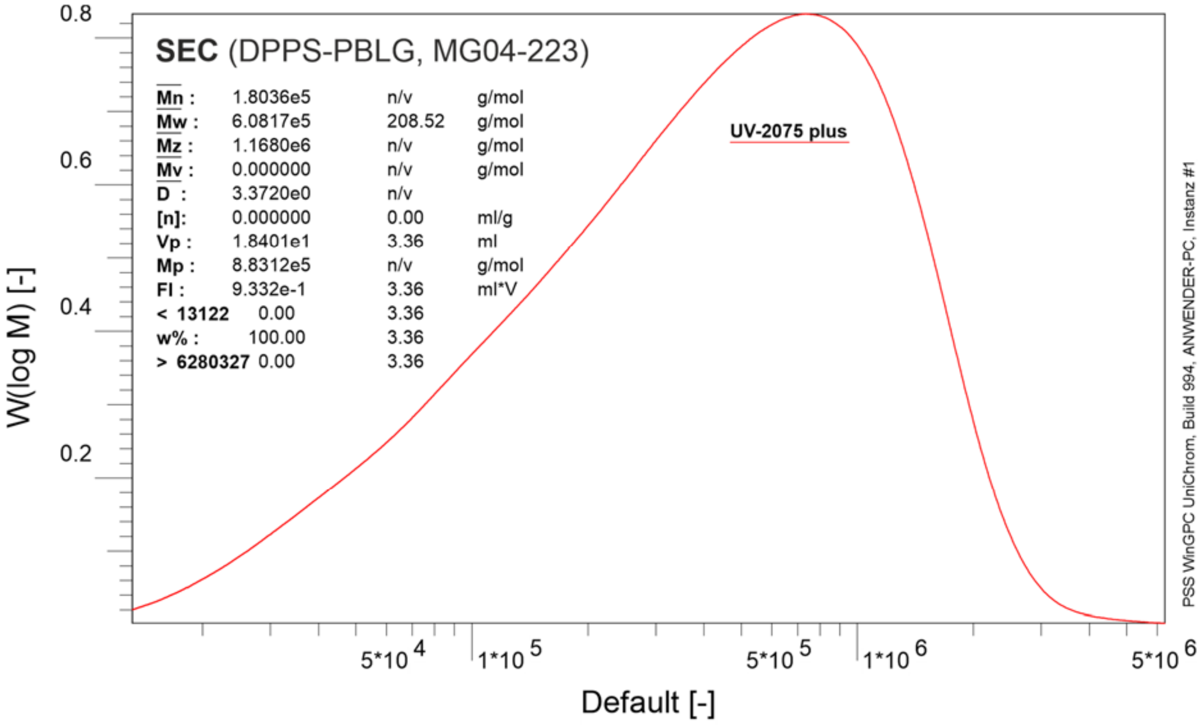

Figure S26: SEC graph of DPPS-PBLG (internal batch number MG04-223). Detailed information about the acquisition parameters can be found in SI section 2.2.

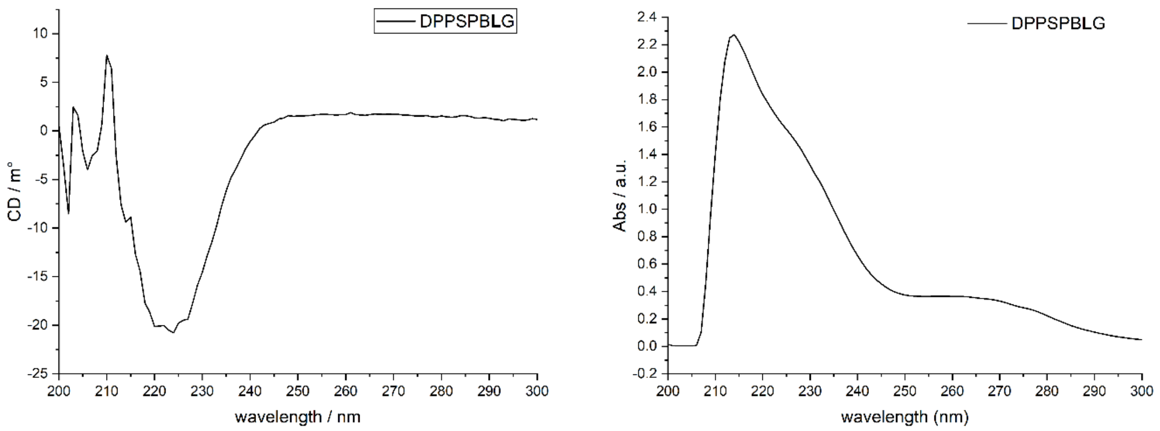

Figure S27: On the left side, the CD spectrum of DPPS-PBLG dissolved in chloroform (2.5 mg/mL), and on the right side, the respective absorbance spectrum is shown. The measurement is performed in custom-made demountable cuvettes with a path length of $d \sim 0.01$ mm.[5] At around 215 nm, the UV solvent cut-off for chloroform is reached (visible on the right).

3.13 Synthesis of the polymer DPPS-PBDG

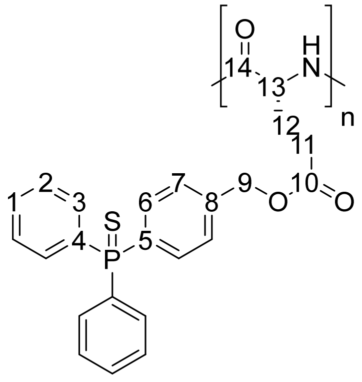

The synthesis is carried out according to a literature procedure.[45]

1.00 g DPPS-BDG-NCA (2.09 mmol, 1 eq.) is dissolved in 40 mL anhydrous and degassed THF (c = 0.1 mol/L) inside a glove box operating under argon. 70 µL of a stock solution containing 10 µL per 1 mL degassed anhydrous THF (resulting in 0.70 µL of pure DMEA, 0.0070 mmol, 0.0033 eq.) is added, resulting in a monomer/initiator ratio of 300:1. The reaction is stirred for 20 h. The reaction progress is monitored using IR spectroscopy. After complete conversion, the polymer solution was precipitated in MeOH (+ 1 % aq. HCl) and hexane thrice each. Residual solvents are removed under reduced pressure. 690 mg (76 % yield) of colorless polymer fibers are obtained.

Internal batch number **MG04-235**

^{1}H-NMR (D-polymer, 700 MHz, $CDCl_3$+TFA-*d*, 300 K): δ = 7.70- 7.55 (m, $6H_{3,6}$), 7.50 (t, $^{3}J_{HH}$ = 6.8 Hz, $2H_1$), 7.42 (t, ^{3}J = ~7 Hz, $4H_2$), 7.33 (d, ^{3}J = 6.8 Hz, $2H_7$), 5.13 (d, $^{2}J_{HH}$ = 12.8 Hz, $1H_9$), 5.08 (d, $^{2}J_{HH}$ = 12.8 Hz, $1H_9$), 4.62 (m, $1H_{13}$), 2.53 (m, $2H_{11}$), 2.06 (m, $2H_{12}$) ppm.

^{13}C{^{1}H}-NMR (D-polymer, 176 MHz, $CDCl_3$+TFA-*d*, 300 K): δ = 174.4 (C_{10}), 172.3 (C_{14}), 138.3 (C_8), 132.1 (d, $^{2}J_{PC}$ = 11 Hz, C_6), 131.7 (C_1), 131.6 (d, $^{2}J_{PC}$ = 11 Hz, C_3), 128.2 (d, $^{3}J_{PC}$ = 13 Hz, C_2), 127.3 (d, $^{3}J_{PC}$ = 13 Hz, C_7), 66.2 (C_9), 52.6 (C_{13}), 29.3 (C_{11}), 26.4 (C_{12}) ppm.

The broad signals between 130.5 and 129.5 ppm are assigned to C_4 and C_5, but no exact ^{13}C chemical shift or ^{13}C-^{31}P coupling constants can be determined.

^{31}P{^{1}H}-NMR (D-polymer, 243 MHz, $CDCl_3$+TFA-*d*, 300 K): δ = 43.9 ppm.

ATR-IR (neat): $\tilde{\nu}$ = 3282 (N-H, H-bridge), 2964 (-C-H), 2859 (-C-H), 1734 (C=O), 1653 (C=O), 1548 (C=C), 1438 (=C-P), 1159 (-C-O), 1102 (-C-O), 712 (monosubs. benzene), 689 (monosubs. benzene), 508 (P=S) cm^{-1}.

MS (MALDI TOF): Repeating unit $C_{24}H_{22}NO_3PS$ requires 435.5 m/z, found 435.5 ± 0.8 m/z.

SEC (batch: MG04-223, M:I = 300:1): $\overline{M}_n = 2.343 \cdot 10^5$ $gmol^{-1}$, $\overline{M}_w = 9.869 \cdot 10^5$ $gmol^{-1}$, $Đ$ = 4.21.

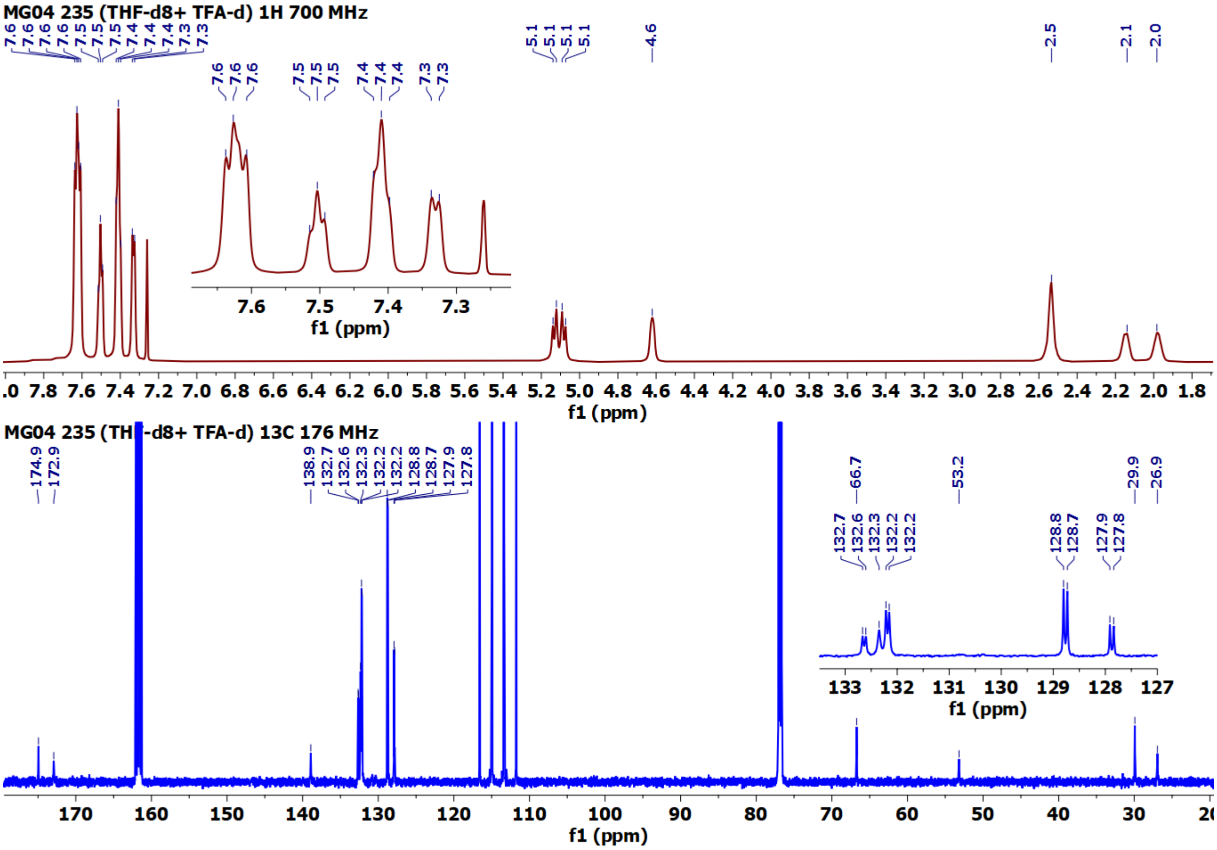

Figure S28: ^{1}H-NMR (red, 700 MHz) and ^{13}C-NMR (blue, 176 MHz) of polymer DPPS-PBDG (D)-**12** measured in THF-d_8 + TFA-d at 300 K.

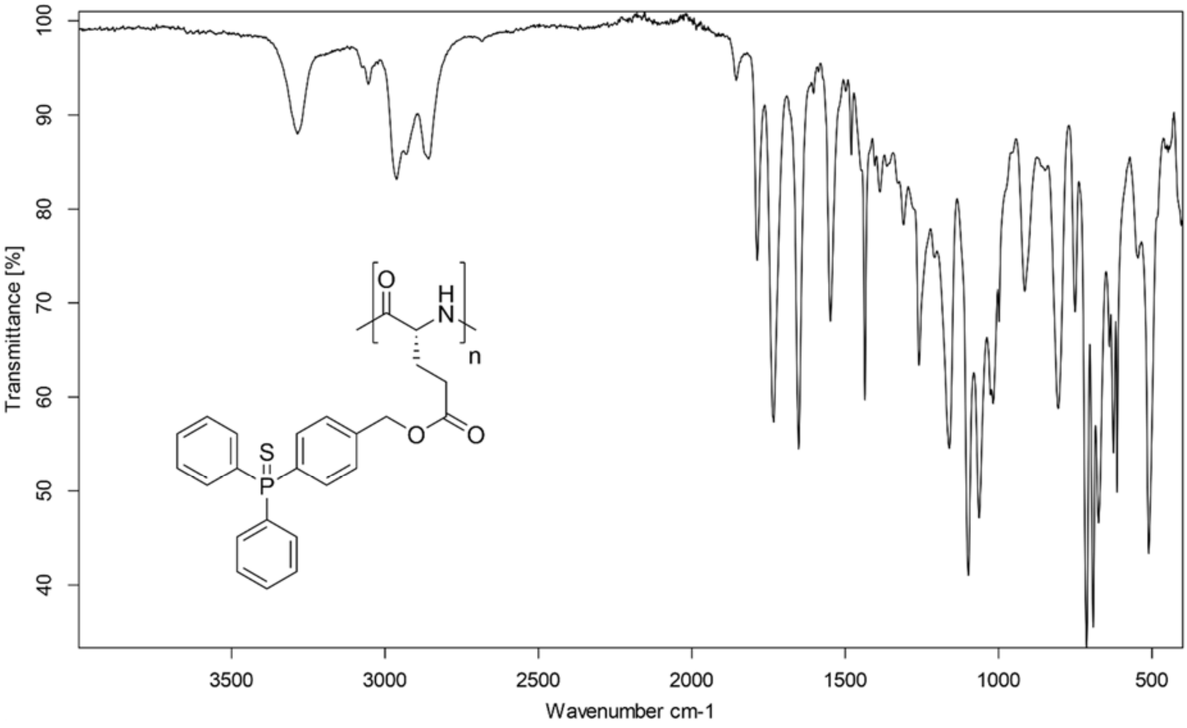

Figure S29: ATR-IR spectrum (neat) of polymer DPPS-PBDG (D)-**12**.

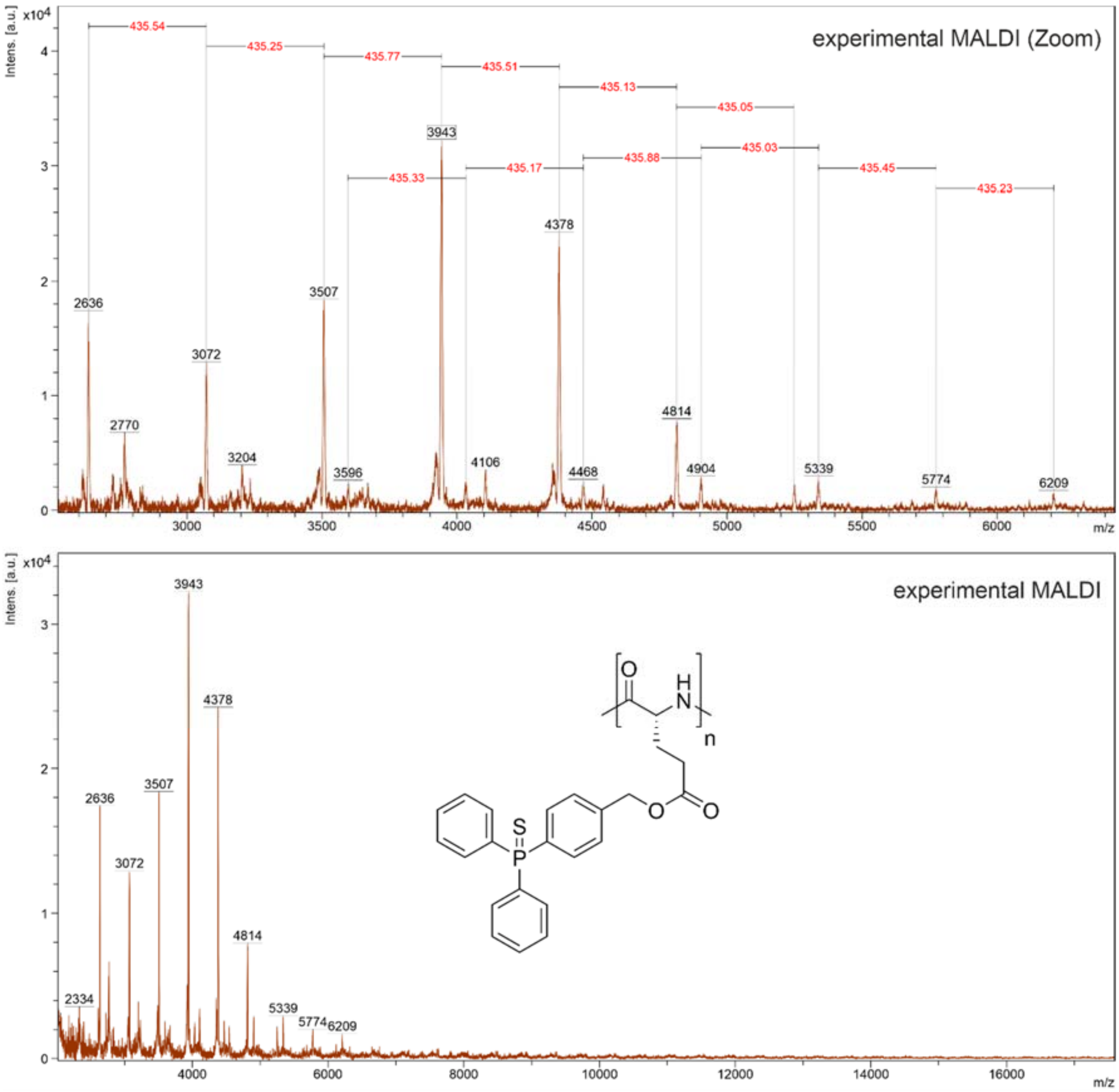

Figure S30: MALDI-TOF-MS spectrum of polymer DPPS-PBDG (D)-**12**. The mass difference between peaks (in red) corresponds to the molecular weight of the repeating unit.

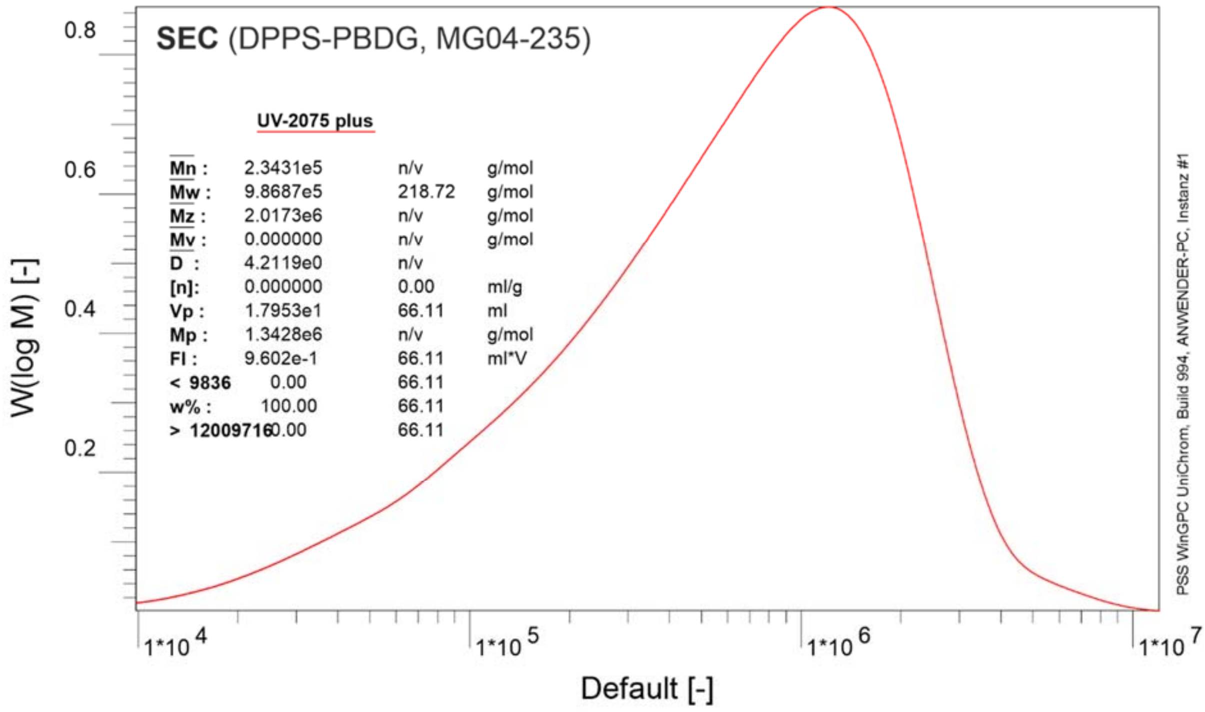

Figure S31: SEC graph of DPPS-PBDG (internal batch number MG04-235). Detailed information about the acquisition parameters can be found in SI section 2.2.

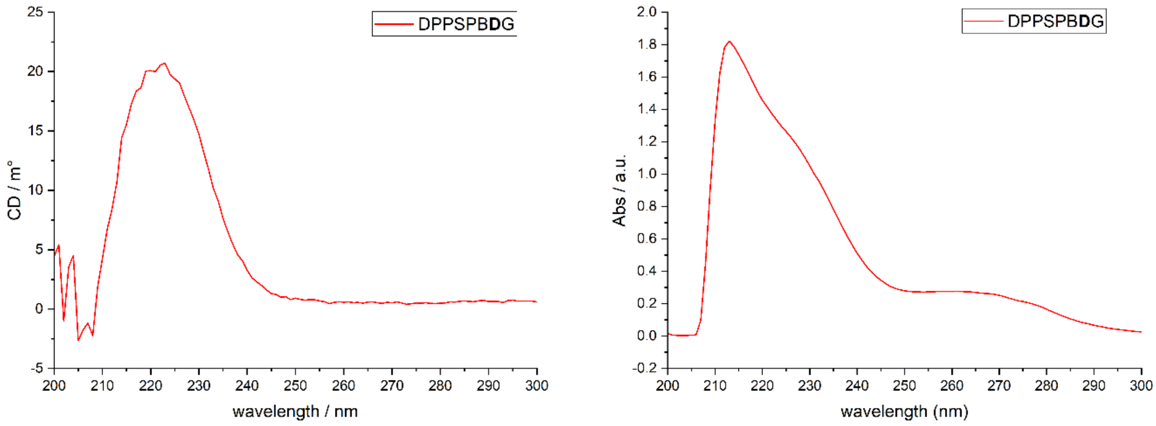

Figure S32: On the left side, the CD spectrum of DPPS-PBDG dissolved in chloroform (2.5 mg/mL), and on the right side, the respective absorbance spectrum is shown. The measurement is performed in custom-made demountable cuvettes with a path length of d ~ 0.01 mm.[5] At around 215 nm, the UV solvent cut-off for chloroform is reached (visible on the right).

4. NMR (coupling) data of the analytes

4.1 (-)-Isopinocampheol

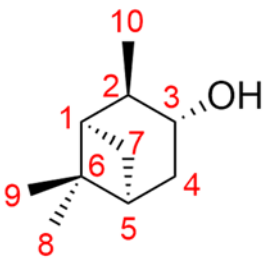

Figure S33: (-)-Isopinocampheol (IPC) with numbered atom positions. The diastereotopic CH_2 protons are labeled 4s, 4a, 7s, and 7a, with 's' defined as *syn* and 'a' as *anti* relative to the dimethyl bridge (C6, C8, C9).[14]

Table S4: Assignment of (-)-IPC in $CDCl_3$ at 700 MHz (^{1}H freq.) and 300 K.[14,46,47]

C	H	δC / ppm	δH / ppm
1	1	47.82	1.79
2	2	47.59	1.94
3	3	71.78	4.07
4	4s	38.89	2.50
4	4a	38.89	1.71
5	5	41.75	1.93
6	-	38.14	-
7	7s	34.34	2.36
7	7a	34.34	1.03
8	8	27.64	1.21
9	9	23.67	0.91
10	10	20.70	1.12

Table S5: Scalar couplings ($^1J_{CH}$) of (-)-IPC in $CDCl_3$ at 700 MHz (^1H freq.) and 300 K.

		perf. CLIP		F_1-cpl		F_1-cpl MQEvo	
C	H	$^1J_{CH}$ / Hz	Δ^1J_{CH} / Hz	$^1J_{CH}$ / Hz	Δ^1J_{CH} / Hz	$^1J_{CH}$ / Hz	Δ^1J_{CH} / Hz
1	1	141.26	0.11	141.39	0.08	-	-
2	2	126.79	0.10	126.73	0.04	-	-
3	3	142.29	0.08	142.32	0.04	-	-
4	4s	126.73	0.12	253.70*	0.03	126.67	0.07
4	4a	126.94	0.13	253.66*	0.04	127.01	0.09
5	5	142.03	0.20	141.76	0.07	-	-
6	-	-	-	-	-	-	-
7	7s	135.32	0.07	272.21*	0.04	135.05	0.08
7	7a	137.05	0.07	272.28*	0.06	137.18	0.07
8	8	124.66	0.04	124.75	0.04	-	-
9	9	123.68	0.02	123.77	0.06	-	-
10	10	124.91	0.05	125.11	0.10	-	-

*Only the sum of the individual $^1J_{CH}$ of a methylene group is observable.

Table S6: Total couplings ($^1T_{CH}$) of (-)-IPC in 10.62 % (w/w) DPPS-PBLG/$CDCl_3$ at 700 MHz (^1H freq.) and 300 K.

		perf. CLIP		F_1-cpl		F_1-cpl MQEvo	
C	H	$^1T_{CH}$ / Hz	Δ^1T_{CH} / Hz	$^1T_{CH}$ / Hz	Δ^1T_{CH} / Hz	$^1T_{CH}$ / Hz	Δ^1T_{CH} / Hz
1	1	156.21	0.35	155.32	0.04	-	-
2	2	117.65	0.28	117.36	0.07	-	-
3	3	148.61	0.18	148.47	0.06	-	-
4	4s	131.53	0.24	253.25*	0.04	131.02	0.12
4	4a	122.15	0.29	253.18*	0.05	122.35	0.11
5	5	117.61	0.23	116.78	0.11	-	-
6	-	-	-	-	-	-	-
7	7s	157.97	0.16	288.39*	0.06	157.49	0.08
7	7a	131.27	0.15	288.49*	0.08	131.04	0.05
8	8	120.38	0.26	120.11	0.03	-	-
9	9	122.15	0.12	122.07	0.06	-	-
10	10	130.02	0.19	129.97	0.04	-	-

*Only the sum of the individual $^1T_{CH}$ of a methylene group is observable.

Table S7: Total couplings ($^1T_{CH}$) of (-)-IPC in 10.67 % (w/w) DPPS-PBDG/$CDCl_3$ at 700 MHz (^{1}H freq.) and 300 K.

		perf. CLIP		F_1-cpl		F_1-cpl MQEvo	
C	H	$^1T_{CH}$ / Hz	Δ^1T_{CH} / Hz	$^1T_{CH}$ / Hz	Δ^1T_{CH} / Hz	$^1T_{CH}$ / Hz	Δ^1T_{CH} / Hz
1	1	155.36	0.23	154.44	0.04	-	-
2	2	122.64	0.29	122.29	0.09	-	-
3	3	149.49	0.18	149.35	0.05	-	-
4	4s	137.69	0.19	257.16*	0.06	136.93	0.15
4	4a	119.93	0.19	257.13*	0.05	120.09	0.10
5	5	119.44	0.24	118.81	0.11	-	-
6	-	-	-	-	-	-	-
7	7s	153.74	0.15	284.56*	0.04	153.37	0.12
7	7a	131.84	0.15	284.51*	0.06	131.61	0.11
8	8	121.57	0.12	121.42	0.02	-	-
9	9	121.87	0.11	121.78	0.07	-	-
10	10	130.77	0.13	130.73	0.06	-	-

*Only the sum of the individual $^1T_{CH}$ of a methylene group is observable.

Table S8: RDCs ($^1D_{CH}$/$^1D_{CC}$) of (-)-IPC calculated from the scalar couplings ($^1J_{CH}$) in isotropic $CDCl_3$ (**Table S5**) and the total couplings ($^1T_{CH}$) in 10.62 % (w/w) DPPS-PBLG/$CDCl_3$ (**Table S6**) at 700 MHz (^{1}H freq.) and 300 K.

		perf. CLIP		F_1-cpl		F_1-cpl MQEvo	
C	X=H,C	$^1D_{CX}$ / Hz	Δ^1D_{CX} / Hz	$^1D_{CX}$ / Hz	Δ^1D_{CX} / Hz	$^1D_{CX}$ / Hz	Δ^1D_{CX} / Hz
1	H1	7.48	0.46	6.97	0.11	-	-
C2	H2	-4.57	0.37	-4.68	0.11	-	-
C3	H3	3.16	0.26	3.07	0.10	-	-
C4	H4s	2.40	0.36	-	-	2.17	0.19
C4	H4a	-2.40	0.42	-	-	-2.33	0.19
C5	H5	-12.21	0.43	-12.49	0.19	-	-
C6	-	-	-	-	-	-	-
C7	H7s	11.33	0.23	-	-	11.22	0.16
C7	H7a	-2.89	0.22	-	-	-3.07	0.13
C8	C6	0.61	0.30	0.66	0.07	-	-
C9	C6	0.22	0.14	0.24	0.11	-	-
C10	C2	-0.73	0.24	-0.70	0.15	-	-

Table S9: RDCs ($^1D_{CH}$/$^1D_{CC}$) of (-)-IPC calculated from the scalar couplings ($^1J_{CH}$) in isotropic $CDCl_3$ (**Table S5**) and the total couplings ($^1T_{CH}$) in 10.67 % (w/w) DPPS-PBDG/$CDCl_3$ (**Table S7**) at 700 MHz (^{1}H freq.) and 300 K.

		***perf.* CLIP**		**F_1-cpl**		**F_1-cpl MQEvo**	
**C**	**X=H,C**	**$^1D_{CX}$ / Hz**	**Δ^1D_{CX} / Hz**	**$^1D_{CX}$ / Hz**	**Δ^1D_{CX} / Hz**	**$^1D_{CX}$ / Hz**	**Δ^1D_{CX} / Hz**
C1	H1	7.05	0.34	6.53	0.11	-	-
C2	H2	-2.08	0.39	-2.22	0.12	-	-
C3	H3	3.60	0.25	3.52	0.09	-	-
C4	H4s	5.48	0.31	-	-	5.13	0.22
C4	H4a	-3.51	0.32	-	-	-3.46	0.18
C5	H5	-11.29	0.43	-11.48	0.18	-	-
C6	-	-	-	-	-	-	-
C7	H7s	9.21	0.22	-	-	9.16	0.20
C7	H7a	-2.60	0.22	-	-	-2.78	0.19
C8	C6	-1.54	0.16	-1.66	0.07	-	-
C9	C6	-0.90	0.13	-0.99	0.13	-	-
C10	C2	2.93	0.18	2.81	0.17	-	-

*Only the sum of the individual $^1D_{CH}$ of a methylene group is observable.

4.2 α-Santonin

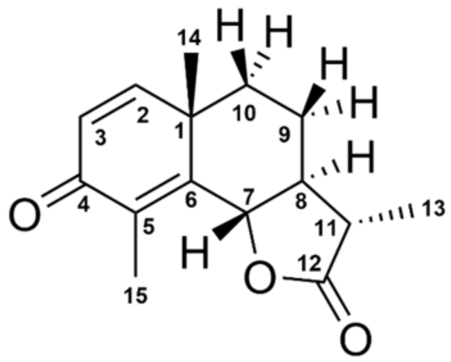

Figure S34: (-)-α-Santonin with numbered atom positions.

Table S10: Assignment of α-santonin in $CDCl_3$ at 700 MHz (^{1}H freq.) and 300 K.[48]

C	H	δC / ppm	δH / ppm
1	-	41.33	-
2	2	154.18	6.68
3	3	125.91	6.24
4	-	186.28	-
5	-	128.78	-
6	-	150.89	-
7	7	81.38	4.78
8	8	53.53	1.80
9	9a	23.09	2.02
9	9b	23.09	1.68
10	10a	37.86	1.89
10	10b	37.86	1.50
11	11	41.01	2.41
12	-	177.53	-
13	13	12.48	1.26
14	14	25.14	1.32
15	15	10.89	2.12

Table S11: Scalar couplings ($^1J_{CH}$) of α-santonin in $CDCl_3$ at 700 MHz (^1H freq.) and 300 K.

		CLIP CLAP		F_1-cpl	
**C**	**H**	**$^1J_{CH}$ / Hz**	**Δ^1J_{CH} / Hz**	**$^1J_{CH}$ / Hz**	**Δ^1J_{CH} / Hz**
1	-	-	-	-	-
2	2	159.83	0.05	159.83	0.03
3	3	165.61	0.06	165.64	0.05
4	-	-	-	-	-
5	-	-	-	-	-
6	-	-	-	-	-
7	7	146.95	0.05	146.88	0.07
8	8	127.70	0.15	126.85	0.06
9	9a	131.75	0.34	255.98*	0.09
9	9b	124.81	0.19	255.86*	0.08
10	10a	131.51	0.12	259.79*	0.06
10	10b	128.60	0.12	259.84*	0.08
11	11	125.93	0.11	125.96	0.06
12	-	-	-	-	-
13	13	128.62	0.04	128.65	0.02
14	14	129.45	0.04	129.40	0.01
15	15	129.24	0.04	129.22	0.02

*Only the sum of the individual $^1J_{CH}$ of a methylene group is observable.

Table S12: Total couplings ($^1T_{CH}$) of α-santonin in 9.41 % (w/w) DPPS-PBDG/$CDCl_3$ at 700 MHz (^1H freq.) and 300 K.

		CLIP CLAP		F_1-cpl	
**C**	**H**	**$^1T_{CH}$ / Hz**	**Δ^1T_{CH} / Hz**	**$^1T_{CH}$ / Hz**	**Δ^1T_{CH} / Hz**
1	-	-	-	-	-
2	2	151.65	0.38	152.48	0.09
3	3	116.59	0.18	116.73	0.11
4	-	-	-	-	-
5	-	-	-	-	-
6	-	-	-	-	-
7	7	231.51	0.73	230.25	0.20
8	8	209.34	1.54	211.10	0.16
9	9a	120.66	2.10	322.73*	0.25
9	9b	199.47	1.83	320.46*	0.32

10	10a	135.40	1.98	344.08*	0.22
10	10b	211.14	3.11	344.82*	0.34
11	11	199.35	0.58	199.86	0.25
12	-	-	-	-	-
13	13	135.24	0.43	135.27	0.04
14	14	104.89	0.35	105.00	0.18
15	15	128.86	0.17	128.80	0.05

*Only the sum of the individual ${}^1T_{CH}$ of a methylene group is observable.

Table S13: RDCs (${}^1D_{CH}$/${}^1D_{CC}$) of α-santonin calculated from the scalar couplings (${}^1J_{CH}$) in isotropic $CDCl_3$ (**Table S11**) and the total couplings (${}^1T_{CH}$) in 9.41 % (w/w) DPPS-PBDG/$CDCl_3$ (**Table S12**) at 700 MHz (^{1}H freq.) and 300 K.

		**CLIP CLAP**		**F_1-cpl**	
**C**	**X=H,C**	**${}^1D_{CX}$ / Hz**	**$\Delta{}^1D_{CX}$ / Hz**	**${}^1D_{CX}$ / Hz**	**$\Delta{}^1D_{CX}$ / Hz**
C1	-	-	-	-	-
C2	H2	-4.09	0.43	-3.67	0.12
C3	H3	-24.51	0.24	-24.45	0.17
C4	-	-	-	-	-
C5	-	-	-	-	-
C6	-	-	-	-	-
C7	H7	42.28	0.79	41.68	0.28
C8	H8	40.82	1.69	42.12	0.22
C9	H9a	-5.55	2.44	-	-
C9	H9b	37.33	2.02	-	-
C10	H10a	1.95	2.10	-	-
C10	H10b	41.27	3.23	-	-
C11	H11	36.71	0.69	36.95	0.31
C12	-	-	-	-	-
C13	C11	-0.95	0.47	-0.95	0.06
C14	C1	3.52	0.39	3.49	0.20
C15	C5	0.05	0.21	0.06	0.07

4.3 Artemisinin

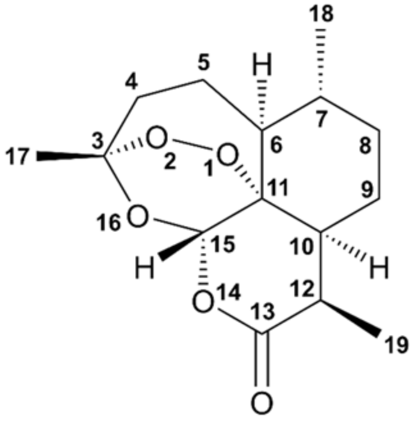

Figure S35: Artemisinin with numbered atom positions.

Table S14: Assignment of artemisinin in $CDCl_3$ at 700 MHz (^{1}H freq.) and 300 K.[49]

C	H	δC / ppm	δH / ppm
O-1	-	-	-
O-2	-	-	-
3	-	105.37	-
4	4a	35.90	2.43
4	4b	35.90	2.05
5	5a	24.85	2.00
5	5b	24.85	1.48
6	6	50.07	1.38
7	7	37.52	1.42
8	8a	33.60	1.78
8	8b	33.60	1.06
9	9a	23.39	1.88
9	9b	23.39	1.08
10	10	44.97	1.76
11	-	79.49	-
12	12	32.88	3.39
13	-	172.03	-
O-14	-	-	-
15	15	93.70	5.86
O-16	-	-	-
17	17	25.18	1.44
18	18	19.81	1.00
19	19	12.54	1.20

Table S15: Scalar couplings ($^1J_{CH}$) of artemisinin in $CDCl_3$ at 700 MHz (^1H freq.) and 300 K.

		CLIP CLAP		F1-cpl	
**C**	**H**	**$^1J_{CH}$ / Hz**	**Δ^1J_{CH} / Hz**	**$^1J_{CH}$ / Hz**	**Δ^1J_{CH} / Hz**
O-1	-	-	-	-	-
O-2	-	-	-	-	-
3	-	-	-	-	-
4	4a	129.16	0.12	256.10*	0.03
4	4b	126.94	0.10	256.12*	0.04
5	5a	128.70	0.09	256.79*	0.14
5	5b	127.21	0.63	256.78*	0.22
6	6	129.97	0.25	131.59	0.09
7	7	125.28	0.33	125.20	0.05
8	8a	129.65	0.14	255.21*	0.06
8	8b	125.40	0.25	255.28*	0.05
9	9a	130.83	0.17	255.89*	0.08
9	9b	124.62	0.16	255.82*	0.09
10	10	130.80	0.21	131.08	0.08
11	-	-	-	-	-
12	12	126.29	0.08	126.35	0.05
13	-	-	-	-	-
O-14	-	-	-	-	-
15	15	174.26	0.05	174.32	0.02
O-16	-	-	-	-	-
17	17	129.41	0.05	129.43	0.02
18	18	125.70	0.06	125.68	0.03
19	19	128.56	0.06	128.55	0.02

*Only the sum of the individual $^1J_{CH}$ of a methylene group is observable.

Table S16: Total couplings ($^{1}T_{CH}$) of artemisinin in 9.61 % (w/w) DPPS-PBDG/$CDCl_3$ at 700 MHz (^{1}H freq.) and 300 K.

		**CLIP CLAP**		**F_1-cpl**	
**C**	**H**	**$^{1}T_{CH}$ / Hz**	**$\Delta^{1}T_{CH}$ / Hz**	**$^{1}T_{CH}$ / Hz**	**$\Delta^{1}T_{CH}$ / Hz**
O-1	-	-	-	-	-
O-2	-	-	-	-	-
3	-	-	-	-	-
4	4a	197.79	0.23	325.82*	0.09
4	4b	128.17	0.51	325.94*	0.09
5	5a	93.30	2.05	290.96*	0.10
5	5b	194.87	1.87	290.69*	0.16
6	6	188.66	1.55	191.94	0.21
7	7	187.87	0.84	188.56	0.16
8	8a	131.92	0.84	313.47*	0.08
8	8b	174.57	3.15	314.27*	0.42
9	9a	122.17	1.09	302.96*	0.64
9	9b	176.90	2.56	303.57*	0.20
10	10	187.58	0.62	188.16	0.12
11	-	-	-	-	-
12	12	144.88	0.23	144.96	0.05
13	-	-	-	-	-
O-14	-	-	-	-	-
15	15	195.05	0.46	195.00	0.05
O-16	-	-	-	-	-
17	17	137.40	0.17	137.30	0.04
18	18	141.15	0.21	141.06	0.07
19	19	132.69	0.21	132.87	0.04

Only the sum of the individual $^{1}T_{CH}$ of a methylene group is observable.

Table S17: RDCs ($^{1}D_{CH}$/$^{1}D_{CC}$) of artemisinin calculated from the scalar couplings ($^{1}J_{CH}$) in isotropic $CDCl_3$ (**Table S15**) and the total couplings ($^{1}T_{CH}$) in 9.61 % (w/w) DPPS-PBDG/$CDCl_3$ (**Table S16**) at 700 MHz (^{1}H freq.) and 300 K.

		CLIP CLAP		F_1-cpl	
**C**	**X=H,C**	**$^{1}D_{CX}$ / Hz**	**$\Delta^{1}D_{CX}$ / Hz**	**$^{1}D_{CX}$ / Hz**	**$\Delta^{1}D_{CX}$ / Hz**
O-1	-	-	-	-	-
O-2	-	-	-	-	-
3	-	-	-	-	-
4	4a	34.32	0.36	-	-
4	4b	0.62	0.60	-	-
5	5a	-17.70	2.14	-	-
5	5b	33.83	2.50	-	-
6	6	29.34	1.81	30.18	0.30
7	7	31.29	1.18	31.68	0.22
8	8a	1.14	0.99	-	-
8	8b	24.58	3.39	-	-
9	9a	-4.33	1.26	-	-
9	9b	26.14	2.72	-	-
10	10	28.39	0.83	28.54	0.20
11	-	-	-	-	-
12	12	9.29	0.31	9.30	0.10
13	-	-	-	-	-
O-14	-	-	-	-	-
15	15	10.39	0.51	10.34	0.06
O-16	-	-	-	-	-
17	17	-1.14	0.22	-1.13	0.05
18	18	-2.21	0.27	-2.20	0.10
19	19	-0.59	0.27	-0.62	0.06

4.4 (+)-Vincamine

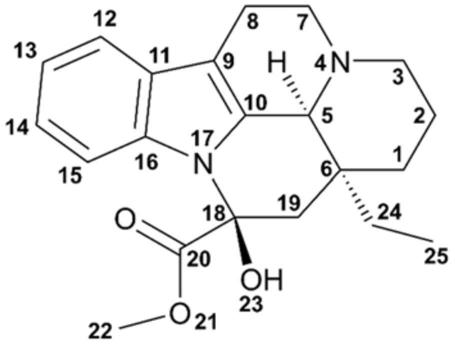

Figure S36: (+)-Vincamine with numbered atom positions.

Table S18: Assignment of vincamine in $CDCl_3$ at 700 MHz (^{1}H freq.) and 300 K.[50]

C	H	δC / ppm	δH / ppm
1	1a	24.98	1.70
1	1b	24.98	1.50
2	2a	20.61	1.77
2	2b	20.61	1.39
3	3a	44.59	2.66
3	3b	44.59	2.54
N-4	-	-	-
5	5	59.21	3.96
6	-	35.16	-
7	7a	51.01	3.37
7	7b	51.01	3.31
8	8a	16.78	3.00
8	8b	16.78	2.59
9	-	105.87	-
10	-	130.93	-
11	-	128.88	-
12	12	118.51	7.49
13	13	120.33	7.13
14	14	121.79	7.13
15	15	110.35	7.10
16	-	134.18	-
N-17	-	-	-
18	-	81.88	-
19	19a	44.35	2.23
19	19b	44.35	2.14
20	-	174.40	-
O21	-	-	-
22	22	54.30	3.83
O23	OH-23	-	4.61
24	24a	28.91	2.28
24	24b	28.91	1.48
25	25	7.57	0.92

Table S19: Scalar couplings ($^{1}J_{CH}$) of vincamine in $CDCl_3$ at 700 MHz (^{1}H freq.) and 300 K.

		**CLIP CLAP**		**F_1-cpl**	
**C**	**H**	**$^{1}J_{CH}$ / Hz**	**$\Delta^{1}J_{CH}$ / Hz**	**$^{1}J_{CH}$ / Hz**	**$\Delta^{1}J_{CH}$ / Hz**
1	1a	130.29	0.69	259.44*	0.10
1	1b	131.25	0.16	259.60*	0.09
2	2a	129.83	0.41	257.92*	0.10
2	2b	129.77	0.60	257.95*	0.15
3	3a	137.60	0.19	267.97*	0.08
3	3b	130.42	0.11	267.98*	0.06
N-4	-	-	-	-	-
5	5	139.92	0.10	139.84	0.05
6	-	-	-	-	-
7	7a	138.53	0.12	275.05*	0.04
7	7b	136.59	0.10	275.03*	0.07
8	8a	127.59	0.13	257.52*	0.03
8	8b	129.90	0.11	257.53*	0.08
9	-	-	-	-	-
10	-	-	-	-	-
11	-	-	-	-	-
12	12	158.57	0.05	158.57	0.04
13	13	158.94	0.05	158.79	0.04
14	14	159.92	0.08	159.77	0.07
15	15	159.52	0.11	159.81	0.04
16	-	-	-	-	-
N-17	-	-	-	-	-
18	-	-	-	-	-
19	19a	125.99	0.07	257.93*	0.06
19	19b	131.90	0.06	257.93*	0.05
20	-	-	-	-	-
O21	-	-	-	-	-
22	22	148.73	0.02	148.69	0.01
O23	OH-23	-	-	-	-
24	24a	126.71	0.12	252.24*	0.04
24	24b	125.51	0.16	252.28*	0.04
25	25	125.25	0.05	125.31	0.01

*Only the sum of the individual $^{1}J_{CH}$ of a methylene group is observable.

Table S20: Total couplings ($^1T_{CH}$) of vincamine in 9.86 % (w/w) DPPS-PBDG/$CDCl_3$ at 700 MHz (^{1}H freq.) and 300 K.

		CLIP CLAP		F_1-cpl	
**C**	**H**	$^1T_{CH}$ / Hz	Δ^1T_{CH} / Hz	$^1T_{CH}$ / Hz	Δ^1T_{CH} / Hz
1	1a	90.53	2.05	230.31*	0.54
1	1b	136.83	1.43	229.26*	1.67
2	2a	80.90	1.91	282.21*	0.95
2	2b	198.79	2.13	282.76*	0.95
3	3a	97.91	1.36	195.41*	1.09
3	3b	98.10	1.12	195.99*	0.46
N-4	-	-	-	-	-
5	5	216.31	0.60	217.23	0.20
6	-	-	-	-	-
7	7a	93.47	0.83	306.88*	0.20
7	7b	212.60	1.38	306.88*	0.20
8	8a	174.44	0.99	332.10*	0.20
8	8b	155.73	0.77	331.04*	0.23
9	-	-	-	-	-
10	-	-	-	-	-
11	-	-	-	-	-
12	12	148.24	0.49	149.61	0.13
13	13	123.62	0.25	124.75	0.08
14	14	108.19	0.24	109.41	0.09
15	15	151.84	0.31	151.81	0.08
16	-	-	-	-	-
N-17	-	-	-	-	-
18	-	-	-	-	-
19	19a	197.22	1.21	342.47*	0.24
19	19b	143.05	1.30	341.06*	0.16
20	-	-	-	-	-
O21	-	-	-	-	-
22	22	153.37	0.37	153.51	0.03
O23	OH-23	-	-	-	-
24	24a	103.54	0.97	271.05*	0.12
24	24b	166.09	0.89	269.97*	0.21
25	25	123.55	0.37	123.76	0.03

*Only the sum of the individual $^1T_{CH}$ of a methylene group is observable.

Table S21: RDCs ($^1D_{CH}$/$^1D_{CC}$) of vincamine calculated from the scalar couplings ($^1J_{CH}$) in isotropic $CDCl_3$ (**Table S19**) and the total couplings ($^1T_{CH}$) in 9.86 % (w/w) DPPS-PBDG/$CDCl_3$ (**Table S20**) at 700 MHz (^{1}H freq.) and 300 K.

		**CLIP CLAP**		**F_1-cpl**	
**C**	**X=H,C**	**$^1D_{CX}$ / Hz**	**Δ^1D_{CX} / Hz**	**$^1D_{CX}$ / Hz**	**Δ^1D_{CX} / Hz**
C1	H1a	-19.88	2.74	-	-
C1	H1b	2.79	1.59	-	-
C2	H2a	-24.47	2.33	-	-
C2	H2b	34.51	2.73	-	-
C3	H3a	-19.84	1.54	-	-
C3	H3b	-16.16	1.23	-	-
N-4	-	-	-	-	-
C5	H5	38.20	0.70	38.70	0.26
C6	-	-	-	-	-
C7	H7a	-22.53	0.95	-	-
C7	H7b	38.01	1.48	-	-
C8	H8a	23.42	1.12	-	-
C8	H8b	12.92	0.88	-	-
C9	-	-	-	-	-
C10	-	-	-	-	-
C11	-	-	-	-	-
C12	H12	-5.16	0.53	-4.48	0.17
C13	H13	-17.66	0.30	-17.02	0.12
C14	H14	-25.86	0.33	-25.18	0.15
C15	H15	-3.84	0.43	-4.00	0.13
C16	-	-	-	-	-
N-17	-	-	-	-	-
C18	-	-	-	-	-
C19	H19a	35.61	1.28	-	-
C19	H19b	5.58	1.36	-	-
C20	-	-	-	-	-
O21	-	-	-	-	-
C22	O-21	-0.66	0.39	-0.69	0.04
O23	OH-23	-	-	-	-
C24	H24a	-11.59	1.10	-	-
C24	H24b	20.29	1.05	-	-
C25	C24	0.24	0.42	0.22	0.04

4.5 (-)-Galantamine

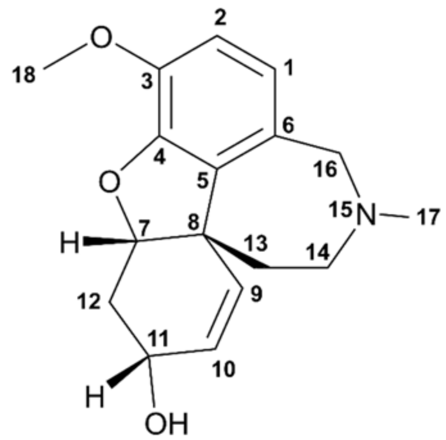

Figure S37: (-)-galantamine with numbered atom positions.

Table S22: Assignment of (-)-galantamine in $CDCl_3$ at 700 MHz (^{1}H freq.) and 300 K.[51,52]

C	H	δC / ppm	δH / ppm
1	1	122.25	6.65
2	2	111.33	6.68
3	-	144.32	-
4	-	145.90	-
5	-	133.00	-
6	-	128.39	-
7	7	88.71	4.63
8	-	48.15	-
9	9	126.64	6.06
10	10	127.84	6.03
11	11	62.03	4.16
12	12a	29.94	2.70
12	12b	29.94	2.03
13	13a	33.54	2.11
13	13b	33.54	1.63
14	14a	53.73	3.32
14	14b	53.73	3.10
N-15	-	-	-
16	16a	60.39	4.14
16	16b	60.39	3.74
17	17	41.74	2.44
18	18	55.93	3.85

Table S23: Scalar couplings ($^{1}J_{CH}$) of (-)-galantamine in $CDCl_3$ at 700 MHz (^{1}H freq.) and 300 K.

		CLIP CLAP		F_1-cpl	
**C**	**H**	**$^{1}J_{CH}$ / Hz**	**$\Delta^{1}J_{CH}$ / Hz**	**$^{1}J_{CH}$ / Hz**	**$\Delta^{1}J_{CH}$ / Hz**
1	1	159.26	0.06	159.12	0.06
2	2	158.49	0.05	158.21	0.05
3	-	-	-	-	-
4	-	-	-	-	-
5	-	-	-	-	-
6	-	-	-	-	-
7	7	150.70	0.05	150.65	0.07
8	-	-	-	-	-
9	9	159.27	0.06	159.58	0.18
10	10	161.79	0.05	161.76	0.05
11	11	147.34	0.05	147.41	0.04
12	12a	133.03	0.10	255.25*	0.04
12	12b	122.17	0.12	255.19*	0.04
13	13a	128.16	0.13	253.67*	0.13
13	13b	125.15	0.10	253.50*	0.08
14	14a	133.14	0.14	268.94*	0.07
14	14b	136.16	0.19	269.16*	0.05
N-15	-	-	-	-	-
16	16a	132.37	0.07	268.88*	0.42
16	16b	135.83	0.07	268.83*	0.47
17	17	134.08	0.03	134.30	0.09
18	18	144.36	0.02	144.40	0.01

*Only the sum of the individual $^{1}J_{CH}$ of a methylene group is observable.

Table S24: Total couplings ($^{1}T_{CH}$) of (-)-galantamine in 9.32 % (w/w) DPPS-PBDG/$CDCl_3$ at 700 MHz (^{1}H freq.) and 300 K.

		CLIP CLAP		F1-cpl	
**C**	**H**	**$^{1}T_{CH}$ / Hz**	**$\Delta^{1}T_{CH}$ / Hz**	**$^{1}T_{CH}$ / Hz**	**$\Delta^{1}T_{CH}$ / Hz**
1	1	154.24	0.31	154.47	0.08
2	2	182.51	0.13	183.09	0.07
3	-	-	-	-	-
4	-	-	-	-	-
5	-	-	-	-	-
6	-	-	-	-	-
7	7	194.51	0.33	195.89	0.25
8	-	-	-	-	-
9	9	163.61	0.83	164.12	0.59
10	10	205.47	0.59	203.51	0.14
11	11	155.91	0.34	155.93	0.11
12	12a	100.47	0.80	221.99*	0.15
12	12b	121.58	0.79	222.38*	0.09
13	13a	164.40	0.95	317.27*	0.14
13	13b	152.88	0.62	316.29*	0.16
14	14a	165.15	0.77	293.74*	0.10
14	14b	128.93	0.56	294.17*	0.18
N-15	-	-	-	-	-
16	16a	158.30	0.56	296.50*	0.43
16	16b	138.05	0.68	296.07*	0.91
17	17	134.39	0.12	134.41	0.13
18	18	142.11	0.17	141.74	0.03

*Only the sum of the individual $^{1}T_{CH}$ of a methylene group is observable.

Table S25: RDCs ($^1D_{CH}$/$^1D_{CC}$) of (-)-galantamine calculated from the scalar couplings ($^1J_{CH}$) in isotropic $CDCl_3$ (**Table S23**) and the total couplings ($^1T_{CH}$) in 9.32 % (w/w) DPPS-PBDG/$CDCl_3$ (**Table S24**) at 700 MHz (^{1}H freq.) and 300 K.

		**CLIP CLAP**		**F_1-cpl**	
**C**	**X=H,C**	**$^1D_{CX}$ / Hz**	**Δ^1D_{CX} / Hz**	**$^1D_{CX}$ / Hz**	**Δ^1D_{CX} / Hz**
C1	H1	-2.51	0.37	-2.33	0.14
C2	H2	12.01	0.18	12.44	0.12
C3	-	-	-	-	-
C4	-	-	-	-	-
C5	-	-	-	-	-
C6	-	-	-	-	-
C7	H7	21.91	0.38	22.62	0.31
C8	-	-	-	-	-
C9	H9	2.17	0.89	2.27	0.77
C10	H10	21.84	0.64	20.87	0.19
C11	H11	4.29	0.39	4.26	0.14
C12	H12a	-16.28	0.90	-	-
C12	H12b	-0.29	0.91	-	-
C13	H13a	18.12	1.08	-	-
C13	H13b	13.86	0.71	-	-
C14	H14a	16.01	0.91	-	-
C14	H14b	-3.62	0.75	-	-
N-15	-	-	-	-	-
C16	H16a	12.96	0.63	-	-
C16	H16b	1.11	0.74	-	-
C17	N-15	-	-	-	-
C18	O	-	-	-	-

5. Orientational properties of analytes (RDC@hotFCHT fits)

The following parameters are used in the **Table S26** – **Table S36**:

#RDC – number of RDCs used for analysis

RMSD – Root mean square deviation

Q – Quality factor as defined by Cornilescu et al.[53] The measure of RDC-fitting quality used in this work

CN – Condition number of the coefficient matrix

Da – Axial component of the Saupe tensor

Dr – Rhombic component of the Saupe tensor

R – Rhombicity of the Alignment tensor

GDO – Generalized degree of order

Euler angles α, β, γ – Euler angles to describe the relative orientation of the principle axis frame of the order tensor to the molecule-fixed frame (ZY'Z'' convention).

All RDC fits are performed using individual weighting of all RDCs based on their absolute error (error weighting) to ensure that the RDCs that can be determined with a higher confidence contribute more to the determination of the alignment tensor.[54,55] This has some implications for the condition numbers reported here, since not all RDC vector orientations contribute equally, and the condition number is higher than if an equal weighting of all RDCs is employed.

5.1 (-)-Isopinocampheol

Table S26: Key parameters of the SVD-based RDC fitting (using RDC@hotFCHT) of (-)-IPC oriented in 10.67 w% DPPS-PBDG/$CDCl_3$ at 300 K and 700 MHz ^{1}H frequency. The $^1D_{CX}$ RDCs are determined via F_2-coupled CLIP/CLAP HSQC spectra.

Nucleus 1	Nucleus 2	$^1D_{CX,exp}$ / Hz	$^1D_{CX,calc}$ / Hz	$\Delta(^1D_{CX,exp} - ^1D_{CX,calc})$ / Hz
C1	H1	7.05 ± 0.34	6.80 ± 0.03	0.25
C2	H2	-2.08 ± 0.39	-2.35 ± 0.39	0.27
C3	H3	3.60 ± 0.25	3.53 ± 0.20	0.07
C4	H4s	5.48 ± 0.31	5.48 ± 0.32	0.00
C4	H4a	-3.51 ± 0.32	-3.65 ± 0.10	0.14
C5	H5	-11.29 ± 0.43	-11.72 ± 0.02	0.43
C7	H7s	9.21 ± 0.22	9.09 ± 0.33	0.12
C7	H7a	-2.60 ± 0.22	-2.64 ± 0.26	0.04
C8	C6	0.44 ± 0.16	0.55 ± 0.01	-0.11
C9	C6	0.26 ± 0.13	0.38 ± 0.01	-0.12
C10	C2	-0.84 ± 0.18	-0.80 ± 0.03	-0.04
#RDC			11	
RMSD / Hz			0.186	
Q			0.034	
CN			2.049	

Da (*10^{-4})	-3.215
Dr (*10^{-4})	-1.264
R	0.393
GDO (*10^{-4})	6.792
Euler α / °	246.22 ± 1.15
Euler β / °	34.31 ± 0.47
Euler γ / °	114.07 ± 1.88

Table S27: Key parameters of the SVD-based RDC fitting (using RDC@hotFCHT) of (-)-IPC oriented in 10.67 w% DPPS-PBDG/$CDCl_3$ at 300 K and 700 MHz ^1H frequency. The $^1D_{CX}$ RDCs are determined via a combination of F_1-coupled HSQC (CH and CH_3) and F_1-coupled HSQC spectra with MQ evolution (CH_2).

Nucleus 1	Nucleus 2	$^1D_{CX,exp}$ / Hz	$^1D_{CX,calc}$ / Hz	$\Delta(^1D_{CX,exp} - ^1D_{CX,calc})$ / Hz
C1	H1	6.53 ± 0.11	6.52 ± 0.04	0.01
C2	H2	-2.22 ± 0.12	-2.33 ± 0.18	0.11
C3	H3	3.52 ± 0.09	3.55 ± 0.14	-0.03
C4	H4s	5.13 ± 0.22	5.26 ± 0.14	-0.13
C4	H4a	-3.46 ± 0.18	-3.54 ± 0.06	0.08
C5	H5	-11.48 ± 0.18	-11.46 ± 0.02	-0.02
C7	H7s	9.16 ± 0.20	9.00 ± 0.17	0.16
C7	H7a	-2.78 ± 0.19	-2.59 ± 0.13	-0.19
C8	C6	0.48 ± 0.07	0.54 ± 0.01	-0.06
C9	C6	0.28 ± 0.13	0.38 ± 0.01	-0.10
C10	C2	-0.80 ± 0.17	-0.77 ± 0.02	-0.03
#RDC			11	
RMSD / Hz			0.102	
Q			0.019	
CN			2.525	
Da (*10^{-4})			-3.141	
Dr (*10^{-4})			-1.240	
R			0.395	
GDO (*10^{-4})			6.640	
Euler α / °			246.58 ± 0.50	
Euler β / °			34.60 ± 0.30	
Euler γ / °			113.40 ± 0.96	

The condition numbers reported for these fits of (-)-IPC differ since error weighting is applied during the RDC fitting process, and the experimental errors are not identical for both data sets used for enantiodifferentiation.

Table S28: Key parameters of the SVD-based RDC fitting (using RDC@hotFCHT) of (-)-IPC oriented in 10.62 w% DPPS-PBLG/$CDCl_3$ at 300 K and 700 MHz ^{1}H frequency. The $^1D_{CX}$ RDCs are determined via F_2-coupled CLIP/CLAP HSQC spectra.

Nucleus 1	Nucleus 2	$^1D_{CX,exp}$ / Hz	$^1D_{CX,calc}$ / Hz	$\Delta(^1D_{CX,exp} - ^1D_{CX,calc})$ / Hz
C1	H1	7.48 ± 0.46	7.15 ± 0.05	0.33
C2	H2	-4.57 ± 0.37	-5.00 ± 0.43	0.43
C3	H3	3.16 ± 0.26	3.15 ± 0.22	0.01
C4	H4s	2.40 ± 0.36	2.52 ± 0.37	-0.12
C4	H4a	-2.40 ± 0.42	-2.62 ± 0.13	0.22
C5	H5	-12.21 ± 0.43	-12.43 ± 0.01	0.22
C7	H7s	11.33 ± 0.23	11.22 ± 0.36	0.11
C7	H7a	-2.89 ± 0.22	-2.84 ± 0.28	-0.05
C8	C6	0.61 ± 0.30	0.75 ± 0.01	-0.14
C9	C6	0.22 ± 0.14	0.32 ± 0.02	-0.10
C10	C2	-0.73 ± 0.24	-0.69 ± 0.04	-0.04
#RDC			11	
RMSD / Hz			0.203	
Q			0.034	
CN			2.302	
Da (*10^{-4})			-3.503	
Dr (*10^{-4})			-1.378	
R			0.393	
GDO (*10^{-4})			7.401	
Euler α / °			60.20 ± 0.98	
Euler β / °			140.56 ± 0.47	
Euler γ / °			252.95 ± 1.76	

Table S29: Key parameters of the SVD-based RDC fitting (using RDC@hotFCHT) of (-)-IPC oriented in 10.62 w% DPPS-PBLG/$CDCl_3$ at 300 K and 700 MHz ^{1}H frequency. The $^1D_{CX}$ RDCs are determined via a combination of F_1-coupled HSQC (CH and CH_3) and F_1-coupled HSQC spectra with MQ evolution (CH_2).

Nucleus 1	Nucleus 2	$^1D_{CX,exp}$ / Hz	$^1D_{CX,calc}$ / Hz	$\Delta(^1D_{CX,exp} - ^1D_{CX,calc})$ / Hz
C1	H1	6.97 ± 0.11	6.99 ± 0.03	-0.02
C2	H2	-4.68 ± 0.11	-4.91 ± 0.17	0.23
C3	H3	3.07 ± 0.10	3.18 ± 0.12	-0.11
C4	H4s	2.17 ± 0.19	2.46 ± 0.13	-0.29
C4	H4a	-2.33 ± 0.19	-2.51 ± 0.05	0.18
C5	H5	-12.49 ± 0.19	-12.29 ± 0.02	-0.20
C7	H7s	11.22 ± 0.16	11.07 ± 0.16	0.15
C7	H7a	-3.07 ± 0.13	-2.76 ± 0.12	-0.31
C8	C6	0.66 ± 0.07	0.74 ± 0.01	-0.08
C9	C6	0.24 ± 0.11	0.32 ± 0.01	-0.08
C10	C2	-0.70 ± 0.15	-0.68 ± 0.02	-0.02
#RDC			11	
RMSD / Hz			0.179	
Q			0.030	
CN			2.287	
Da (*10^{-4})			-3.448	
Dr (*10^{-4})			-1.378	
R			0.400	
GDO (*10^{-4})			7.298	
Euler α / °			60.32 ± 0.40	
Euler β / °			140.53 ± 0.22	
Euler γ / °			253.18 ± 0.76	

5.2 α-Santonin

Table S30: Key parameters of the SVD-based RDC fitting (using RDC@hotFCHT) of α-santonin oriented in 9.41 w% DPPS-PBDG/$CDCl_3$ at 300 K and 700 MHz ^{1}H frequency. The $^1D_{CX}$ RDCs are determined via F2-coupled CLIP/CLAP HSQC spectra.

Nucleus 1	Nucleus 2	$^1D_{CX,exp}$ / Hz	$^1D_{CX,calc}$ / Hz	$\Delta(^1D_{CX,exp} - ^1D_{CX,calc})$ / Hz
C2	H2	-4.09 ± 0.43	-4.05 ± 1.54	-0.04
C3	H3	-24.51 ± 0.24	-24.55 ± 0.23	0.04
C7	H7	42.28 ± 0.79	41.72 ± 0.18	0.56
C8	H8	40.82 ± 1.69	40.31 ± 0.68	0.51
C9	H9a	-5.55 ± 2.44	-9.50 ± 2.80	3.95
C9	H9b	37.33 ± 2.02	40.32 ± 0.51	-2.99
C10	H10a	1.95 ± 2.10	0.84 ± 2.79	1.11
C10	H10b	41.27 ± 3.23	42.03 ± 1.33	-0.76
C11	H11	36.71 ± 0.69	36.55 ± 0.18	0.16
C13	C11	-0.95 ± 0.47	-1.02 ± 0.10	0.07
C14	C1	3.52 ± 0.39	3.61 ± 0.04	-0.09
C15	C5	0.05 ± 0.21	-0.19 ± 0.17	0.24
#RDC			12	
RMSD / Hz			1.501	
Q			0.056	
CN			22.488	
Da (*10^{-4})			-9.230	
Dr (*10^{-4})			-5.192	
R			0.563	
GDO (*10^{-4})			20.535	
Euler α / °			101.75 ± 23.77	
Euler β / °			162.30 ± 14.77	
Euler γ / °			19.33 ± 21.36	

The condition number of the alignment tensor is quite high due to the RDC error weighting applied during the fitting process. The RDCs of the diastereotopic positions 9 and 10 show some differences between experimental and calculated RDCs due to asymmetric signals caused by strong coupling.

Table S31: Key parameters of the SVD-based RDC fitting (using RDC@hotFCHT) of α-santonin oriented in 9.41 w% DPPS-PBDG/$CDCl_3$ at 300 K and 700 MHz ^{1}H frequency. The $^1D_{CX}$ RDCs are determined via a combination of F_1-coupled HSQC (CH and CH_3) and F_2-coupled CLIP/CLAP HSQC (CH_2) spectra.

Nucleus 1	Nucleus 2	$^1D_{CX,exp}$ / Hz	$^1D_{CX,calc}$ / Hz	$\Delta(^1D_{CX,exp} - ^1D_{CX,calc})$ / Hz
C2	H2	-3.67 ± 0.12	-3.65 ± 0.58	-0.02
C3	H3	-24.45 ± 0.17	-24.48 ± 0.13	0.03
C7	H7	41.68 ± 0.28	42.52 ± 0.11	-0.84
C8	H8	42.12 ± 0.22	41.53 ± 0.17	0.59
C9	H9a	-5.55 ± 2.44	-9.02 ± 1.13	3.47
C9	H9b	37.33 ± 2.02	41.30 ± 0.09	-3.97
C10	H10a	1.95 ± 2.10	-0.03 ± 1.05	1.98
C10	H10b	41.27 ± 3.23	44.30 ± 0.47	-3.03
C11	H11	36.95 ± 0.31	36.68 ± 0.08	0.27
C13	C11	-0.95 ± 0.06	-1.09 ± 0.01	0.14
C14	C1	3.52 ± 0.20	3.71 ± 0.01	-0.19
C15	C5	0.05 ± 0.07	-0.20 ± 0.06	0.25
#RDC			12	
RMSD / Hz			1.874	
Q			0.070	
CN			17.915	
Da (*10^{-4})			-9.699	
Dr (*10^{-4})			-5.789	
R			0.597	
GDO (*10^{-4})			21.837	
Euler α / °			97.68 ± 2.03	
Euler β / °			160.26 ± 1.77	
Euler γ / °			17.11 ± 2.33	

The condition number of the alignment tensor is quite high due to the RDC error weighting applied during the fitting process. The RDCs of the diastereotopic positions 9 and 10 show some differences between experimental and calculated RDCs due to asymmetric signals caused by strong coupling.

5.3 Artemisinin

Table S32: Key parameters of the SVD-based RDC fitting (using RDC@hotFCHT) of artemisinin oriented in 9.61 w% DPPS-PBDG/$CDCl_3$ at 300 K and 700 MHz ^{1}H frequency. The $^1D_{CX}$ RDCs are determined via F_2-coupled CLIP/CLAP HSQC spectra.

Nucleus 1	Nucleus 2	$^1D_{CX,exp}$ / Hz	$^1D_{CX,calc}$ / Hz	$\Delta(^1D_{CX,exp} - ^1D_{CX,calc})$ / Hz
C4	H4a	34.32 ± 0.36	34.27 ± 0.08	0.05
C4	H4b	0.62 ± 0.60	0.28 ± 2.47	0.34
C5	H5a	-17.70 ± 2.14	-16.21 ± 1.63	-1.49
C5	H5b	33.83 ± 2.50	34.43 ± 0.77	-0.60
C6	H6	29.34 ± 1.81	28.59 ± 0.45	0.75
C7	H7	31.29 ± 1.18	31.06 ± 0.73	0.23
C8	H8a	1.14 ± 0.99	1.19 ± 1.21	-0.05
C8	H8b	24.58 ± 3.39	26.92 ± 0.75	-2.34
C9	H9a	-4.33 ± 1.26	-2.81 ± 2.33	-1.52
C9	H9b	26.14 ± 2.72	29.29 ± 0.35	-3.15
C10	H10	28.39 ± 0.83	28.34 ± 0.71	0.05
C12	H12	9.29 ± 0.31	9.10 ± 1.11	0.19
C15	H15	10.39 ± 0.51	10.95 ± 1.06	-0.56
C17	C3	-1.14 ± 0.22	-1.22 ± 0.06	0.08
C18	C7	-2.21 ± 0.27	-2.43 ± 0.16	0.22
C19	C12	-0.59 ± 0.27	-0.47 ± 0.19	-0.12
#RDC			16	
RMSD / Hz			1.157	
Q			0.056	
CN			10.092	
Da (*10^{-4})			-7.715	
Dr (*10^{-4})			-3.156	
R			0.409	
GDO (*10^{-4})			16.370	
Euler α / °			235.29 ± 30.01	
Euler β / °			176.31 ± 1.17	
Euler γ / °			214.65 ± 30.82	

The RDCs of the diastereotopic positions 5, 8, and 9 show some differences between experimental and calculated RDCs due to asymmetric signals caused by strong coupling.

5.4 (+)-Vincamine

Table S33: Key parameters of the SVD-based RDC fitting (using RDC@hotFCHT) of (+)-vincamine oriented in 9.86 w% DPPS-PBDG/$CDCl_3$ at 300 K and 700 MHz ^{1}H frequency. The $^1D_{CX}$ RDCs are determined via F_2-coupled CLIP/CLAP HSQC spectra.

Nucleus 1	Nucleus 2	$^1D_{CX,exp}$ / Hz	$^1D_{CX,calc}$ / Hz	$\Delta(^1D_{CX,exp} - ^1D_{CX,calc})$ / Hz
C1	H1a	-19.88 ± 2.74	-16.55 ± 0.55	-3.33
C1	H1b	2.79 ± 1.59	3.32 ± 0.20	-0.53
C2	H2a	-24.47 ± 2.33	-21.97 ± 0.45	-2.50
C2	H2b	34.51 ± 2.73	36.26 ± 0.48	-1.75
C3	H3a	-19.84 ± 1.54	-20.89 ± 0.89	1.05
C3	H3b	-16.16 ± 1.23	-13.23 ± 0.68	-2.93
C5	H5	38.20 ± 0.70	37.70 ± 0.58	0.50
C7	H7a	-22.53 ± 0.95	-21.24 ± 0.87	-1.29
C7	H7b	38.01 ± 1.48	36.71 ± 0.57	1.30
C8	H8a	23.42 ± 1.12	22.90 ± 0.75	0.52
C8	H8b	12.92 ± 0.88	13.66 ± 0.29	-0.74
C12	H12	-5.16 ± 0.53	-4.37 ± 0.54	-0.79
C13	H13	-17.66 ± 0.30	-17.79 ± 0.84	0.13
C14	H14	-25.86 ± 0.33	-26.06 ± 0.62	0.20
C15	H15	-3.84 ± 0.43	-4.25 ± 0.59	0.41
C19	H19a	35.61 ± 1.28	37.56 ± 0.52	-1.95
C19	H19b	5.58 ± 1.36	4.14 ± 0.31	1.44
#RDC			17	
RMSD / Hz			1.564	
Q			0.067	
CN			3.850	
Da (*10^{-4})			-8.615	
Dr (*10^{-4})			-4.566	
R			0.530	
GDO (*10^{-4})			18.959	
Euler α / °			38.65 ± 0.71	
Euler β / °			99.04 ± 0.66	
Euler γ / °			222.69 ± 0.48	

The RDCs of the diastereotopic positions show some differences between experimental and calculated RDCs due to asymmetric signals caused by strong coupling.

5.5 (-)-Galantamine

Table S34: Key parameters of the SVD-based RDC fitting (using RDC@hotFCHT) of (-)-galantamine oriented in 9.32 w% DPPS-PBDG/$CDCl_3$ at 300 K and 700 MHz ^{1}H frequency. The $^1D_{CX}$ RDCs are determined via F_2-coupled CLIP/CLAP HSQC spectra. RDCs are fit in a **single-conformer-single-tensor approach (SCST)** to the structure model of conformer 1.

Nucleus 1	Nucleus 2	$^1D_{CX,exp}$ / Hz	$^1D_{CX,calc}$ / Hz	$\Delta(^1D_{CX,exp} - ^1D_{CX,calc})$ / Hz
C1	H1	-2.51 ± 0.37	-2.45 ± 0.60	-0.06
C2	H2	12.01 ± 0.18	12.04 ± 0.41	-0.03
C7	H7	21.91 ± 0.38	22.19 ± 0.40	-0.28
C9	H9	2.17 ± 0.89	-1.08 ± 0.38	3.25
C10	H10	21.84 ± 0.64	20.04 ± 0.71	1.80
C11	H11	4.29 ± 0.39	3.71 ± 0.54	0.58
C12	H12a	-16.28 ± 0.90	-15.20 ± 0.48	-1.08
C12	H12b	-0.29 ± 0.91	-0.94 ± 0.26	0.65
C13	H13a	18.12 ± 1.08	17.99 ± 0.76	0.13
C13	H13b	13.86 ± 0.71	14.25 ± 0.63	-0.39
C14	H14a	16.01 ± 0.91	17.98 ± 0.79	-1.97
C14	H14b	-3.62 ± 0.75	-5.36 ± 0.30	1.74
C16	H16a	12.96 ± 0.63	12.80 ± 0.69	0.16
C16	H16b	1.11 ± 0.74	1.30 ± 0.75	-0.19
#RDC			14	
RMSD / Hz			1.280	
Q			0.099	
CN			4.991	
Da (*10^{-4})			9.160	
Dr (*10^{-4})			4.836	
R			0.528	
GDO (*10^{-4})			20.144	
Euler α / °			346.09	
Euler β / °			89.49	
Euler γ / °			293.27	

Table S35: Key parameters of the SVD-based RDC fitting (using RDC@hotFCHT) of (-)-galantamine oriented in 9.32 w% DPPS-PBDG/$CDCl_3$ at 300 K and 700 MHz ^{1}H frequency. The $^1D_{CX}$ RDCs are determined via F_2-coupled CLIP/CLAP HSQC spectra. RDCs are fit in a **single-conformer-single-tensor approach (SCST)** to the structure model of conformer 3.

Nucleus 1	Nucleus 2	$^1D_{CX,exp}$ / Hz	$^1D_{CX,calc}$ / Hz	$\Delta(^1D_{CX,exp} - ^1D_{CX,calc})$ / Hz
C1	H1	-2.51 ± 0.37	-4.71 ± 0.53	2.20
C2	H2	12.01 ± 0.18	11.78 ± 0.25	0.23
C7	H7	21.91 ± 0.38	20.85 ± 0.04	1.06
C9	H9	2.17 ± 0.89	4.86 ± 0.15	-2.69
C10	H10	21.84 ± 0.64	17.12 ± 0.55	4.72
C11	H11	4.29 ± 0.39	2.29 ± 0.26	2.00
C12	H12a	-16.28 ± 0.90	-1.65 ± 0.25	-14.63
C12	H12b	-0.29 ± 0.91	4.52 ± 0.19	-4.81
C13	H13a	18.12 ± 1.08	17.17 ± 0.54	0.95
C13	H13b	13.86 ± 0.71	17.61 ± 0.64	-3.75
C14	H14a	16.01 ± 0.91	17.36 ± 0.53	-1.35
C14	H14b	-3.62 ± 0.75	-4.18 ± 0.22	1.19
C16	H16a	12.96 ± 0.63	17.39 ± 0.35	-4.43
C16	H16b	1.11 ± 0.74	4.31 ± 0.70	-3.20
#RDC			14	
RMSD / Hz			4.816	
Q			0.371	
CN			5.369	
Da (*10^{-4})			8.351	
Dr (*10^{-4})			1.016	
R			0.122	
GDO (*10^{-4})			16.794	
Euler α / °			0.33	
Euler β / °			81.40	
Euler γ / °			289.70	

Table S36: Key parameters of the SVD-based RDC fitting (using RDC@hotFCHT) of (-)-galantamine oriented in 9.32 w% DPPS-PBDG/$CDCl_3$ at 300 K and 700 MHz ^{1}H frequency. The $^1D_{CX}$ RDCs are determined via F_2-coupled CLIP/CLAP HSQC spectra. RDCs are fit in a **multi-conformer-single-tensor approach (MCST)**. Data for the minimum of 96% conformer 1 and 4% conformer 3 is shown.

Nucleus 1	Nucleus 2	$^1D_{CX,exp}$ / Hz	$^1D_{CX,calc}$ / Hz	$\Delta(^1D_{CX,exp} - ^1D_{CX,calc})$ / Hz
C1	H1	-2.51 ± 0.37	-2.61 ± 0.61	0.10
C2	H2	12.01 ± 0.18	12.02 ± 0.42	-0.01
C7	H7	21.91 ± 0.38	22.43 ± 0.40	-0.52
C9	H9	2.17 ± 0.89	-0.26 ± 0.37	2.43
C10	H10	21.84 ± 0.64	19.79 ± 0.72	2.05
C11	H11	4.29 ± 0.39	4.09 ± 0.53	0.20
C12	H12a	-16.28 ± 0.90	-14.34 ± 0.46	-1.94
C12	H12b	-0.29 ± 0.91	-0.34 ± 0.25	0.05
C13	H13a	18.12 ± 1.08	17.86 ± 0.77	0.26
C13	H13b	13.86 ± 0.71	14.31 ± 0.64	-0.45
C14	H14a	16.01 ± 0.91	17.86 ± 0.80	-1.85
C14	H14b	-3.62 ± 0.75	-5.17 ± 0.30	1.55
C16	H16a	12.96 ± 0.63	12.65 ± 0.70	0.31
C16	H16b	1.11 ± 0.74	1.25 ± 0.75	-0.14
#RDC			14	
RMSD / Hz			1.207	
Q			0.093	
CN			5.043	
Da (*10^{-4})			9.172	
Dr (*10^{-4})			4.857	
R			0.529	
GDO (*10^{-4})			20.182	
Euler α / °			346.20	
Euler β / °			89.56	
Euler γ / °			293.43	

6. Input structures/coordinates

The generation of the input structures is described in **SI chapter 2.6**.

6.1 α-santonin

```
36
Coordinates from ORCA-job santonin_alpha_strucopt
C3     -11.46825118855859     -4.99227184413330     -1.25897699068663
C4     -10.95596049112607     -3.74219816088344     -0.69227170567463
C5      -9.62446265153952     -3.25383625297630     -1.14592149633371
C6      -8.95174269276592     -3.95045480169340     -2.08857004493686
C1      -9.49661621028913     -5.20333766420097     -2.77675543940063
C2     -10.79990324547133     -5.65657334825436     -2.20430550273822
C7      -7.56936646109213     -3.65959215056493     -2.59247007577174
C8      -6.62151613890942     -4.82215979751603     -2.27111170834387
C9      -7.04743936831913     -6.06336455753441     -3.03344165411919
C10     -8.49247158749303     -6.39815887380182     -2.63728803611557
O       -6.88289743975031     -2.49792272424048     -2.06228291467198
C12     -5.54462260606112     -2.74111579537225     -2.04939130787599
C11     -5.26543938739842     -4.16522484719948     -2.52217129020053
O      -11.61819973092531     -3.12661085678850      0.14692906543155
C14     -9.76365168788596     -4.91241490732213     -4.28265908736471
C15     -9.13304483457039     -1.99390760584418     -0.47137920179922
C13     -4.05532333723946     -4.80359866155785     -1.85449586304763
O       -4.75889411551253     -1.89990975348117     -1.69410907062335
H3     -12.42212596975484     -5.34156281154657     -0.87628682709790
H2     -11.20596846745832     -6.57487118490098     -2.62278982673145
H7      -7.60419387385265     -3.51489833517221     -3.67749801452561
H8      -6.70447677061913     -5.02243452886755     -1.19360103856215
H9b     -6.95729866951083     -5.89406505184558     -4.11200844063307
H9a     -6.40141704288185     -6.91202935840581     -2.79063055322039
H10b    -8.50059922603386     -6.72629444147492     -1.59249400228093
H10a    -8.87255100204871     -7.23249326063213     -3.23383313858094
H11     -5.09810984304788     -4.09387304633725     -3.60750697081383
H14a    -8.84418854142285     -4.71798102654897     -4.83553296136036
H14b   -10.24852711012493     -5.77665977960190     -4.74308864261041
H14c   -10.42210463604692     -4.04847371274613     -4.39491959479788
H15a    -9.93342783349273     -1.57488581479245      0.13593372924637
H15b    -8.28239436329260     -2.19469090882225      0.18409390593201
H15c    -8.81178789624538     -1.24440284979068     -1.19323008499316
H13a    -3.15239290223655     -4.21851780390854     -2.04097868824938
H13b    -3.89280335168437     -5.81093728454585     -2.24522926712056
H13c    -4.19645032533772     -4.87537719669516     -0.77254925932741
```

6.2 (+)-Vincamine

```
52
Coordinates from ORCA-job vincamine_crystalstruc_strucopt
C25     4.52894289591072    10.11092748116439    15.41639382981972
H25a    3.49853988779354     9.91498037232270    15.72388035001401
H25b    4.96013783385861    10.79774966127712    16.15046436619897
H25c    5.08146425375748     9.16978941322276    15.49050603077300
C24     4.60342683549626    10.72415888520443    14.01666732307534
H24b    5.63411848909648    11.04076769036195    13.81625570968184
H24a    4.00553702393519    11.63840411809880    13.99271595314452
C6      4.15588351299672     9.81432787577845    12.84473063332803
C1      2.75350312588270     9.22138770899203    13.07956499658508
H1a     2.52352653137660     8.49669029817298    12.29542436059441
H1b     2.73552792640892     8.66205028335386    14.01848534978458
C2      1.68247860836907    10.31446211524545    13.06685784833358
H2a     1.82063651539293    11.00992124977905    13.90094629528718
H2b     0.69179018622545     9.86531551759527    13.19046243201810
C3      1.71917128266861    11.07353694227360    11.74377003384815
H3b     1.41801455928268    10.38443693151569    10.93781266830899
H3a     0.99728669725406    11.89598175826235    11.74768401682326
N4      3.04596411618384    11.65778192701917    11.49812829637654
C7      3.10348142708029    12.37858035680405    10.21596946230263
H7b     4.08941375699063    12.85107117382991    10.15693655318732
H7a     2.36172642957812    13.18146234718684    10.24188465129223
C8      2.88576956976533    11.50158286960323     8.95259598857277
H8a     1.81477643383377    11.36683682941386     8.75514468016909
H8b     3.28532282422749    12.03448938935791     8.08212994711654
C9      3.54184536347229    10.16687538013867     9.13845226266863
C11     3.66375731094160     9.02620407753168     8.26971354415480
C12     3.33662390412443     8.78363518520253     6.92905875383600
H12     2.87364707139139     9.55973237271094     6.32710474978414
C13     3.60914690117341     7.53526638703299     6.38186770589530
H13     3.35742765066076     7.33647169011431     5.34489727504108
C14     4.20565000029989     6.52064489870252     7.15219773586563
H14     4.40182622835986     5.55274637080576     6.70250076552239
C15     4.54603361652073     6.73217999072151     8.48537694433608
H15     4.99142816338697     5.93697994994458     9.07138557419461
C16     4.27570770292846     7.98875979655358     9.02684208477237
N17     4.53420098692851     8.49610901244828    10.29408788653284
C10     4.08090869207265     9.80414823477904    10.33654757972741
C5      4.13749245618336    10.66626711637246    11.55582314799666
H5      5.07371710253722    11.24200669002518    11.53767138540852
C19     5.23691163018354     8.72610334473358    12.64093878201996
H19a    6.20526342805675     9.22861397591248    12.55504302740581
```

```
H19b     5.30170127515358     8.07794488060512     13.51608993922618
C18      5.03858937259710     7.77574522216437     11.43256205834643
O        4.11920617311500     6.74164078415696     11.74121109689603
H23      4.63462885240748     5.96186219409691     12.00571163171485
C20      6.41021143577591     7.08879061487973     11.13933035754516
O        6.59791869566417     5.96361032945351     11.55277279674402
O        7.39841864133256     7.71464366230796     10.50817255976924
C22      7.32727251177673     9.02674072999060      9.89519152432075
H22a     6.96514106023703     9.77444311117684     10.59593053971866
H22b     8.35585588498328     9.24840027874841      9.62244599700992
H22c     6.70234755237211     8.99184564185056      9.00611801190696
```

6.3 (-)-Galantamine

Conformer 1 (highest Boltzmann population)

```
42
Coordinates from ORCA-job galantamine_crest20240503_conf01 E -940.589636182638
C9       0.681214      1.753368      1.088044
C10     -0.193968      2.722018      1.363433
C11     -1.207465      3.243597      0.377392
C12     -0.838147      2.861932     -1.056272
C7      -0.407553      1.415571     -1.170718
C8       0.748954      0.996877     -0.225234
C3      -1.705564     -1.758271     -0.037923
C2      -1.005474     -2.814923      0.549237
C1       0.347660     -2.675390      0.881923
C16      2.503713     -1.347530      1.076195
C14      3.335327      0.624912     -0.161253
C13      2.110282      1.154268     -0.927017
C4      -0.990668     -0.581907     -0.270585
C5       0.343887     -0.441716      0.069204
C6       1.048211     -1.487003      0.668389
C18     -3.760759     -2.965759     -0.188363
C17      3.464348     -1.619244     -1.147706
N15      3.420501     -0.818101      0.068045
O       -1.538937      0.545143     -0.822064
O       -3.015484     -1.769988     -0.412680
O       -2.535836      2.800830      0.727458
H11     -1.262554      4.332096      0.457225
H7      -0.137937      1.166485     -2.202011
H9       1.364922      1.423960      1.864965
H10     -0.215602      3.161976      2.357643
H12b    -0.006061      3.488045     -1.395885
H12a    -1.681739      3.059930     -1.723145
H2      -1.500438     -3.759020      0.739619
```

H1	0.862342	-3.523332	1.324342
H16b	2.870076	-2.333177	1.381915
H16a	2.582813	-0.703499	1.959477
H14a	3.414061	1.117988	0.812298
H14b	4.227664	0.917579	-0.725566
H13b	2.274668	2.219429	-1.129526
H13a	2.053935	0.660081	-1.902268
H18a	-3.798092	-3.212039	0.876808
H18b	-4.765900	-2.760706	-0.551350
H18c	-3.333817	-3.805376	-0.744891
H17a	3.709285	-2.653248	-0.888849
H17b	4.249559	-1.238324	-1.806254
H17c	2.521332	-1.634416	-1.715509
HO	-2.586372	1.852080	0.535847

Conformer 2 (similar to conformer 1)

42

Coordinates from ORCA-job galantamine_crest20240503_conf02 E -940.587032817197

C9	0.728339	1.778703	1.099088
C10	-0.130135	2.747888	1.420492
C11	-1.229359	3.232729	0.510943
C12	-0.975958	2.815651	-0.938131
C7	-0.540203	1.370837	-1.050555
C8	0.692868	0.984373	-0.192896
C3	-1.706903	-1.784734	0.279604
C2	-0.950078	-2.805274	0.856619
C1	0.416934	-2.646796	1.086534
C16	2.566013	-1.294502	1.043271
C14	3.280142	0.632308	-0.331093
C13	1.991021	1.128369	-1.008884
C4	-1.034329	-0.611298	-0.055731
C5	0.326525	-0.448199	0.174021
C6	1.083867	-1.460332	0.757518
C18	-3.553470	-1.790004	-1.223313
C17	3.344406	-1.646725	-1.239370
N15	3.393632	-0.800253	-0.054652
O	-1.630493	0.502141	-0.585286
O	-3.060016	-1.951291	0.117279
O	-2.518489	2.789154	0.984445
H11	-1.289546	4.322444	0.565648
H7	-0.355326	1.096332	-2.094028
H9	1.480708	1.479546	1.822742
H10	-0.070406	3.217058	2.399618
H12b	-0.180911	3.439559	-1.360292
H12a	-1.872957	2.988928	-1.538864

H2	-1.451147	-3.732370	1.115412
H1	0.976653	-3.467263	1.525674
H16b	2.964573	-2.264974	1.356718
H16a	2.707088	-0.616607	1.892510
H14a	3.441112	1.164172	0.611634
H14b	4.118259	0.904323	-0.982064
H13b	2.128860	2.187597	-1.257223
H13a	1.858642	0.603220	-1.960334
H18a	-3.037395	-2.478292	-1.899565
H18b	-4.612993	-2.038388	-1.186277
H18c	-3.424661	-0.764160	-1.570740
H17a	2.359495	-1.682152	-1.729891
H17b	3.611748	-2.670297	-0.962224
H17c	4.074140	-1.290327	-1.971219
HO	-2.568062	1.831893	0.843130

Conformer 3 (unique conformer)

42

Coordinates from ORCA-job galantamine_crest20240503_conf03 E -940.578496770374

C9	0.409669	1.600266	1.165207
C10	-0.081874	2.836626	1.236225
C11	-0.714649	3.498157	0.040611
C12	-1.539069	2.456474	-0.724971
C7	-0.742522	1.201338	-1.079976
C8	0.462334	0.834748	-0.143254
C3	-1.518102	-2.280917	-0.382333
C2	-0.706441	-3.246664	0.210837
C1	0.559228	-2.921619	0.702437
C16	2.437343	-1.268342	1.159662
C14	3.072392	0.769768	-0.089121
C13	1.797515	1.123447	-0.874548
C4	-1.013225	-0.980860	-0.456359
C5	0.228249	-0.647868	0.066847
C6	1.047552	-1.611463	0.652058
C18	-3.874515	-1.990576	-0.360704
C17	3.551337	-1.454206	-1.003690
N15	3.336876	-0.643787	0.187935
O	-1.663375	0.064010	-1.042742
O	-2.727334	-2.643538	-0.927318
O	-1.520041	4.624071	0.407120
H11	0.054311	3.925888	-0.611683
H7	-0.376242	1.257371	-2.108373
H9	0.835112	1.129741	2.045517
H10	-0.059933	3.400109	2.165733
H12b	-1.970263	2.903700	-1.623500
H12a	-2.367784	2.153388	-0.076258

H2	-1.074415	-4.266366	0.261373
H1	1.177111	-3.704061	1.132924
H16b	2.911568	-2.188010	1.516810
H16a	2.371169	-0.597038	2.022203
H14a	3.072435	1.299351	0.868696
H14b	3.928858	1.153687	-0.653673
H13b	1.838753	2.192157	-1.115454
H13a	1.788562	0.589299	-1.830187
H18a	-3.838285	-0.913124	-0.530102
H18b	-4.743155	-2.414071	-0.862843
H18c	-3.933261	-2.195901	0.712670
H17a	2.650675	-1.601196	-1.619363
H17b	3.914760	-2.442713	-0.708845
H17c	4.314697	-0.982674	-1.628128
HO	-2.233796	4.323858	0.986880

Conformer 4 (similar to conformer 3)

42

Coordinates from ORCA-job galantamine_crest20240503_conf04 E -940.580860718937

C9	0.517017	1.673306	1.253408
C10	0.095586	2.934790	1.335223
C11	-0.579511	3.611117	0.170587
C12	-1.511719	2.603419	-0.513425
C7	-0.807296	1.303927	-0.899411
C8	0.446477	0.895305	-0.046301
C3	-1.665305	-2.140164	-0.151207
C2	-0.855939	-3.140594	0.390067
C1	0.451317	-2.855218	0.804766
C16	2.423960	-1.284777	1.129275
C14	3.049402	0.724819	-0.171247
C13	1.738576	1.124071	-0.871425
C4	-1.110911	-0.859391	-0.249008
C5	0.170698	-0.576066	0.184753
C6	0.991310	-1.572147	0.719004
C18	-3.517554	-3.602017	-0.511735
C17	3.386262	-1.519687	-1.100261
N15	3.279644	-0.696453	0.096430
O	-1.772676	0.211800	-0.772873
O	-2.942184	-2.299819	-0.603107
O	-1.292733	4.788625	0.564604
H11	0.165547	3.981464	-0.541823
H7	-0.513390	1.325237	-1.952548
H9	0.971758	1.190622	2.112323
H10	0.203771	3.508515	2.252371
H12b	-1.983115	3.059068	-1.387239

H12a	-2.305456	2.355406	0.198947
H2	-1.226686	-4.154082	0.478801
H1	1.060377	-3.664498	1.197054
H16b	2.885358	-2.223019	1.454270
H16a	2.448704	-0.612432	1.993554
H14a	3.133751	1.258095	0.780750
H14b	3.879739	1.073654	-0.794646
H13b	1.807065	2.188124	-1.126273
H13a	1.643453	0.583932	-1.819158
H18a	-2.949477	-4.325751	-1.103867
H18b	-3.567027	-3.938025	0.528300
H18c	-4.524327	-3.510720	-0.914390
H17a	3.735881	-2.518164	-0.822274
H17b	4.121144	-1.078483	-1.778777
H17c	2.442545	-1.642031	-1.654184
HO	-1.980159	4.540697	1.198442

7. CREST run for galantamine

The following is an excerpt of the CREST[25–27] output file for galantamine. Parts where information is left out are marked with […]. The command line input, the thresholds used, and the four relevant conformers (conf. 1 = conf. 2, conf. 3 = conf. 4) are highlighted with different colors. Conformers 5 to 12, all inside a 3 kcal energy window, are presented to show that they do not show a relevant (1 % being the cut-off) Boltzmann population.

```
       ==============================================
       |                                            |
       |                 C R E S T                  |
       |                                            |
       |  Conformer-Rotamer Ensemble Sampling Tool  |
       |          based on the GFN methods          |
       |             P.Pracht, S.Grimme             |
       |          Universitaet Bonn, MCTC           |
       ==============================================
       Version 2.12,   Thu 19. Mai 16:32:32 CEST 2022
  Using the xTB program. Compatible with xTB version 6.4.0
[…]

Command line input:
 > crest galantamine_crystalstruc_1258428_strucopt.xyz --gfn2 --alpb chloroform
-T 4

  -gfn2 : Use of GFN2-xTB requested.
  --alpb chloroform : implicit solvation
  -T 4 (CPUs/Threads selected)

 -------------------------

[…]

input  file name : crest_rotamers_6.xyz
 output file name : crest_rotamers_7.xyz
 number of atoms                :   42
 number of points on xyz files  :   259
 RMSD threshold                 :   0.1250
 Bconst threshold               :   0.0100
 population threshold           :   0.0500
 conformer energy window  /kcal :   6.0000
 # fragment in coord            :     1
 # bonds in reference structure :    45
 number of reliable points      :   259
 reference state Etot :  -62.2764487000000
 number of doubles removed by rot/RMSD         :           1
 total number unique points considered further :         258
       Erel/kcal        Etot weight/tot  conformer     set   degen     origin
       1   0.000   -62.27645    0.05425    0.48805       1       9     mtd3
       2   0.000   -62.27645    0.05425                                mtd11
       3   0.000   -62.27645    0.05424                                mtd11
       4   0.000   -62.27645    0.05424                                mtd5
       5   0.000   -62.27645    0.05423                                mtd9
       6   0.000   -62.27645    0.05423                                hor
       7   0.000   -62.27645    0.05422                                mtd3
       8   0.000   -62.27645    0.05421                                mtd8
       9   0.001   -62.27645    0.05418                                mtd9
      10   0.108   -62.27628    0.04523    0.40636       2       9     mtd6
```

11	0.108	-62.27628	0.04521				mtd2
12	0.108	-62.27628	0.04521				md4
13	0.108	-62.27628	0.04520				mtd9
14	0.108	-62.27628	0.04519				mtd9
15	0.108	-62.27628	0.04519				mtd11
16	0.109	-62.27628	0.04518				mtd8
17	0.110	-62.27627	0.04509				mtd1
18	0.113	-62.27627	0.04486				mtd10
19	1.496	-62.27406	0.00435	0.03913	3	9	mtd8
20	1.496	-62.27406	0.00435				mtd1
21	1.496	-62.27406	0.00435				mtd14
22	1.496	-62.27406	0.00435				mtd8
23	1.497	-62.27406	0.00435				md6
24	1.497	-62.27406	0.00435				md8
25	1.497	-62.27406	0.00435				md8
26	1.497	-62.27406	0.00435				mtd6
27	1.497	-62.27406	0.00434				md5
28	1.935	-62.27337	0.00208	0.01869	4	9	md8
29	1.935	-62.27337	0.00208				mtd9
30	1.935	-62.27337	0.00208				mtd1
31	1.935	-62.27337	0.00208				mtd1
32	1.935	-62.27337	0.00208				hor
33	1.935	-62.27337	0.00208				hor
34	1.935	-62.27336	0.00208				md7
35	1.935	-62.27336	0.00208				mtd4
36	1.936	-62.27336	0.00207				mtd10
37	2.258	-62.27285	0.00120	0.00479	5	4	hor
38	2.258	-62.27285	0.00120				mtd5
39	2.265	-62.27284	0.00119				mtd7
40	2.266	-62.27284	0.00119				mtd7
41	2.449	-62.27255	0.00087	0.00523	6	6	mtd4
42	2.449	-62.27255	0.00087				gc
43	2.449	-62.27255	0.00087				mtd5
44	2.449	-62.27255	0.00087				mtd7
45	2.449	-62.27255	0.00087				gc
46	2.450	-62.27255	0.00087				gc
47	2.583	-62.27233	0.00070	0.00555	7	8	mtd4
48	2.583	-62.27233	0.00070				mtd12
49	2.583	-62.27233	0.00070				mtd5
50	2.583	-62.27233	0.00070				mtd6
51	2.584	-62.27233	0.00070				mtd13
52	2.585	-62.27233	0.00069				mtd1
53	2.588	-62.27233	0.00069				mtd8
54	2.588	-62.27232	0.00069				mtd6
55	2.595	-62.27231	0.00068	0.00341	8	5	mtd12
56	2.595	-62.27231	0.00068				mtd9
57	2.596	-62.27231	0.00068				mtd12
58	2.596	-62.27231	0.00068				mtd1
59	2.596	-62.27231	0.00068				mtd12
60	2.712	-62.27213	0.00056	0.00447	9	8	mtd7
61	2.713	-62.27213	0.00056				mtd10
62	2.713	-62.27213	0.00056				mtd4
63	2.713	-62.27213	0.00056				mtd13
64	2.713	-62.27213	0.00056				mtd9
65	2.713	-62.27212	0.00056				md6
66	2.713	-62.27212	0.00056				md6
67	2.714	-62.27212	0.00056				mtd13
68	2.881	-62.27186	0.00042	0.00252	10	6	mtd2
69	2.882	-62.27186	0.00042				md5
70	2.882	-62.27186	0.00042				mtd11
71	2.883	-62.27186	0.00042				md5
72	2.883	-62.27185	0.00042				mtd12
73	2.884	-62.27185	0.00042				mtd9
74	2.915	-62.27180	0.00040	0.00278	11	7	mtd6
75	2.915	-62.27180	0.00040				mtd9

76	2.915	-62.27180	0.00040				md4
77	2.915	-62.27180	0.00040				mtd8
78	2.916	-62.27180	0.00040				mtd9
79	2.916	-62.27180	0.00040				mtd1
80	2.916	-62.27180	0.00040				mtd13
81	2.965	-62.27172	0.00037	0.00328	12	9	mtd8
82	2.965	-62.27172	0.00037				mtd5
83	2.966	-62.27172	0.00037				mtd5
84	2.966	-62.27172	0.00037				mtd8
85	2.966	-62.27172	0.00037				mtd10
86	2.966	-62.27172	0.00036				mtd5
87	2.967	-62.27172	0.00036				mtd10
88	2.968	-62.27172	0.00036				mtd11
89	2.969	-62.27172	0.00036				mtd5

[…]

8. Download NMR raw data

The NMR raw data can be downloaded using the following DOI:
https://doi.org/10.5281/zenodo.14170603

The archive contains NMR raw data of the compounds synthesized (**2-12**) and the NMR raw data used for the determination of RDCs (isotropic and anisotropic data).

9. Author Contributions

J.R.†: Conceptualization (NMR, RDC): Lead; Data acquisition (NMR, RDC): Lead; Data interpretation (NMR, RDC): Lead; Formal analysis (NMR, RDC): Lead; Original Draft: Lead; Review and Editing: Lead

M.G.†: Conceptualization (synthesis): Lead; Synthesis: Lead; Acquisition of analytic data (synthesis): Lead; Interpretation of analytic data (synthesis): Lead; Original Draft: Supporting; Review and Editing: Supporting

C.M.T.: Project Administration: Lead; Conceptualization (total project): Lead; Data interpretation (total project): Supporting; Original Draft: Supporting; Review and Editing: Supporting

† Both authors contributed equally

10. Literature

[1] G. R. Fulmer, A. J. M. Miller, N. H. Sherden, H. E. Gottlieb, A. Nudelman, B. M. Stoltz, J. E. Bercaw, K. I. Goldberg, *Organometallics* **2010**, *29*, 2176–2179.
[2] R. K. Harris, E. D. Becker, S. M. Cabral De Menezes, P. Granger, R. E. Hoffman, K. W. Zilm, *Pure Appl. Chem.* **2008**, *80*, 59–84.
[3] Z. Grubisic, P. Rempp, H. Benoit, *J. Polym. Sci. [B]* **1967**, *5*, 753–759.
[4] H. C. Benoit, *J. Polym. Sci. Part B Polym. Phys.* **1996**, *34*, 1703–1704.
[5] M. Hirschmann, C. Merten, C. M. Thiele, *Soft Matter* **2021**, *17*, 2849–2856.
[6] P. Trigo ‐ Mouriño, C. Merle, M. R. M. Koos, B. Luy, R. R. Gil, *Chem. – Eur. J.* **2013**, *19*, 7013–7019.
[7] L. Castañar, E. Sistaré, A. Virgili, R. T. Williamson, T. Parella, *Magn. Reson. Chem.* **2015**, *53*, 115–119.
[8] A. Enthart, J. C. Freudenberger, J. Furrer, H. Kessler, B. Luy, *J. Magn. Reson.* **2008**, *192*, 314–322.
[9] C. M. Thiele, W. Bermel, *J. Magn. Reson.* **2012**, *216*, 134–143.
[10] K. E. Kövér, K. Fehér, *J. Magn. Reson.* **2004**, *168*, 307–313.
[11] G. Kummerlöwe, S. Schmitt, B. Luy, *Open Spectrosc. J.* **2010**, *4*, 16–27.
[12] C. M. Thiele, *Eur. J. Org. Chem.* **2008**, *2008*, 5673–5685.
[13] L. Verdier, P. Sakhaii, M. Zweckstetter, C. Griesinger, *J. Magn. Reson.* **2003**, *163*, 353–359.
[14] A. Marx, V. Schmidts, C. M. Thiele, *Magn. Reson. Chem.* **2009**, *47*, 734–740.
[15] A. Navarro-Vázquez, R. R. Gil, K. Blinov, *J. Nat. Prod.* **2018**, *81*, 203–210.
[16] F. Neese, *WIREs Comput. Mol. Sci.* **2012**, *2*, 73–78.
[17] F. Neese, *WIREs Comput. Mol. Sci.* **2022**, *12*, e1606.
[18] A. D. Becke, *J. Chem. Phys.* **1993**, *98*, 5648–5652.
[19] A. D. Becke, *J. Chem. Phys.* **1993**, *98*, 1372–1377.
[20] C. Lee, W. Yang, R. G. Parr, *Phys. Rev. B* **1988**, *37*, 785–789.
[21] R. Krishnan, J. S. Binkley, R. Seeger, J. A. Pople, *J. Chem. Phys.* **1980**, *72*, 650–654.
[22] T. Clark, J. Chandrasekhar, G. W. Spitznagel, P. V. R. Schleyer, *J. Comput. Chem.* **1983**, *4*, 294–301.
[23] E. Caldeweyher, C. Bannwarth, S. Grimme, *J. Chem. Phys.* **2017**, *147*, 034112.
[24] V. Barone, M. Cossi, *J. Phys. Chem. A* **1998**, *102*, 1995–2001.
[25] S. Grimme, *J. Chem. Theory Comput.* **2019**, *15*, 2847–2862.
[26] P. Pracht, F. Bohle, S. Grimme, *Phys. Chem. Chem. Phys.* **2020**, *22*, 7169–7192.
[27] P. Pracht, S. Grimme, C. Bannwarth, F. Bohle, S. Ehlert, G. Feldmann, J. Gorges, M. Müller, T. Neudecker, C. Plett, S. Spicher, P. Steinbach, P. A. Wesołowski, F. Zeller, *J. Chem. Phys.* **2024**, *160*, 114110.
[28] S. Ehlert, M. Stahn, S. Spicher, S. Grimme, *J. Chem. Theory Comput.* **2021**, *17*, 4250–4261.
[29] T. A. Halgren, *J. Comput. Chem.* **1996**, *17*, 490–519.
[30] S. Parsons, H. D. Flack, T. Wagner, *Acta Crystallogr. Sect. B Struct. Sci. Cryst. Eng. Mater.* **2013**, *69*, 249–259.
[31] C. R. Groom, I. J. Bruno, M. P. Lightfoot, S. C. Ward, *Acta Crystallogr. Sect. B Struct. Sci. Cryst. Eng. Mater.* **2016**, *72*, 171–179.
[32] H. M. Greenblatt, G. Kryger, T. Lewis, I. Silman, J. L. Sussman, *FEBS Lett.* **1999**, *463*, 321–326.
[33] M. W. Campbell, J. S. Compton, C. B. Kelly, G. A. Molander, *J. Am. Chem. Soc.* **2019**, *141*, 20069–20078.

[34] X. Xia, P. Toy, *Synlett* **2015**, *26*, 1737–1743.
[35] Y.-S. Hon, C.-F. Lee, R.-J. Chen, P.-H. Szu, *Tetrahedron* **2001**, *57*, 5991–6001.
[36] F. Chalier, Y. Berchadsky, J.-P. Finet, G. Gronchi, S. Marque, P. Tordo, *J. Phys. Chem.* **1996**, *100*, 4323–4330.
[37] X. Chen, H. Wu, R. Yu, H. Zhu, Z. Wang, *J. Org. Chem.* **2021**, *86*, 8987–8996.
[38] L. Monnereau, D. Sémeril, D. Matt, L. Toupet, *Polyhedron* **2013**, *51*, 70–74.
[39] A. T. Breshears, A. C. Behrle, C. L. Barnes, C. H. Laber, G. A. Baker, J. R. Walensky, *Polyhedron* **2015**, *100*, 333–343.
[40] M. Hayashi, T. Matsuura, I. Tanaka, H. Ohta, Y. Watanabe, *Org. Lett.* **2013**, *15*, 628–631.
[41] V. L. Van Zyl, A. Muller, D. B. G. Williams, *Tetrahedron Lett.* **2018**, *59*, 918–921.
[42] W. A. R. Van Heeswijk, M. J. D. Eenink, J. Feijen, *Synthesis* **1982**, *1982*, 744–747.
[43] W. Liu, M. Zhu, J. Xiao, Y. Ling, H. Tang, *J. Polym. Sci. Part Polym. Chem.* **2016**, *54*, 3425–3435.
[44] W. D. Fuller, M. S. Verlander, M. Goodman, *Biopolymers* **1976**, *15*, 1869–1871.
[45] W. Zhao, Y. Gnanou, N. Hadjichristidis, *Polym. Chem.* **2015**, *6*, 6193–6201.
[46] J. M. Coxon, G. J. Hydes, P. J. Steel, *J. Chem. Soc. Perkin Trans. 2* **1984**, 1351.
[47] W.-R. Abraham, *Z. Für Naturforschung C* **1994**, *49*, 553–560.
[48] A. Ata, J. A. Nachtigall, *Z. Für Naturforschung C* **2004**, *59*, 209–214.
[49] G. Blaskó, G. A. Cordell, D. C. Lankin, *J. Nat. Prod.* **1988**, *51*, 1273–1276.
[50] E. Bonandi, F. Foschi, C. Marucci, G. Paladino, M. Luzzani, D. Passarella, *Phytochem. Rev.* **2021**, *20*, 343–365.
[51] B. M. Trost, F. D. Toste, *J. Am. Chem. Soc.* **2000**, *122*, 11262–11263.
[52] C. M. Halpin, C. Reilly, J. J. Walsh, *J. Chem. Educ.* **2010**, *87*, 1242–1243.
[53] G. Cornilescu, J. L. Marquardt, M. Ottiger, A. Bax, *J. Am. Chem. Soc.* **1998**, *120*, 6836–6837.
[54] C. M. Thiele, V. Schmidts, B. Böttcher, I. Louzao, R. Berger, A. Maliniak, B. Stevensson, *Angew. Chem.* **2009**, *121*, 6836–6840.
[55] V. Schmidts, *PhD Thesis*, Technical University of Darmstadt, May **2013**.
